# Altruism Can Proliferate through Population Viscosity despite High Random Gene Flow

**DOI:** 10.1371/journal.pone.0072043

**Published:** 2013-08-19

**Authors:** Roberto H. Schonmann, Renato Vicente, Nestor Caticha

**Affiliations:** 1 Department of Mathematics, University of California Los Angeles, Los Angeles, California, United States of America; 2 Department of Applied Mathematics, Instituto de Matemática e Estatstica, Universidade de São Paulo, São Paulo, SP, Brazil; 3 Departamento de Física Geral, Instituto de Física, Universidade de São Paulo, São Paulo, SP, Brazil; Universite de Sherbrooke, Canada

## Abstract

The ways in which natural selection can allow the proliferation of cooperative behavior have long been seen as a central problem in evolutionary biology. Most of the literature has focused on interactions between pairs of individuals and on linear public goods games. This emphasis has led to the conclusion that even modest levels of migration would pose a serious problem to the spread of altruism through population viscosity in group structured populations. Here we challenge this conclusion, by analyzing evolution in a framework which allows for complex group interactions and random migration among groups. We conclude that contingent forms of strong altruism that benefits equally all group members, regardless of kinship and without greenbeard effects, can spread when rare under realistic group sizes and levels of migration, due to the assortment of genes resulting only from population viscosity. Our analysis combines group-centric and gene-centric perspectives, allows for arbitrary strength of selection, and leads to extensions of Hamilton’s rule for the spread of altruistic alleles, applicable under broad conditions.

## Introduction

The evolution of cooperation and altruism are fundamental scientific challenges highlighted by their role in the major transitions in life’s history, when natural selection acted simultaneously on several competing levels [1]–[8]. In this context, the relevance of basic concepts, including group selection and Hamilton’s rule remain controversial [9]–[18]. Here we address these problems by studying a framework for evolution in group structured populations that incorporates inter- and intra-group competition and migration. Combining group-centric with gene-centric perspectives in a constructive group/kin selection approach, we build methodology that allows for the analysis of arbitrary non-linear fitness functions, resulting from complex multi-individual interactions across life cycles. We obtain the conditions for a rare social allele to invade the population. This is obtained in a mathematically rigorous way, by analyzing the stability of the equilibrium in which this allele is absent. This analysis is done for arbitrary strength of selection, but when selection is weak and groups are large the condition for invasion simplifies significantly into a form that is easy to apply and provides substantial intuition. In the case of linear fitness functions, the condition for invasion is identical to Hamilton’s rule, and it is natural to regard the more general non-linear cases as generalizations of that rule. Our results also show that one of the most widely used approaches to analyzing kin selection models, [19], [7](condition (6.7)), and [16](Box 6), yields incorrect results in some biologically relevant situations.

Our results reveal conditions that are biologically realistic and under which altruism can evolve when rare even with modest genetic relatedness in groups, without kin recognition or greenbeard effects (the altruistic acts benefit all group members equally). In this way we challenge a common understanding according to which inter-group selection favoring altruism could only override intra-group selection favoring selfishness under exceptional conditions, namely small group size and very low migration rates [13], [17], [20]–[28]. The issue is illustrated by quoting from the recent review [13], p.12: “For group selection to overcome selection within groups, less than one succesfully reproducing migrant may be exchanged per two populations per population lifetime.” The fact that this idea is still incorporated in mainstream evolutionary biology is illustrated by the theoretical considerations on pp. 11, 12 of the influential recent textbook [29], where one reads: “[…] for group selection to work populations must be isolated, such that individuals cannot migrate among them. Otherwise there would be nothing to stop the migration of selfish individuals […]. Once selfish individuals arrive, their genotype would soon spread. In nature groups are rarely isolated sufficiently to prevent such immigration.”

We identify the emphasis on linear public goods games in the literature, including most of the papers quoted in the previous paragraph, as having supported this belief in exceptionality. For these games, the condition for altruism to proliferate is Hamilton’s classical rule, requiring the relatedness in groups to exceed the ratio of cost to benefit for each altruistic act. Therefore, in this setting, altruism can only spread when either relatedness is large, or the cost/benefit ratio is low. And since relatedness is often low [28] (Table 8.3), [30], [31] (Tables 6.4 and 6.5), [32], [33] (Table 4.9), exceptionally low cost/benefit ratios are required, as observed for instance in [34]. In the absence of reasons to expect low cost/benefit ratios to be common, researchers whose intuition is molded by the linear public goods game are naturally led to the belief that altruism could only spread through population viscosity in exceptional cases.

In contrast, we show that for iterated public goods games, in which altruists cooperate or not in each round based on previous outcomes [35], [36], altruism can spread even when cost/benefit ratios for each altruistic act are reasonably high, groups are large, selection is weak and migration rates are substantially larger than the inverse of group size (high gene flow, realistically low relatedness). This result corrects [36], who predicted that large group size would not allow cooperation to spread when rare in this model. For species that live in groups, several vital group activities repeat themselves periodically and behavior changes as feedback is obtained from previous iterations. The iterated public goods game that we study is therefore often more realistic than a simple one shot public goods game. A proper analysis of this model fills therefore an important gap in the literature. (To illustrate the fact that the incorrect conclusion from [36] is still incorporated in the literature, we refer the reader to, e.g., Section 4.5 of the textbook [37] and p. 359 of the very recent textbook [29]).

To obtain our result in the case of weak selection and large groups we show that in the absence of selection, when groups are large, the fraction of group members that are close relatives of a randomly chosen individual has a non-Gaussian distribution with a fatter tail. As a consequence, even when altruistic alleles are rare in the population, they have a significant probability of concentrating in some groups, accruing substantial reproductive gains through multi-individual synergy.

### The Two-Level Fisher-Wright Framework

**Figure 1:**
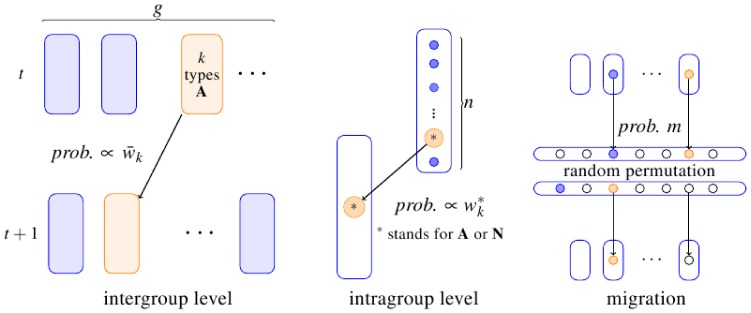
Diagram of the 2lFW process. **(Left) FW intergroup competition:** Each group in the new generation independently descends from a group in the previous generation, with probabilities proportional to group average fitness 
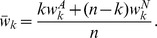
 **(Center) FW Intragroup competition:** If a group descends from a group with 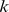 types A, then it will have 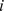 types A with probability 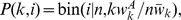 where the binomial probability 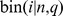 is the probability of 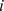 successes in 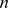 independent trials, each with probability 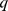 of success. **(Right) Migration:** Once the new 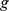 groups have been formed according to the two-level competition process, a random fraction 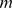 of the individuals migrates. Migrants are randomly shuffled. **Note:** The assignment of relative fitness to the groups in the fashion done above is a necessary and sufficient condition [68] for individuals in the parental generation to have each an expected number of offspring proportional to their personal relative fitness.

When members of a species live in groups, their reproductive success depends on the behavior of all group members. More efficient groups may grow faster and split, outcompeting the less efficient ones that die out. On the other hand, individuals may free ride on the cooperation of other members of their group, and in this way outcompete them. This picture is further complicated by migration among groups. The *Two-level Fisher-Wright framework with selection and migration* (2lFW) captures all these elements, in a simplified fashion. (In the last two paragraphs of this section we explain how it relates to the trait group framework and budding-viscosity models.) In 2lFW haploid individuals live in a large number 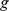 of groups of size 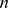, and are of two genetically determined phenotypic types, A or N. Generations do not overlap, reproduction is asexual and the type is inherited by the offspring (mutations will be considered briefly later). The relative fitness (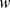) of a type A, and that of a type N, in a group that has 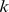 types A, are, respectively, 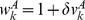 and 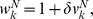 with the convention that 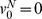, i.e., 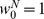. The quantities 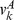 and 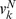 represent life-cycle payoffs derived from behavior, physiology, etc. The parameter 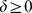 indicates the strength of selection. Figure 1 describes the creation of a new generation in the 2lFW through inter- and intra- group competition, followed by migration at rate 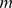.

Cases in which types A behave in some altruistic fashion are of particular interest [38]. Most of the literature concerns the very special case of a *linear* public goods game (PG), defined by
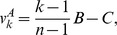


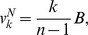
with 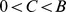, in which each type A cooperates, at a cost 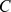 to herself, providing a benefit 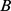 shared by the other members of her group. The need to consider more complex intra-group interactions and non-linear payoff functions is, nevertheless, well known [10], [14], [30], [35], [36], [39]–[49]. Non-linearities appear naturally whenever activities involve many group members simultaneously. They result from threshold phenomena, increasing returns to scale, saturation, etc. For instance, to hunt large prey may require a large minimum number of hunters, the likelihood of success may first increase rapidly with the number of hunters, but it may plateau when this number becomes very large. Allowing for the analysis of such synergistic multi-individual interactions and activities is a central feature of our approach, distinguishing it from theoretical frameworks based on pairwise interactions, or single actors benefiting a group [4], [50], [51].

The 2lFW framework can be seen as a generalization of the trait-group framework (see Sec. 2.3.2 of [5]), which corresponds to the case 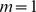. One can interpret 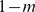 as a viscosity, or an assortment parameter. Because migration is completely random in 2lFW, this assortment represents a worst case scenario, abstracting away additional assortment caused by kin recognition, greenbeard effects, selective acceptance of migrants, joint migration of individuals, etc. We notice that, according to [52], the 2lFW can be classified as a Type II group selection model, as the intergroup competition component qualifies as an explicit group level event that is absent in a trait-group model. It is well known [35], [36], [43] that even when 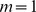 non-linearities in fitness functions allow for coexistence of cooperators and defectors. But under the strong altruism condition 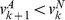 (meaning that each type A would be better off mutating into a type N), this is not the case [38], [53]. One of our goals is to determine the level of migration compatible with invasion by rare strong altruists.

The model with the population structure of 2lFW and PG payoffs was studied in [54] with the name “budding-viscosity model”. But, as the authors explained on p.1714, this name may not be appropriate in some applications. We propose the name “two-level Fisher-Wright framework with selection and migration” to denote a mathematical structure applicable to several demographic/reproductive biological systems, including group fissioning and budding, and admitting arbitrary payoff functions. This name also emphasizes that selection occurs at the individual and the group levels. In [54] motivation for the population structure is discussed in detail, references to previous work leading to it are provided (notably [55]) and an explanation of how it allows viscosity to increase relatedness without increasing in the same measure the competition among relatives (as is the case in an island model with inelastic group size [56]) is presented. The analysis in [54] relied on the assumptions of PG payoffs and weak selection (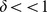). The paper [57] provides an alternative analysis, which depends on the same strong assumptions, in the section called “typical kin selection model”. This paper was a response to [58], where group selection was argued to be an important mechanism for the evolution of cooperation, and a multilevel selection model based on Moran’s model was introduced. In [57] it was argued that kin selection is a better tool for studying evolution in group structured populations. In the context of this debate, our analysis of 2lFW with non-linear fitness functions highlights the importance of combining group-centric with gene-centric perspectives, and shows that group selection can be an important force in evolution under realistic conditions. It also shows that mathematically rigorous analysis can be carried out, to a large extent, even when selection is strong, fitness functions are non-linear and migration rates are arbitrary. And it shows that one has to be very careful in applying mathematically non-rigorous methodology, as it can produce substantially incorrect results, even when selection is weak. We will see this when we observe that methods based on the approximation of regression coefficients by partial derivatives lead to such incorrect results in important examples.

## Results

### A Basic Example: Iterated Public Goods Game

Non-linearities in life-cycle payoffs can result from activities repeating themselves during a lifetime, and behavior being contingent on previous outcomes. A basic example is the iterated public goods game (IPG) [35], [36]. In IPG a PG is repeated an average of 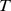 times in a life-cycle. We will suppose that types N never cooperate, while types A cooperate in the first round and later cooperate only if at least a fraction 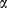 of group members cooperated in the previous round. The payoffs (see Figure S1 in File S1) are, therefore, given by
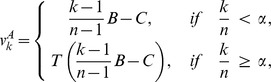


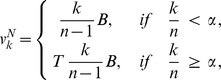
for constants 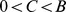, 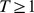 and 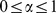 (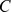 and 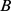 are costs and benefits in each iteration). Mathematically, this model generalizes the iterated prisoner dilemma and tit-for-tat, from the dyadic setting of [59] and [60] to the multi-individual setting. But while direct or indirect reciprocity requires the identification of individuals in the group, this is not the case here. The behavior of types A in the IPG can be triggered by individuals simply discontinuing cooperative behavior when previous cooperation produced negative feedback to them, for instance, when they received a negative payoff. In other words, allele A can predispose individuals to cooperate, but as they do it and obtain feedback from that behavior, they may continue it or discontinue it. The IPG is in this sense closely related to generalized reciprocity mechanisms [61], [62] with low cognitive requirements. (In generalized reciprocity models individuals interact in pairs, and generalized reciprocators help any other member of the group, but only in the first iteration, or when they were helped by some group member in the previous iteration. In contrast, in the IPG the actions involve several individuals simultaneously, but as in generalized reciprocity, individuals react to their own previous experience, without having to remember who did what.) Negative feedback from cooperation should occur if the fraction of group members that cooperated was less than 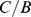, but not if it was larger than that threshold, since in the former case the payoff to a cooperator is negative, while in the latter case it is positive. This gives a special role to the value 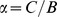.

When are types A altruistic in the IPG? There is more than one way in which the concept of altruism in the context of the trait-group framework has been defined [38]. These different definitions carry over to the 2lFW. A particularly simple concept is called in [38] the “multilevel interpretation” of altruism. That definition requires the two conditions 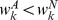, for all 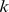, and 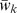 increasing in 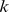. The first one means that types A are always worse off than types N in the same group, and the second one means that the more types A in a group, the better for the group. Both conditions are clearly always satisfied in the IPG.

There are nevertheless good arguments for considering other definitions of altruism [38]. A particularly appealing definition is called “focal-complement interpretation” in [38], and is often known as “mutation condition”, or “strong altruism”. Suppose that a type N mutated into a type A, everything else remaining unchanged. Would this cause a decrease in the fitness of the mutant? Would it cause an increase in the average fitness of the other members of the mutant’s group? Since the average fitness of the 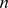 members of the mutant’s group increases with the mutation, the answer to the second question will be affirmative whenever the answer to the first question is affirmative. Therefore the condition for types A to be strongly altruistic is that 
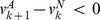
 for all 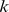. In File S1 (Section S8, last subsection) this condition is shown to be satisfied when 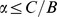 and to fail otherwise.

To sum up, if 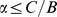, the behavior of types A is altruistic in the strong sense that each type A individual would increase its fitness if it behaved as a type N, everything else being equal, i.e., 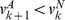. Moreover, types N always free ride and have greater fitness than types A in the same group, regardless of the values of 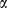 and 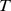, i.e., 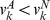.

**Figure 2:**
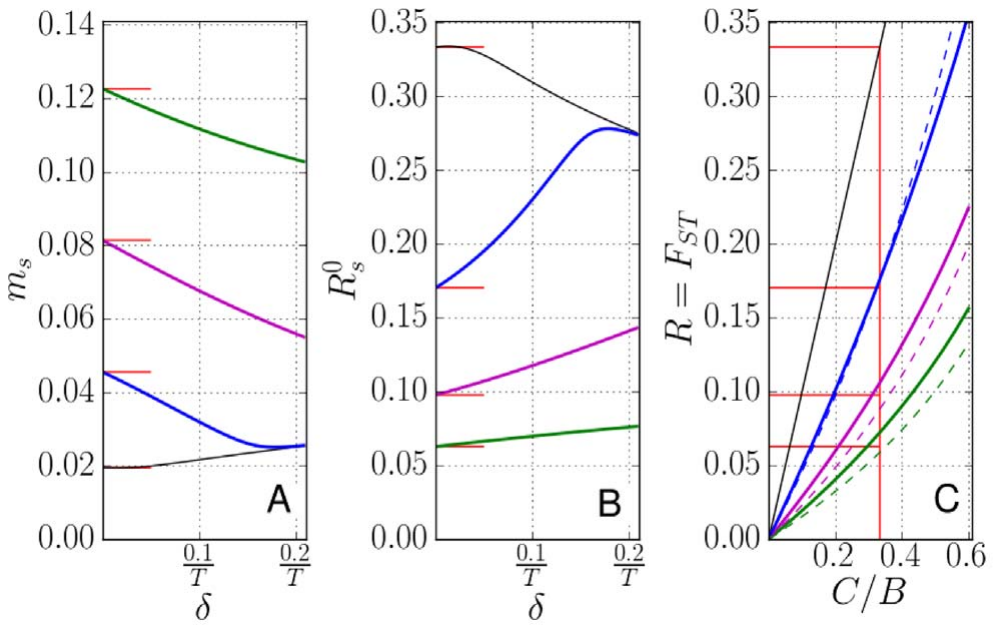
Iterated public goods game (IPG). A public goods game (PG) is repeated an average of 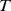 times in a life-cycle. In each round each individual can cooperate at a cost 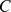 to herself, producing a benefit 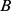 shared by the other members of the group. Types N never cooperate, while types A cooperate in the first round and later cooperate only if at least a fraction 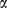 of group members cooperated in the previous round. In all panels 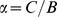 (types A are strongly altruistic) and curves correspond to 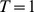 (black, this case is identical to PG), 10 (blue), 100 (magenta), 1000 (green) (bottom to top in Panel A, top to bottom in Panels B and C). **Panel A:** 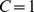, 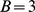, 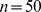. Curves give the critical migration rate 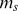 below which types A proliferate when rare, and that solves 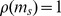, or equivalently 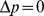 in (1). (The subscript ‘s’ stands for ‘survival’.) The dependence of 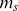 on the strength of selection 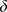 indicates the relevance of studying both weak and strong selection. Short horizontal red lines indicate value of 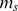 under weak selection, obtained from setting 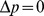 in (2) (note the excellent agreement). **Panel B:** Again, 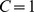, 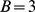, 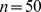. Curves give the critical relatedness 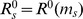 above which types A proliferate. Here 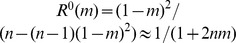 is the relatedness obtained from neutral genetic markers. Short horizontal red lines are again from 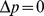 in (2). **Panel C:** Limit of large 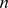 under weak selection. Critical values of relatedness 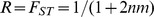, as function of 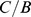. Solid lines provide the solution to the equation 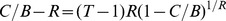 derived from setting 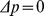 in (3). Dashed lines give its approximation (5). Red vertical line corresponds to 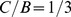, while horizontal red lines are at the same level of those from Panel B. Their intersections illustrate the fact that both the solid and dashed lines in Panel C are good approximations to weak-selection values of critical relatedness, 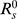, when 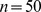.

Figure 2 displays a detailed analysis of some instances of the IPG, giving conditions for allele A to spread when rare (In this figure, 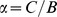, so that, in particular, types A are strongly altruistic). For many species that live and interact in groups for many years, several vital activities, including collective hunting and food sharing, can repeat themselves hundreds or thousands of times in a life-cycle, giving plausibility to the values of 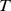 in Figure 2. The assumption that individuals discontinue behavior after a single unsuccessful participation is a simplification. When this is not a realistic assumption, we can, however, interpret the parameter 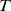 as the ratio between the typical number of repetitions of the activity and the typical number of unsuccessful attempts before cooperation is discontinued by a type A.

Panel C, in which selection is weak and groups are large, shows two important contrasting results. When 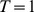, and the IPG is identical to the PG, allele A can only invade under Hamilton’s condition 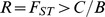. But as 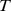 increases, the level of relatedness needed for invasion drops substantially, so that for modest values of 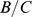, allele A can invade under 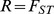 significantly lower than 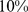, compatible with levels observed in several species, including humans [28] (Table 8.3), [30], [31] (Tables 6.4 and 6.5), [32], [33] (Table 4.9). The corresponding number of migrants per group per generation, 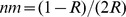, can be of the order of 10. Further examples showing the spread of altruism and cooperation under high levels of gene flow and low levels of relatedness are provided in Figure S5, S6, S7 and S8, S20 and S21 in File S1.

The 2-player iterated prisoner dilemma has been analysed in detail in the literature, as reviewed, for instance in Chapter 4 of [37]. In this setting, when types N are defectors and types A play tit-for-tat, types A will not be able to invade when rare, if assortment is random (because then they are typically paired with defectors and lose in fitness to those by cooperating in the first iteration). A very modest level of relatedness is nevertheless sufficient to allow tit-for-tat to proliferate when rare, as computed in Section 4.1.2 of [37]. Our computations here provide similar results for the 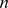-player iterated public goods game and correct the computations in Section 4.5.1 of that text, which had indicated that types A would require very high levels of relatedness to invade. In File S1 (Section S8, next to last subsection), we explain in detail what assumption in [36], reproduced in [37], led to that incorrect conclusion.

It is important to also emphasize a relevant difference between tit-for-tat in 2-player iterated prisoner’s dilemmas and types A with 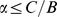 in 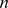-player public goods games (as in Figure 2). The former can proliferate under random assortment provided that initially they are not very rare. This is so because, when common, tit-for-tat is not altruistic; cooperation in each iteration assures continuation of cooperation, and in the long run benefits the cooperator. In contrast, the latter is strongly altruistic in the sense that 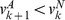 and therefore never proliferates under random assortment ([53], reviewed in [38]). In other words, while 2-player reciprocity is sometimes not altruistic, but rather cooperative, the behavior of types A that we are studying in Figure 2, is genuinely altruistic rather than simply cooperative.

### Condition for Invasion Under Strong Selection

To analyze the 2lFW, denote by 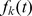, 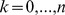 the fraction of groups in generation 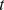 that have exactly 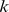 types A. Denote by 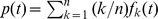 the frequency of types A in the population. The state of the population in generation 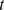 is described by the vector 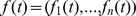, since 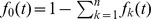. We will suppose that 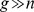, so that, by the law of large numbers, 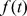 evolves as a deterministic (non-linear) dynamical system in dimension 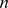. Here we will study its linearization close to the fixed point 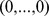, with no types A. This means that we are restricting ourselves to the case in which 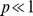, and studying the conditions for allele A to invade the population when rare. With the notation introduced in Fig. 1, we have then 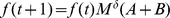, where
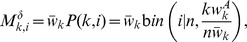


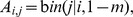


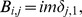
where 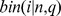 represents the binomial distribution with 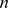 trials with probability of success 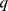 and we use the standard Kronecker notation 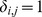 if 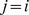 and 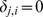 if 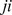. Matrix 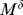 represents the production of groups in the new generation, through the two-level competition, prior to migration. Matrix 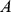 represents the effect of types A migrating out of groups, and matrix 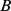 represents the effect of these migrant types A joining groups that previous to migration had no types A. (Explanation for 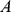 and 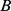: When 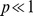, the migrant types A are a small fraction of the migrant population, and therefore each one is likely to settle in a different group that had no types A before migration. A group that had 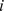 types A prior to migration will therefore have 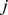 types A after migration with the probability given by 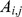 above. To understand the form of 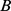 now, note that such a group that had 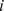 types A prior to migration will contribute an average of 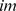 migrant types A, who will therefore produce that same number of groups with exactly one type 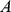 each.)

A standard application of the Perron-Frobenius Theorem (See Section 2 of File S1) implies that when 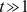, we have, in good approximation, 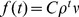, where 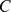 is a constant that depends on 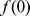, 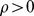 is the leading eigenvalue of 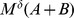 and 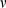 is its corresponding left-eigenvector normalized as a probability vector. This means that, regardless of the initial distribution 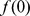, with 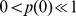, demographics and natural selection drive 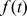 towards multiples of 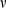, in what can be seen as self-organization of copies of A in the optimal stable way for them to spread. Once this has happened, 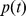 grows at rate 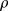. Consequently, allele A will proliferate, when rare, if the *viability condition* 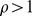 holds, and it will vanish if 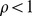 (see Figure 2 and Sections S1 and S2 in File S1 for applications and further explanations, see also Figure S2 to S9 in File S1 for illustrations). When 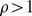, even if allele A is initially absent, a small rate of mutation will introduce it, allowing it to then invade the population. In the terminology of evolutionary game theory (see, e.g., [28](Chapter 7)), phenotype N is an evolutionary stable strategy (ESS) when 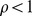 and N is not an ESS when 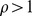.

The viability condition 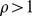 has a gene-centric (kin-selection) interpretation in terms of average (neighbor modulated) fitnesses. For this purpose, define 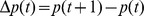. Then it is well known that 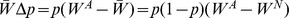, where 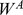 and 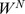 are the average fitnesses of types A and N, and 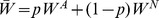 is the average fitness of all individuals. If we choose a random type A, it will have probability proportional to 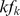 of being in a group with exactly 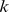 types A (Bayesian sampling bias, reviewed in Section S3 of File S1). Therefore 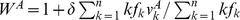. When 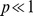, if we choose a random individual, it is likely to be in a group with no types A. Therefore, in good approximation, 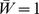. Since 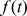 is driven towards multiples of 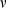, we obtain.
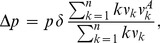
(1)provided 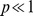 and 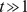 (the error term is of order 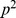
+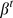, with 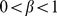). The viability condition 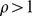 can also be stated as 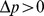, in (1). It is important to observe that 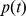 does not need to be monotone, and that 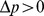 is the proper condition for invasion only when, as in (1), one is considering the stationary regime, 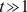. (See Figures S10, S11, S12 and S13 in File S1 for illustrations of the onset of this stationary regime.)

### Weak Selection

If selection is weak, i.e., 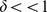, migration acts much faster than selection, providing a separation of time scales [3], [57], [63]–[65]. This allows us to replace 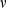 in (1) with 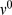, obtained by assuming 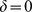 within error of order 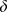 (see also Figure S15 and S16 in File S1). Defining now 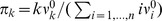, allows us then to rewrite the neighbor modulated fitness relation (1) in the form
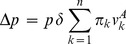
(2)(within error term of order 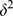). Algebraic simplifications (presented in Section S5 of File S1) transform the eigenvalue equation for 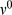 into the following equations for 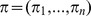:



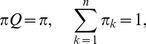
with the 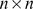 matrix 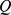 given by



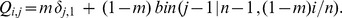
Matrix 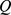 is a Markov transition matrix (i.e., 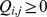 and 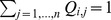) and 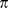 is its invariant probability distribution (see Section S5 in File S1 and Figure S17 in File S1 for details). They have natural interpretations in terms of identity by descent (IBD) under neutral genetic drift, as we explain next when we provide a second, independent, derivation of (2). This derivation is gene-centered, and is more intuitive and simpler than the derivations in the previous section. But it relies heavily on the assumption that selection is weak.

Two individuals are said to be IBD if following their lineages back in time, they coalesce before a migration event affects either one (see Figure S14 in File S1 for an illustration of the concept). The separation of time scales implies that when selection acts, the demographic distribution is well approximated by that obtained in equilibrium with 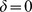. This means that in good approximation 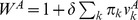, where 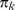 is the 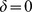 equilibrium probability that in the group of a randomly chosen focal type A there are exactly 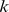 types A (focal included). But because we are supposing that types A are rare, the only individuals that are type A in this group are those that are IBD to the focal, so that 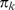 is also the probability that exactly 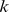 individuals in this group are IBD to the focal. As in the derivation of (1), since types A are rare, we have 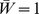 and hence 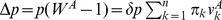, which is (2).

Now, the probability 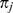 that the focal is IBD to exactly 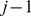 other members of her group is 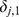 if the focal is a migrant (probability 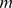), while if she is not a migrant (probability 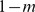), then we have to consider how many individuals in her mother’s group were IBD to her mother. If, counting her mother, that number was 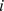 (probability 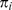, assuming demographic equilibrium) then the probability that the focal is IBD to exactly 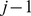 other members of her group is equal to the probability that of the 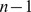 other members of her group, exactly 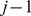 are non-migrants who chose for mother one of the 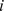 candidates (among 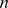 possibilities) that were IBD to the focal’s mother (probability 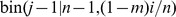). Combining these pieces, we have
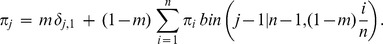



This is exactly the same as the set of equations 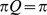.

The IBD distribution 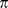 contains all the relevant information about genetic relatedness in the groups, including and exceeding that given by the average relatedness between group members, 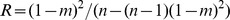 (see Figure S18 in File S1), obtained from lineages, regression coefficients, or Wright’s 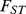 statistics. (See Sections S3 and S5 in File S1 for a review on these alternative, equivalent, definitions of relatedness, and its computation.) Specifically, we can define 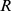 as the probability that a second member chosen from the focal’s group is IBD to the focal and then obtain (from linearity of expected values) that 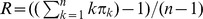 is a linear function of 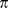
’s first moment. When 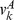 is a non-linear function of 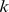, more information contained in 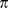, including its higher moments, is needed to decide whether 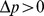 in (2). (In [66] the second moment of 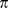 was used to obtain conditions for invasion by rare greenbeards in an island population structure.) It is important to also stress that (2) can be easily used for applications in which even the knowledge of all the moments of 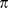 (see [63]) would be cumbersome to apply, as for instance in the computation of the short horizontal red lines in Figure 2, Panels A and B.

### Large Groups Under Weak Selection

The stationarity condition 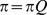 allows for a recursive computation of all the moments of 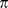 (see Section S5 in File S1). These moments can then be used to show the powerful result that if 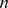 is large and 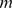 is small, then 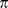, when properly rescaled, is close to a beta distribution, with mean 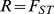 (see Section S6 and Figure S19 in File S1). In this case, if in addition to the assumption of weak selection, also 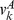 is well approximated by 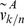, for some piecewise continuous function 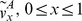, then (2) takes the easy to apply form
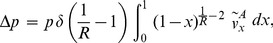
(3)where 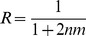. Equations (1) and (3) play complementary roles in the analysis of 2lFW. Both provide the condition for invasion by allele A; (1) holds in full generality, while (3) requires special assumptions (small 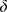, large 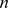), but is computationally much simpler and provides a great deal of intuition, as we discuss next.

Equation (3) should be contrasted with what [36] predicted by supposing that the number of individuals in a group that are IBD to a focal individual would be well approximated by a binomial with 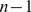 attempts and probability 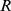 of success. That would lead to a normal distribution, narrowly concentrated close to its mean 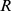, in place of the beta distribution above. Our result reveals a strong dependency structure among lineages, producing the beta distribution, with a standard deviation comparable to its mean, and a tail that decays slowly compared to a Gaussian distribution. As a consequence, fitness functions that are large only when the fraction of types A in a group is above a threshold value, as in the IPG, will allow for proliferation of types A under levels of relatedness substantially lower than that predicted under the assumption in [36] (for another example see also Section S7 and in File S1 and Figure S20 in File S1). We will refer to the fact that the fraction of group members that are IBD to a focal individual has a non-vanishing standard deviation, even when selection is weak and groups are large, as *persistence of variability*. This phenomenon poses a severe limitation to the applicability of covariance-regression methods in which regression of fitness on genotype is replaced with derivatives, as in [19], [16](Box 6), [7](condition (6.7)). Both the assumptions in [36], or in [19] applied to the IPG would have implied incorrectly that when selection is weak and groups are large, types A could only invade the population when rare if 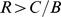 (these computations are presented in Sections S8 an S9 in File S1). In a companion paper [37] we show that methodologies in which one expresses the fitness of a focal individual in terms of partial derivatives with respect to the focal individual’s phenotype and the phenotype of the individuals with whom the focal interacts, as in [3], [7], [16], [19], [51], [57], require 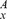 to be a linear function of 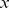.

For the PG, (2) and (3) clearly reduce to the well known 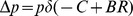. The same is also true for the more general (1), as was shown in [22], where in case of strong selection the relatedness 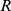 depends on the payoff functions. (The derivation in [22] was based on the Price equation. An alternative derivation is provided in Section 4 of File S1.) In contrast, if we are under the conditions of (3) with 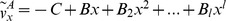, then
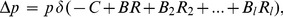
(4)where 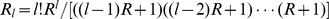 is the 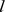-th moment of the beta distribution.

For the IPG, 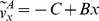, if 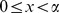, and 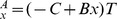, if 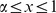. The viability condition derived from (3) can be analyzed in detail, by simple, but long, computations, presented in Section S8 in File S1 and illustrated by Figure S21–S26 in File S1. In the case 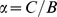, the viability condition reads 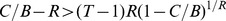. When 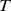 is large, this yields the following approximation for the critical relatedness 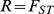:
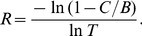
(5)


If also 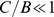, then
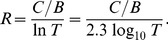
(6)


The simplicity and transparency of (5) and (6) illustrate the power of (3), and Fig. 2 shows how well they compare to the more general, but less transparent (1). Note also how (5) and (6) provide a direct grasp on the effect of the number of repetitions in the game, and a nice comparison between the PG and the IPG. Both Figure 2 and (6) show that alleles that promote contingent cooperative behavior, which is discontinued when participation is low, can spread under levels of genetic relatedness (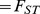) more than 5 times smaller than 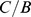. This mechanism should, therefore, be seriously investigated as a possible route for the proliferation of altruistic/cooperative behavior.

### Conclusions

1. Natural selection in group structured populations is best analyzed by a combination of group-centric and gene-centric perspectives and methods. Both shed light, carry intuition and provide computational power, in different ways. For instance, in this paper the analysis of invasion under strong selection focused on groups, while that under weak selection focused on genes. Our computations indicate the advantage of a pluralistic group-centric/gene-centric approach over views that regard one of these two approaches as being redundant, less informative, or counterproductive. Contrast, on one hand, with, e.g., [14], which to us seems excessively negative about the contributions of kin selection ideas. And contrast, on the other hand, with, e.g., Section 6.5 of [17], or the subsection on group selection in Chapter 15 of [29], which to us seem excessively negative about the contributions of group selection ideas).
2. Rigorous mathematical analysis of models is sometimes possible even when fitness functions are non-linear, selection is possibly strong, and the migration rate is arbitrary. And it is needed for assessing the validity of non-rigorous approaches, showing for instance that regression coefficients may be poorely approximated by partial derivatives, even when selection is weak. (Contrast with, e.g., [19](from (1) to (2)), [51]((3.4), (3.5)), [16](Box 6), [7](condition (6.7)).)
3. Hamilton’s condition for the spread of altruism, 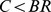, should be complemented with more general rules (like those obtained by setting 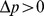 in (2), (3), or (4)) that extend it to non-linear fitness functions. In Section 9 of File S1, we compare these extensions of Hamilton’s condition to other extensions, based on covariance-regression formulas, like display (5) in [16]. We observe there that for the fundamental purpose of computing critical levels of relatedness needed for invasion, in term of parameters of the models, like in (5) and (6) above, covariance-regression formulas are incomplete in that they must be complemented with computations of the appropriate distribution of alleles in groups. And once these distributions are computed, our methods are much more direct and simpler.
4. Contingent forms of group altruism that are discontinued when participation is low can proliferate under biologically realistic conditions even in large groups. Their role in the spread of altruism should be empirically investigated. (Contrast with [36].)
5. Natural selection can promote traits that (in net terms over a full life-cycle) are costly to the actors and beneficial to the other members of their group, under demographic conditions that are not stringent. This can happen in large groups and with realistically high levels of gene flow, through population viscosity, without the need for kin recognition or greenbeard effects. Excessive focus on one-shot linear public goods games in the literature has obscured this fact. (Contrast with, e.g., [13], [17], [20]–[29].)

## Supporting Information

File S1
**Supporting Information. Figure S1,** Payoff profiles. Payoffs 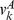 for types A are represented by black squares, while red circles depict payoffs 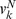 for types N. From left: Public goods game (PG, Example 1) for *n* = 20, *C* = 1 and *B* = 5. Iterated public goods game (IPG, Example 2) for *n* = 20, *C* = 1, *B* = 5, *a* = 4 and *T* = 2. Threshold model (THR, Example 3) for *n* = 20 *C* = 1, *θ* = 4 and *A* = *A*′ = 10. **Figure S2,** Perron-Frobenius eigenvalues *ρ* as a function of *m* for *δ* = 0.1, 0.2 and 0.4. From top to bottom: Public goods game (PG, Example 1) with *n* = 20, *C* = 1, *B* = 5. Iterated public goods (IPG, Example 2) with *n* = 20, *C* = 1, *B* = 5, *a* = 8 and *T* = 10. Threshold model (THR, Example 3) with *n* = 20, *C* = 1, *θ* = 4, *A* = *A*′ = 10. Critical migration values *m_s_* are obtained by solving *ρ*(*m_s_*) = 1. **Figure S3,** Public goods game (Example 1): Panel A represents critical values *m_s_* as a function of the strength of selection *δ*. Curves correspond to the case *C* = 1, *B* = 2 and *n* = 10 (top, black dotted line), *n* = 20 (middle, blue dashed line) and *n* = 50 (bottom, magenta full line). Short horizontal red lines indicate critical values at the weak selection limit obtained from (2) in the paper. The inset shows the same curves within the full range of possible values for *m_s_*, illustrating the well known fact that for this model, only under exceptional conditions can the allele A invade. Panel B depicts the same conditions except for *B* = 5. **Figure S4,** Public goods game (Example 1): Critical relatedness 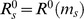 above which types A proliferate, as a function of the strength of selection *δ*. (*R* ^0^(*m*) = (1– *m*)^2^/(*n* – (*n* –1)(1– *m*)^2^) ≈ 1/(1+2*nm*) is the relatedness obtained from neutral genetic markers; see Sections S5 and S7). Panels correspond to the same parameter values as in Figure S3: *C* = 1, *B* = 2 and *n* = 10 (bottom, black dotted line), *n* = 20 (middle, blue dashed line) and *n* = 50 (top, magenta full line). Panel B depicts the same conditions except for *B* = 5. Short horizontal red lines indicate critical values at the weak selection limit obtained from Hamilton’s rule 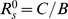, or, equivalently, from (2) in the paper. Note the appreciable effect of the strength of selection. **Figure S5,** Iterated public goods game (Example 2): Critical values *m_s_* as a function of the strength of selection *δ*. Panel A depicts the case *n* = 20, *C* = 1, *B* = 5, *a* = 4 with, respectively from bottom to top, *T* = 1 (dotted black line), *T* = 10 (dashed blue line), *T* = 100 (dot-dashed magenta) and *T* = 500 (green full line). Panel B depicts the same conditions except for *a* = 8. Short horizontal red lines indicate critical values at the weak selection limit obtained from (2) in the paper. Each curve has *T* fixed, but to compare different values of *T*, the product *δT* is a natural measure of strength of selection, and is used in the horizontal axis. **Figure S6,** Iterated public goods game (Example 2): Critical relatedness 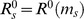 above which types A proliferate, as a function of the strength of selection *δ*. (*R* ^0^(*m*) = (1– *m*)^2^/(*n* – (*n* –1)(1– *m*)^2^) is the relatedness obtained from neutral genetic markers; see Sections S5 and S7). Panels correspond to the same parameter values as in Figure S5: Panel A depicts the case *n* = 20, *C* = 1, *B* = 5, *a* = 4 with, respectively from top to bottom, *T* = 1 (dotted black line), *T* = 10 (dashed blue line), *T* = 100 (dot-dashed magenta) and *T* = 500 (green full line). Panel B depicts the same conditions except for *a* = 8. As in Figure S5, each curve has *T* fixed, but to compare different values of *T*, the product *δT* is a natural measure of strength of selection, and is used in the horizontal axis. Short horizontal red lines indicate critical values at the weak selection limit obtained from (2) in the paper. These values are: Panel A: 0.2000, 0.0865, 0.0402, 0.0243. Panel B: 0.2000, 0.1099, 0.0638, 0.0452. Note the very low values of critical relatedness in Panel A. **Figure S7,** Threshold model (Example 3): Critical values *m_s_* as a function of the strength of selection *δ*. Panel A depicts the case *n* = 20, *C* = 1, *θ* = 4, *A*′ = 2*A*, with, respectively from bottom to top, *A* = 5 (dotted black line), *A* = 10 (dashed blue line), *A* = 50 (dot-dashed magenta) and *A* = 100 (green full line). Panel B depicts the same conditions except for *θ* = 8. Short horizontal red lines indicate critical values at the weak selection limit obtained from (2) in the paper. Each curve has *A* fixed, but to compare different values of *A*, the product *δA* is a natural measure of strength of selection, and is used in the horizontal axis. **Figure S8,** Threshold model (Example 3): Critical relatedness 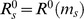 above which types A proliferate, as a function of the strength of selection *δ*. (*R* ^0^(*m*) = (1– *m*)^2^/(*n* – (*n* –1)(1– *m*)^2^) is the relatedness obtained from neutral genetic markers; see Sections S5 and S7). Panels correspond to the same parameter values as in Figure S7: Panel A depicts the case *n* = 20, *C* = 1, *θ* = 4, *A*′ = 2*A*, with, respectively from top to bottom, *A* = 5 (dotted black line), *A* = 10 (dashed blue line), *A* = 50 (dot-dashed magenta) and *A* = 100 (green full line). Panel B depicts the same conditions except for *θ* = 8. As in Figure S7, each curve has *A* fixed, but to compare different values of *A*, the product *δA* is a natural measure of strength of selection, and is used in the horizontal axis. Short horizontal red lines indicate critical values at the weak selection limit obtained from (2) in the paper. These values are: Panel A: 0.012, 0.017, 0.044, 0.071. Panel B: 0.061, 0.075, 0.137, 0.194. Note the extremely low values of critical relatedness in Panel A. The large values of *A* can result from contingent cooperation, based on feedback, as for the IPG. For instance, suppose that a certain activity repeats itself *T* times over a life-cycle. Suppose also that in each repetition the payoff is well described by the threshold model. If types A discontinue the participation when their payoff in the previous round was negative (as in the IPG discussed in Figure 2 in the paper), then the resulting payoff over the *T* iterations is also given by a threshold model, with the same value of *C*, but *A* replaced by (*A* – *C*)*T*+*C*, and *A*′ replaced with *A*′*T*. This gives plausibility to values of *A* and *A*′ as large as those in this figure, since *T* can be in the hundreds, or thousands (see discussion on the IPG in the paper). **Figure S9,** Public goods game (Example 1): Perron-Frobenius eigenvectors *ν* = (*ν*_1_, …, *ν_n_*) represented in each box as a histogram, as a function of the strength of selection *δ* (rows) and of the migration rate parameter *m* (columns). Critical migration rates *m_s_* are annotated in each row. Perron-Frobenius eigenvalues *ρ* are also provided for each box. In this picture we have *C* = 1, *B* = 2 and *n* = 20. **Figure S10,** Self-organization of copies of A. In these pictures we have PG with *n* = 2, *C* = 1, *B* = 3, *δ* = 0.3, resulting in *m_s_* = 0.2889. Pictures show evolution of *f*(*t*) = (*f*_1_(*t*), *f*_2_(*t*)), started from several different initial distributions *f*(0). Circles over the lines mark *f*(*t*), with *t* = 0, 1, …, 500 obtained by iterations of the map *f*(*t* +1) = *f*(*t*)*M*(*A*+*B*). The direction spanned by the eigenvector *ν* is represented as a dotted green line. Left side (black): cases with *ρ* <1, the allele A is eliminated; right side (red): cases with *ρ* >1, the allele A spreads. In the top row, *m* is far from *m_s_*: (A1) *m* = 0.3389, *ρ* = 0.9340, *ν* = (0.8506, 0.1494); (B1) *m* = 0.2389, *ρ* = 1.078, *ν* = (0.7342, 0.2658). In the bottom row, *m* is close to *m_s_*: (A2) *m* = 0.2890, *ρ* = 0.999856, *ν* = (0.7342, 0.2658); (B2) *m* = 0.2888, *ρ* = 1.000014, *ν* = (0.7999, 0.2001). Note that in all cases *f*(*t*) reaches in a few generations a steady state, in which it shrinks (*ρ* <1), or grows (*ρ* >1), as a multiple of *ν*. When *m* approaches *m_s_*, the eigenvalue *ρ* becomes close to 1, the stationary movement along the direction given by *ν* slows down and the trajectories towards this direction straighten themselves, but are not slowed down. **Figure S11,** Self-organization of copies of A. In this picture we have IPG with *n* = 10, *C* = 1, *B* = 3, *T* = 100, *a* = 2, *δ* = 0.01, and *m* = 0.153, slightly smaller than *m_s_* = 0.163. Top part shows evolution of *p*(*t*), and bottom part shows corresponding evolution of *f*(*t*) = (*f*_1_(*t*), …, *f*_10_(*t*)), displayed as normalized histograms. Two initial conditions are compared: (Red) *f*(0) = 10^–2^(1, 0, …, 0), so that *p*(0) = 10^–3^. (Black) *f*(0) = 10^–5^(0, …, 0, 1), so that *p*(0) = 10^–5^. Note that from generation to generation the distribution of copies of A adjusts itself to the same stationary distribution, “losing memory of the initial distribution”. **Figure S12,** Self-organization of copies of A. This picture corresponds to the same model and situation described in Figure S11, but with a different time-frame, including later times. Note that eventually the two curves of *p*(*t*) become parallel straight lines, illustrating the exponential growth of *p*(*t*) at rate *ρ* independently of the initial condition. This picture also illustrates two other important points: 1) The possible non-monotonicity of *p*(*t*). 2) The fact that the asymptotic rate of growth may be smaller than the initial rate of growth. Indeed, computations of Δ*p* only indicate the long term prospects for the allele A, when done under stationary conditions, as in (1). The initial distribution of copies of A in the red line produces neighbor modulated fitness for A below that of allele N, so that Δ*p*(0) <0. In contrast, the initial distribution of copies of A in the black line produces neighbor modulated fitness for A well above that of allele N, so that not only Δ*p*(0) >0, but this growth happens at an unsustainably high rate. The distribution *ν*, towards which the copies of A self-organize is optimal for their stationary, stable, growth. This is so because (*ρ*, *ν*) is the leading eigenpair of the driving matrix *M*(*A*+*B*): *ν* is the vector *ν*′ that satisfies the eigenvalue (stationarity) equation *ν*′*M*(*A*+*B*) = *ρ*′*ν*′, with maximum *ρ*′. **Figure S13,** Self-organization of copies of A. This picture corresponds to the same model described in Figure S11, but now *m* = 0.173 is slightly larger than *m_s_* = 0.163. Note that again eventually the two curves of *p*(*t*) become parallel straight lines, illustrating in this case the exponential decrease of *p*(*t*) at a rate independent of the initial condition. Here again one can see that Δ*p*(0) is not indicative of the relevant long term evolution. The self-organized distribution *ν* is still optimal for the proliferation of the allele A in a stable, sustainable, fashion. But when *ρ* <1, as in this picture, this optimal stable distribution is still not good enough for A to spread, and instead, its copies are eliminated by natural selection. **Figure S14,** This diagram illustrates the concept of identity by descent (IBD) in the 2lFW. Two individuals *X* an *Y* in a given group in generation *t*, regardless of their type, are identical by descent (IBD) if their lineages, when followed back in time, coalesce before a migration event (indicated by a dashed arrow in the figure in the right panel). Considering a migration rate of *m*, migration typically takes place within a random number, of order 1/*m* of generations back. **Figure S15,** Perron-Frobenius eigenvectors *ν* = *ν^δ^* for selection strengths *δ* = 0.01 (left column), *δ* = 0.3 (middle column) and *δ* = 0.7 (right column). Migration rate is set to *m* = 0.1 and group sizes to *n* = 20. Each line represents a different model. The top row, labeled as PG depicts the Public Goods game (Example 1) with parameters *C* = 1 and *B* = 2. The Iterated Public Goods game (Example 2) with parameters *C* = 1, *B* = 4, *a* = 4 and *T* = 10 is shown in row at the middle, labeled as IPG. The bottom row shows Perron-Frobenius eigenvectors for the Threshold model (THR, Example 3) with *C* = 1, *A* = *A*′ = 5 and *θ* = 4. The leftmost column emphasizes that the weak selection limit 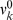 is independent of the model. In contrast, when selection is strong, *ν^δ^* depends on the model, as illustrated in the other columns. **Figure S16,** Distribution *π_k_* (bars) given by *π* = *πQ* and 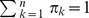, compared with 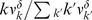. Here *ν^δ^* is the Perron-Frobenius eigenvector of *M*(*A*+*B*), with *δ* = 0.01, for the Threshold model (THR, Example 3) with parameters *n* = 20, *C* = 1, *A* = *A*′ = 5 and *θ* = 4 (red diamonds). The comparison is repeated for migration rates *m* = 0.01 (top panel) and *m* = 0.1 (bottom panel). **Figure S17,** This diagram illustrates why *K^D^* evolves as a Markov chain driven by *Q*. In this picture 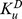 represents the number of individuals that are IBD to the focal individual 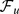, in generation *u* (red circle). Two scenarios are discernible. MC1 (left panel): the focal individual is a migrant. This happens with probability *m* and implies that 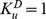. MC2 (right panel): the focal individual 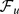 is not a migrant, and she is a child of 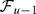. Each individual in the focal group in generation *u* chooses a mother from the group of 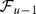 in the previous generation with uniform probability, as *δ* = 0. With probability 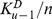 the chosen mother is IBD to 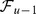 (orange circles) and, consequently, her children are also IBD to 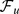, provided that they are not migrants. In this case, the number of individuals in generation *u* that are IBD to the focal is, therefore, 1 (for the focal individual herself) plus a number of individuals given by a binomial random variable with probability of success 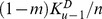 in *n* –1 trials. **Figure S18,** Relatedness *R* ^0^ as a function of migration rate *m*, under neutral drift, *δ* = 0, as given by (S11). From top to bottom, *n* = 20 (dot-dashed blue line), *n* = 50 (dashed green line) and *n* = 100 (full red line). **Figure S19,** Limit of large *n* and small *m* under weak selection. This figure compares tail probabilities for the distribution *π* (stairs) and for Beta distributions with parameters *α* = 1 and *β* = 2*mn*. Panel A shows the case *n* = 20 for, from top to bottom, *m* = 0.01 (red dotted line), *m* = 0.1 (blue dashed line) and *m* = 0.5 (black dot dashed line). Panel B depicts the same scenarios for the case *n* = 100. **Figure S20,** Limit of large *n* under weak selection for the threshold model (THR, Example 3). Panels represent critical migration rates (A and C) and critical relatedness (B and D) for the THR with *C* = 1, *A* = *A*′ = 10 as a function of 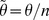. Top panels A and B depict the case *n* = 20. Bottom panels C and D depict the case *n* = 100. In each panel critical values obtained by the viability condition under weak selection derived from (A2) (black full lines) are compared with the approximation for large *n* given by (S25) (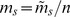, 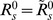, approx.1, dashed blue lines) and with the approximation (S26) (approx.2, dotted red lines). **Figure S21,** Limit of large *n* under weak selection for the Iterated public goods (IPG) game (Example 2). Panels represent critical migration rates (A and C) and critical relatedness (B and D) for the IPG with *C* = 1, *B* = 5 and *T* = 100 as a function of 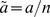. Top panels A and B depict the case *n* = 20. Bottom panels C and D depict the case *n* = 100. In each panel critical values obtained by the viability condition under weak selection derived from (2) (black full lines) are compared with the approximation for large *n* given by solving (S27) in *R* (approx., dashed blue lines). In panel B we have 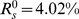 when 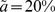, and in panel D we have 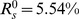 when 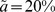. Types A are altruistic in the strong sense of (S31) when 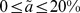. **Figure S22,** Limit of large *n* under weak selection for the Iterated public goods (IPG) game (Example 2): behavior of solutions for (S27) - Part 1. Top panel: *H*(*R*) corresponds to the l.h.s. of (S27) while *G*(*R*) depicts the r.h.s. of (S27). *H*(*R*) is strictly decreasing and it is positive for *R*<*C*/*B*. Derivatives of *G*(*R*) converge to 0 as *R* → 0. *H*(*R*) and *G*(*R*) are equal to each other at exactly one point 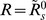 that is a decreasing function of *C*/*B*. Curves depicted correspond to the cases *C*/*B* = 0.5 (full black line), *C*/*B* = 0.2 (dashed red line) and *C*/*B* = 0.1 (dot-dashed blue line) with 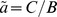 and *T* = 100. Bottom panel: 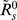 as a function of 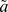 for *C*/*B* = 0.5 (top, full black line), *C*/*B* = 0.2 (middle, dashed red line) and *C*/*B* = 0.1 (bottom, dot-dashed blue line) and *T* = 100. 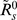 is continuous in the interval 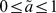, takes the value *C*/*B* on both end-points of this domain and has a minimum at 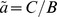. **Figure S23,** Limit of large *n* under weak selection for the Iterated public goods (IPG) game (Example 2): behavior of solutions for (S27) - Part 2. Top panel: *G*(*R*) and *H*(*R*) for *C*/*B* = 0.5, 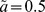 and *T* = 10 (leftmost, full black line), *T* = 10^3^ (dashed red line) and *T* = 10^5^ (dot-dashed blue line). 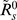 is a decreasing function of *T*. Bottom panel: in the limit *T* → ∞, if 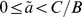 then 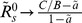 (full magenta line). If 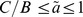 then 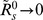 very slowly. **Figure S24,** Limit of large *n* under weak selection for the Iterated public goods (IPG) game (Example 2): behavior of solutions for (S27) - Part 3. *H*(*R*) (strictly decreasing straight line) and *G*(*R*) for *C*/*B* = 0.5 and *T* = 10 for 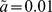, 0.1, 0.3, 0.4, 0.5 from right to left in Panel A and for 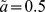, 0.6, 0.7, 0.9, 0.999 from left to right in Panel B. The graph of *G*(*R*) moves upwards for 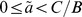 and downwards for 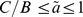. *G*(*R*) → (*T* –1)(*BR* – *C*) as 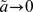 (dashed magenta line in Panel A). In Panel B it can be seen that *G*(*R*) → 0 as 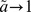. **Figure S25,** Limit of large *n* under weak selection for the Iterated public goods (IPG) game (Example 2): behavior of solutions for (S27) - Part 4. In all panels *C*/*B* = 0.5. Panel A depicts 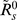 as a function of 1/log(*T*) for 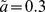 (full black line) and for 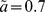 (dashed red line). For 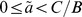 
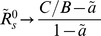
 (this value is approximately 0.286 for the case shown). If 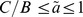 then 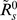 converges to 0 very slowly as *T* increases, more specifically 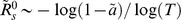 (dotted magenta line). Bottom panels show the behavior of *G*(*R*) as *T* increases. Panel B: case 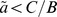 for, from right to left, *T* = 2, 10, 100, 500. Panel A: case 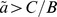 for *T* = 2, 10, 100, 500, from right to left. *G*(*R*) stays at zero for 
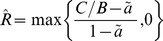
 and goes monotonically to infinity for 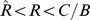. **Figure S26,** The solid lines provide the solution 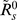 of *C*/*B* – *R* = (*T* –1)*R*(1– (*C*/*B*))^1/*R*^, as a function of *C*/*B*, for (top to bottom) *T* = 1 (black), 10 (blue), 100 (magenta), 1000 (green) and 10000 (cian). The corresponding dashed lines with same colors (no black one) provide the approximation (A5), *R* = –ln(1– (*C*/*B*))/ln *T*. This figure is an expanded version of Panel C of Figure 2 in the paper.(PDF)
