# Supplementary material for: Altruism Can Proliferate through Population Viscosity despite High Random Gene Flow
Source: PLoS One. 2013 Aug 19;8(8):e72043. doi: 10.1371/journal.pone.0072043 (PMC3747169; doi:10.1371/journal.pone.0072043)
Supplement: File S1 — Supporting Information. Figure S1, Payoff profiles. Payoffs for types A are represented by black squares, while red circles depict payoffs for types N. From left: Public goods game (PG, Example 1) for n = 20, C = 1 and B = 5. Iterated public goods game (IPG, Example 2) for n = 20, C = 1, B = 5, a = 4 and T = 2. Threshold model (THR, Example 3) for n = 20 C = 1, θ = 4 and A = A′ = 10. Figure S2, Perron-Frobenius eigenvalues ρ as a function of m for δ = 0.1, 0.2 and 0.4. From top to bottom: Public goods game (PG, Example 1) with n = 20, C = 1, B = 5. Iterated public goods (IPG, Example 2) with n = 20, C = 1, B = 5, a = 8 and T = 10. Threshold model (THR, Example 3) with n = 20, C = 1, θ = 4, A = A′ = 10. Critical migration values ms are obtained by solving ρ(ms) = 1. Figure S3, Public goods game (Example 1): Panel A represents critical values ms as a function of the strength of selection δ. Curves correspond to the case C = 1, B = 2 and n = 10 (top, black dotted line), n = 20 (middle, blue dashed line) and n = 50 (bottom, magenta full line). Short horizontal red lines indicate critical values at the weak selection limit obtained from (2) in the paper. The inset shows the same curves within the full range of possible values for ms, illustrating the well known fact that for this model, only under exceptional conditions can the allele A invade. Panel B depicts the same conditions except for B = 5. Figure S4, Public goods game (Example 1): Critical relatedness above which types A proliferate, as a function of the strength of selection δ. (R 0(m) = (1– m)2/(n – (n –1)(1– m)2) ≈ 1/(1+2nm) is the relatedness obtained from neutral genetic markers; see Sections S5 and S7). Panels correspond to the same parameter values as in Figure S3: C = 1, B = 2 and n = 10 (bottom, black dotted line), n = 20 (middle, blue dashed line) and n = 50 (top, magenta full line). Panel B depicts the same conditions except for B = 5. Short horizontal red lines indicate critical values at the weak sel [file pone.0072043.s001.pdf]

Supporting information for: Altruism can proliferate through population viscosity despite high random gene flow

Roberto H. Schonmann, Renato Vicente, Nestor Caticha

July 20, 2013

Emails: rhs@math.ucla.edu, rvicente@ime.usp.br, nestor@if.usp.br

In this supporting information, (A1), (A2), ... refer to displays (1), (2) ... in the paper

Contents

S1 Examples. Meaning of ρ and ν. Self organization **2**

S2 Use of Perron-Frobenius Theorem **17**

S3 Focal individual. Sampling bias. Relatedness. F_{ST}. IBD **17**

S4 Linear payoff function **21**

**S5 Limit of weak selection.
IBD distribution π as a needed extension of relatedness** **21**

**S6 Limit of large n and small m under weak selection.
IBD distribution is close to beta distribution** **29**

S7 Critical values **32**

**S8 Iterated public goods game.
Types A play multi-individual tit-for-tat** **35**

**S9 Time periods, mutations, viability conditions,
covariance-regression formulas** **47**

S1 Examples. Meaning of ρ and ν. Self organization

Example 1. (PG): *Public goods game:*

$$\begin{aligned} v_k^A &= -C + (k-1)B/(n-1), \\ v_k^N &= kB/(n-1), \end{aligned}$$

where, unless stated otherwise, we suppose that $0 < C < B$. This is, by far, the most widely studied model of cooperative/altruistic behavior. At a net cost C to itself, each type A provides a total benefit B shared uniformly among the other $n-1$ members of the group. This model can also be used when at a gross cost c to itself, each type A provides a total benefit b shared uniformly among the n members of the group, self included. By defining $C = c - b/n$ and $B = (n-1)b/n$, we obtain the payoff functions displayed above also in this case.

Example 2. (IPG): *Iterated public goods game. Altruists cooperate conditionally:*

$$\begin{aligned} v_k^A &= \begin{cases} -C + (k-1)B/(n-1), & \text{if } \ k \le a, \\ T\,(-C + (k-1)B/(n-1)), & \text{if } \ k > a, \end{cases} \\ v_k^N &= \begin{cases} kB/(n-1), & \text{if } \ k \le a, \\ T\,kB/(n-1), & \text{if } \ k > a, \end{cases} \end{aligned}$$

for constants $0 < C < B$, $T \ge 1$ and $a \in \{1, 2, ..., n-1\}$. Here we suppose that a public goods game is repeated a random number of times $\tau \ge 1$, with average $\mathbb{E}(\tau) = T$. Each time each member of the group can cooperate at a cost C to itself, resulting in a benefit $B/(n-1)$ to each one of the other members of its group. Defectors incur no costs and produce no benefits. We suppose that types A cooperate in the first round, and afterwards only cooperate if at least a other members of the group cooperated in the previous round. This is a generalization of the well known tit-for-tat strategy, which corresponds to the case $n = 2$, $a = 1$. We will refer to the strategy of the types A in this example then as "multi-individuals-tit-for-tat (with threshold a)". In the paper, we emphasized the case in which each type A cooperates in the first round, and in later rounds cooperates if and only if its payoff in the previous round was not negative. This corresponds to a taking the smallest integer value that is larger than or equal to $(n-1)C/B$ (since the PG has $v_k^A < 0$ if and only if $k < 1 + (n-1)C/B$). In this important case, we call the strategy of types A "payoff dependent contingent cooperation".

The IPG was studied independently in [3] and in [9], in the trait-group framework, which corresponds to the 2lFW with $m = 1$, i.e., random assortment in groups. Both papers identified stable equilibria with positive fractions of types A. But they also observed that altruists could not invade when rare, when $m = 1$.

The relevance of this model was emphasized in the paper, and it will be analysed in detail in Section S8.

Example 3. (THR): *Threshold model:*

$$v_k^A = \begin{cases} -C, & \text{if } k < \theta, \\ -C + A, & \text{if } k \geq \theta, \end{cases}$$
$$v_k^N = \begin{cases} 0, & \text{if } k < \theta, \\ A', & \text{if } k \geq \theta, \end{cases}$$

for positive constants C, A and A', and an integer $\theta \in \{1, 2, ..., n\}$. The idea here is simple: the allele A carries a cost, but allows its carriers to gain benefits if sufficiently many are in the group. Unless otherwise stated, we assume that $A > C$. Types N obtain benefits also when types A do, but we allowed for the possibility that these benefits are different from those of the types A. These payoff functions can be seen as simplifications of more realistic ones, in which payoffs to types A initially grow slowly with k, then steeply, and then quickly saturate. For instance, the model of coordinated punishment of [2] provides examples of this sort. In [1] a class of models that bridge between the PG and the THR was introduced and their relevance discussed. Similarly to the results of [3] and [9] for the IPG, it was shown in [1] that the THR can have polimorphic equilibria, with Types A and N coexisting, when assortment is random, i.e., in the trait-group framework, that corresponds to 2lFW with $m = 1$. In this case, as for the IPG types A can nevertheless never proliferate, when p is small (the equilibrium with no types A is an ESS). In [17], the case $n = 3$ of the threshold model (called there "stag hunt game") was discussed in connection to the conceptual issue of the role of Hamilton's rule.

The THR behaves in a very simple way under iteration. Suppose that the game is repeated T times over a life-cycle. And suppose that types A are "payoff dependent conditional cooperatores", meaning that a type A cooperates in the first round, and in later rounds cooperates if and only if its payoff in the previous round was not negative. The result is that types A cooperate exactly once if $k < \theta$, and cooperate T times if $k \geq \theta$. The total payoffs to types A and to types N over the life-cycle are then those of a THR with the same value of C, with A replaced by $T(A - C) + C$ and A' replaced by TA'.

Fig. **??** displays instances of the three models above. A more detailed discussion of these and other examples, can be found in [15], where several conceptual issues are also considered. A good source of motivated examples is [8]. Fig. S2 displays curves of ρ vs. m for instances of the three examples above. In each case, ρ is decreasing in m, and there is a single critical value m_s defined by $\rho(m_s) = 1$ (the subscript 's' stands for 'survival' of the allele A, when rare). The critical rate of gene flow that allows types A to proliferate when rare can also be identified by the critical relatedness $R_s^0 = R^0(m_s)$, where $R^0(m) = (1-m)^2/(n-(n-1)(1-m)^2) \approx 1/(1+2nm)$ (the approximation refers to the case in which m is small) is the relatedness obtained from neutral genetic markers (see Sections S5 and S7). Fig. S3, Fig. S5 and Fig. S7 display values of m_s as function of the strength of selection δ. In these pictures, the short horizontal red lines indicate the limits as $\delta \to 0$, obtained by using (A2). Note the good agreement between these values

obtained from (A2) and the asymptotic behavior of m_s with vanishing δ in the pictures. As in Panel A of Fig. 2 in the paper, the region above the curve defined by m_s represents the region where the allele A cannot invade the population, while in the region below this curve it can invade the population. Fig. S4, Fig. S6 and Fig. S8 display corresponding values of critical relatedness $R_s^0 = R^0(m_s)$ above which types A proliferate, as a function of the strength of selection δ. In Fig. S5 and Fig. S6, the case $T = 1$ corresponds to the PG, and comparison between the results for the various values of T illustrates again the strength of group selection under contingent cooperative behavior. In Fig. S7 and Fig. S8 a similar phenomenon is shown for the threshold model, under contingent cooperation. Fig. S9 shows how, even for the standard public goods game, with $B = 2C$, the distribution ν depends on δ and m. Fig. S10, Fig. S11, Fig. S12 and Fig. S13 illustrate features of the evolution $f(t+1) = f(t)M(A+B)$, that self-organizes the copies of the allele A. These pictures also illustrate the meanings of ρ and ν.

It is natural to wonder if there are simple conditions under which one can guarantee that (a) when $m = 1$ (random assortment) types A will not proliferate when rare, but (b) that they will proliferate when rare when the migration rate m is low. The following conditions, satisfied by the three examples above, imply, respectively, each one of these two facts (see Section S7).

(C1) $v_1^A < 0$, i.e., $w_1^A < 1 = w_0^N$, so that an isolated type A individual has lower fitness than the wild type N has in groups without altruists.

(C2) $v_n^A > 0$, i.e., $w_n^A > 1 = w_0^N$, so that type A individuals have greater fitness when in single-type groups than type N individuals have when in single-type groups.

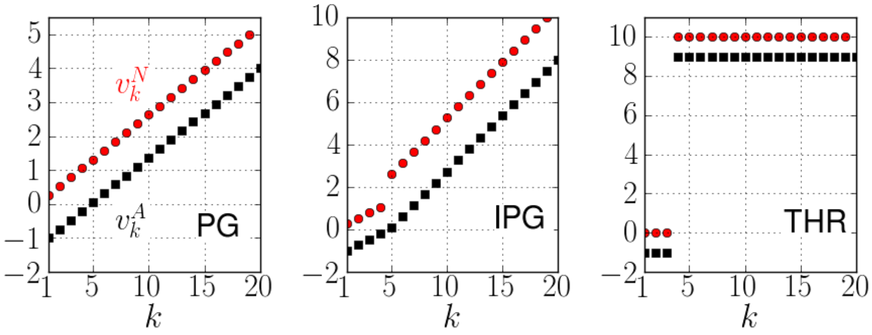

Figure S1: Payoff profiles. Payoffs v_k^A for types A are represented by black squares, while red circles depict payoffs v_k^N for types N. From left: Public goods game (PG, Example 1) for $n = 20$, $C = 1$ and $B = 5$. Iterated public goods game (IPG, Example 2) for $n = 20$, $C = 1$, $B = 5$, $a = 4$ and $T = 2$. Threshold model (THR, Example 3) for $n = 20$ $C = 1$, $\theta = 4$ and $A = A' = 10$.

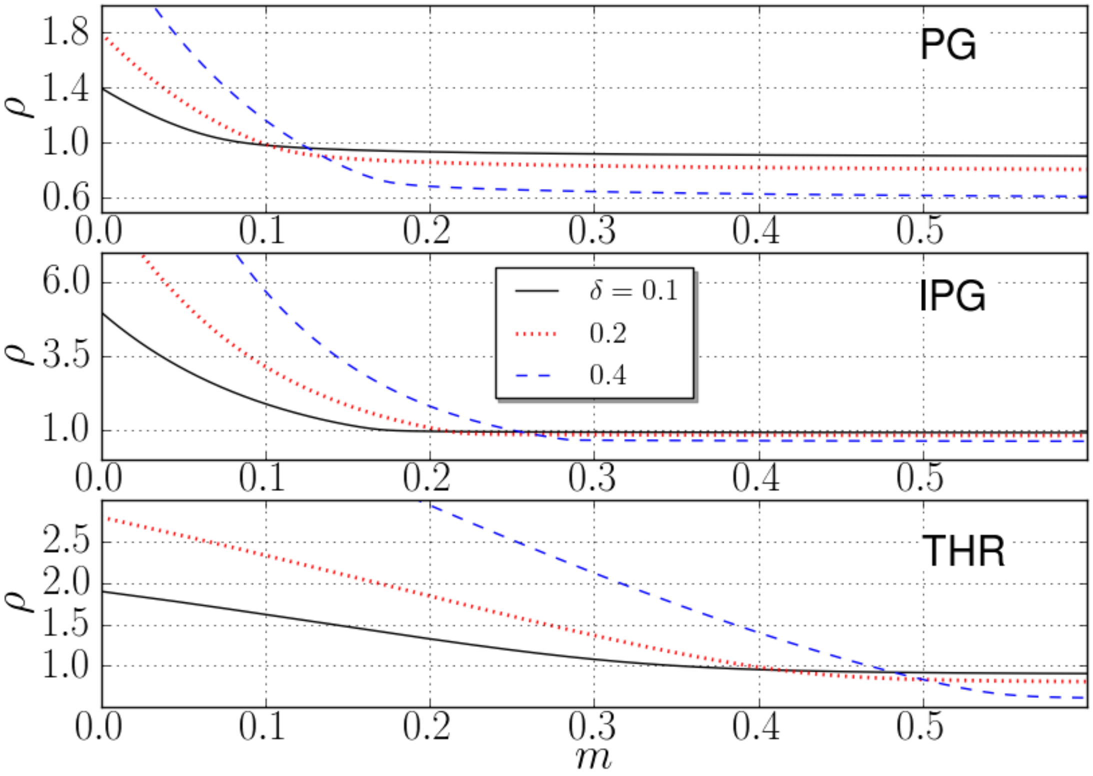

Figure S2: Perron-Frobenius eigenvalues ρ as a function of m for $\delta = 0.1$, 0.2 and 0.4. From top to bottom: Public goods game (PG, Example 1) with $n = 20$, $C = 1$, $B = 5$. Iterated public goods (IPG, Example 2) with $n = 20$, $C = 1$, $B = 5$, $a = 8$ and $T = 10$. Threshold model (THR, Example 3) with $n = 20$, $C = 1$, $\theta = 4$, $A = A' = 10$. Critical migration values m_s are obtained by solving $\rho(m_s) = 1$.

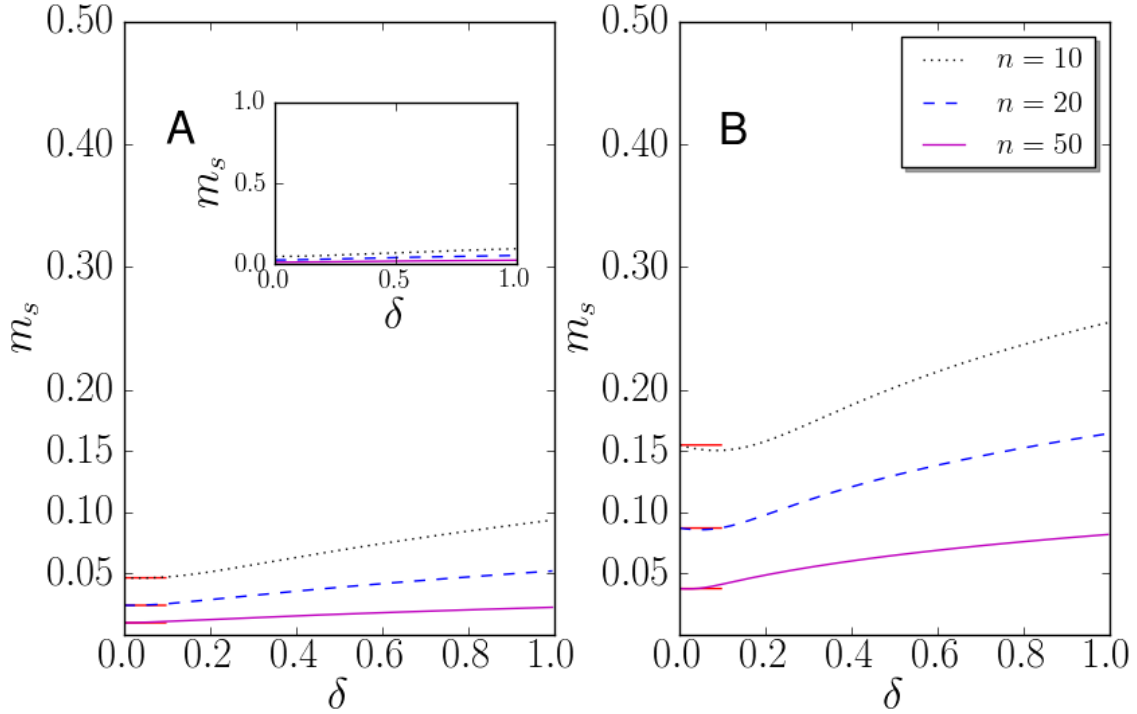

Figure S3: Public goods game (Example 1): Panel A represents critical values m_s as a function of the strength of selection δ. Curves correspond to the case $C = 1$, $B = 2$ and $n = 10$ (top, black dotted line), $n = 20$ (middle, blue dashed line) and $n = 50$ (bottom, magenta full line). Short horizontal red lines indicate critical values at the weak selection limit obtained from (A2) in the paper. The inset shows the same curves within the full range of possible values for m_s, illustrating the well known fact that for this model, only under exceptional conditions can the allele A invade. Panel B depicts the same conditions except for $B = 5$.

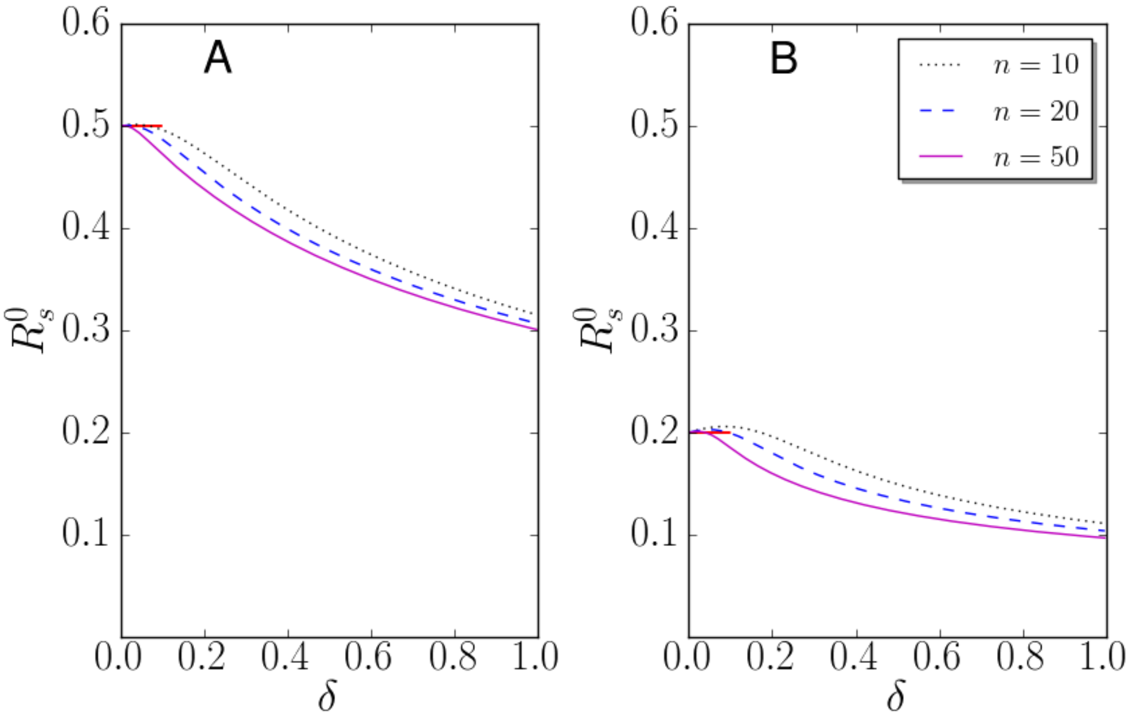

Figure S4: Public goods game (Example 1): Critical relatedness $R_s^0 = R^0(m_s)$ above which types A proliferate, as a function of the strength of selection δ. ($R^0(m) = (1-m)^2/(n-(n-1)(1-m)^2) \approx 1/(1+2nm)$ is the relatedness obtained from neutral genetic markers; see Sections S5 and S7). Panels correspond to the same parameter values as in Fig. S3: $C = 1$, $B = 2$ and $n = 10$ (bottom, black dotted line), $n = 20$ (middle, blue dashed line) and $n = 50$ (top, magenta full line). Panel B depicts the same conditions except for $B = 5$. Short horizontal red lines indicate critical values at the weak selection limit obtained from Hamilton's rule $R_s^0 = C/B$, or, equivalently, from (A2) in the paper. Note the appreciable effect of the strength of selection.

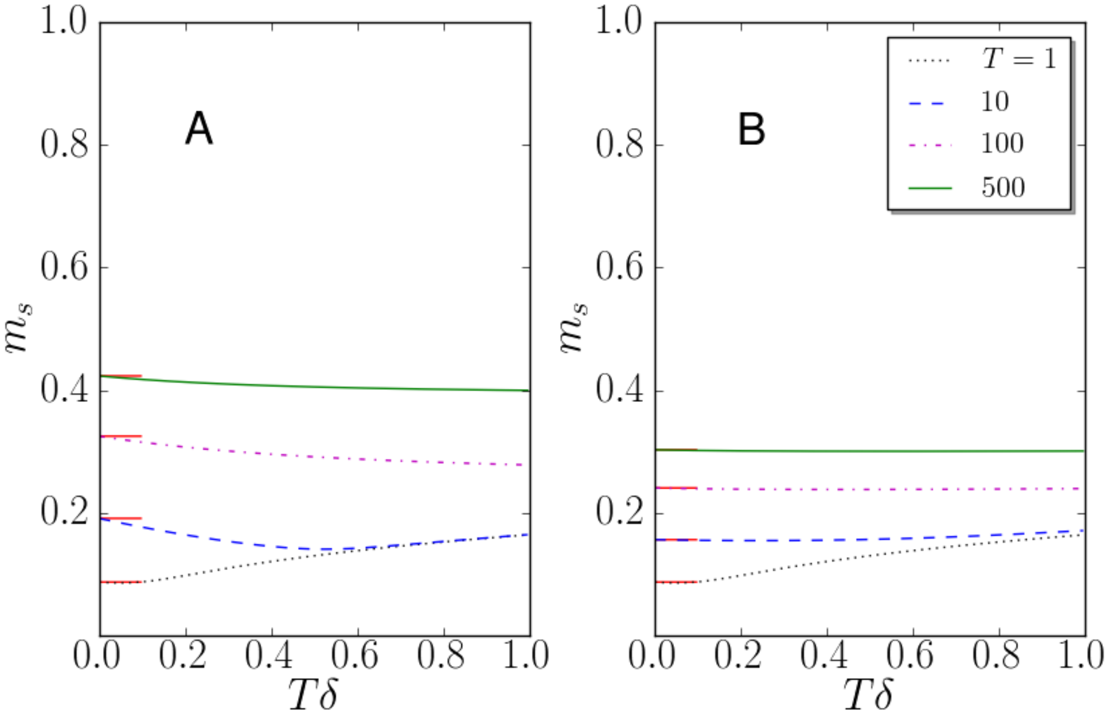

Figure S5: Iterated public goods game (Example 2): Critical values m_s as a function of the strength of selection δ. Panel A depicts the case $n = 20$, $C = 1$, $B = 5$, $a = 4$ with, respectively from bottom to top, $T = 1$ (dotted black line), $T = 10$ (dashed blue line), $T = 100$ (dot-dashed magenta) and $T = 500$ (green full line). Panel B depicts the same conditions except for $a = 8$. Short horizontal red lines indicate critical values at the weak selection limit obtained from (A2) in the paper. Each curve has T fixed, but to compare different values of T, the product δT is a natural measure of strength of selection, and is used in the horizontal axis.

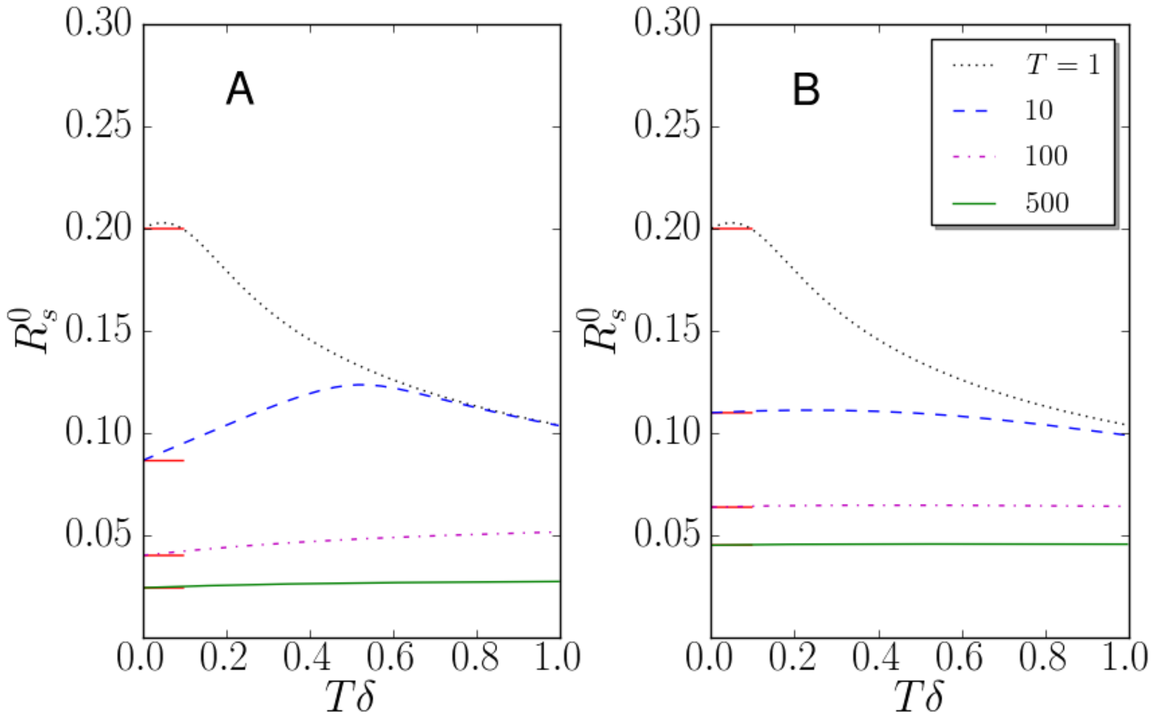

Figure S6: Iterated public goods game (Example 2): Critical relatedness $R_s^0 = R^0(m_s)$ above which types A proliferate, as a function of the strength of selection δ. ($R^0(m) = (1-m)^2/(n-(n-1)(1-m)^2)$ is the relatedness obtained from neutral genetic markers; see Sections S5 and S7). Panels correspond to the same parameter values as in Fig. S5: Panel A depicts the case $n = 20$, $C = 1$, $B = 5$, $a = 4$ with, respectively from top to bottom, $T = 1$ (dotted black line), $T = 10$ (dashed blue line), $T = 100$ (dot-dashed magenta) and $T = 500$ (green full line). Panel B depicts the same conditions except for $a = 8$. As in Fig. S5, each curve has T fixed, but to compare different values of T, the product δT is a natural measure of strength of selection, and is used in the horizontal axis. Short horizontal red lines indicate critical values at the weak selection limit obtained from (A2) in the paper. These values are: Panel A: 0.2000, 0.0865, 0.0402, 0.0243. Panel B: 0.2000, 0.1099, 0.0638, 0.0452. Note the very low values of critical relatedness in Panel A.

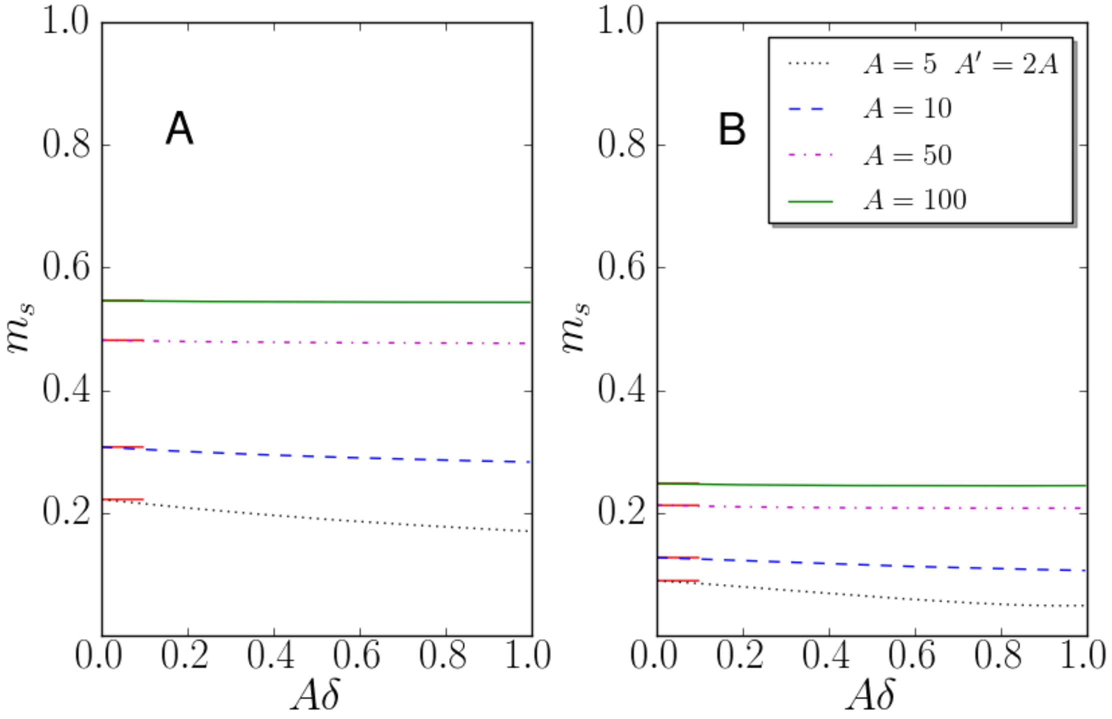

Figure S7: Threshold model (Example 3): Critical values m_s as a function of the strength of selection δ. Panel A depicts the case $n = 20$, $C = 1$, $\theta = 4$, $A' = 2A$, with, respectively from bottom to top, $A = 5$ (dotted black line), $A = 10$ (dashed blue line), $A = 50$ (dot-dashed magenta) and $A = 100$ (green full line). Panel B depicts the same conditions except for $\theta = 8$. Short horizontal red lines indicate critical values at the weak selection limit obtained from (A2) in the paper. Each curve has A fixed, but to compare different values of A, the product δA is a natural measure of strength of selection, and is used in the horizontal axis.

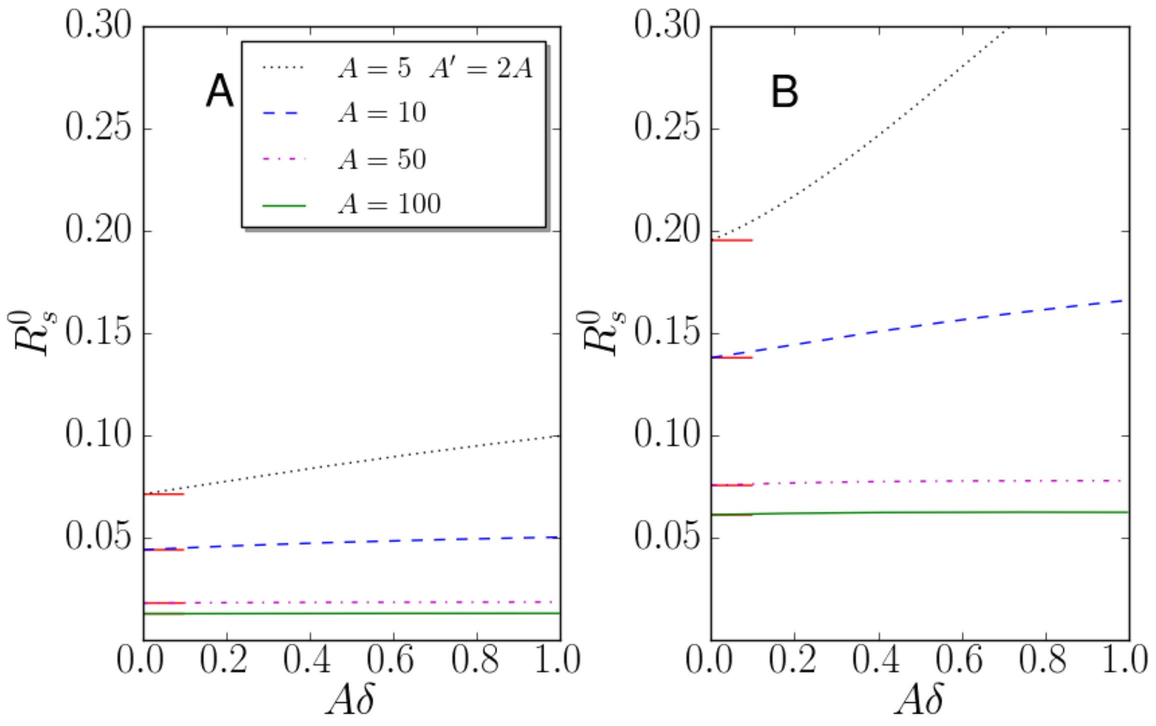

Figure S8: Threshold model (Example 3): Critical relatedness $R_s^0 = R^0(m_s)$ above which types A proliferate, as a function of the strength of selection δ. ($R^0(m) = (1-m)^2/(n-(n-1)(1-m)^2)$ is the relatedness obtained from neutral genetic markers; see Sections S5 and S7). Panels correspond to the same parameter values as in Fig. S7: Panel A depicts the case $n = 20$, $C = 1$, $\theta = 4$, $A' = 2A$, with, respectively from top to bottom, $A = 5$ (dotted black line), $A = 10$ (dashed blue line), $A = 50$ (dot-dashed magenta) and $A = 100$ (green full line). Panel B depicts the same conditions except for $\theta = 8$. As in Fig. S7, each curve has A fixed, but to compare different values of A, the product δA is a natural measure of strength of selection, and is used in the horizontal axis. Short horizontal red lines indicate critical values at the weak selection limit obtained from (A2) in the paper. These values are: Panel A: 0.012 , 0.017, 0.044, 0.071. Panel B: 0.061 , 0.075, 0.137, 0.194. Note the extremely low values of critical relatedness in Panel A. The large values of A can result from contingent cooperation, based on feedback, as for the IPG. For instance, suppose that a certain activity repeats itself T times over a life-cycle. Suppose also that in each repetition the payoff is well described by the threshold model. If types A discontinue the participation when their payoff in the previous round was negative (as in the IPG discussed in Fig 2 in the paper), then the resulting payoff over the T iterations is also given by a threshold model, with the same value of C, but A replaced by $(A-C)T+C$, and A' replaced with $A'T$. This gives plausibility to values of A and A' as large as those in this figure, since T can be in the hundreds, or thousands (see discussion on the IPG in the paper).

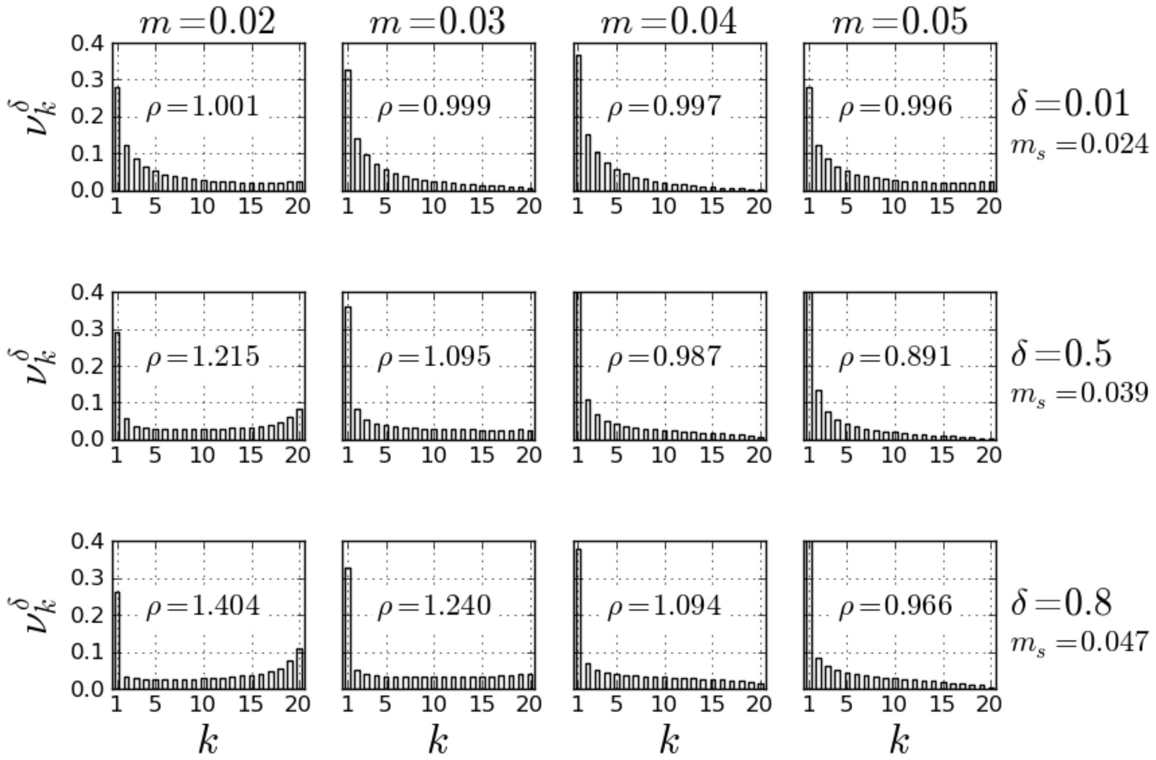

Figure S9: Public goods game (Example 1): Perron-Frobenius eigenvectors $\nu = (\nu_1, ..., \nu_n)$ represented in each box as a histogram, as a function of the strength of selection δ (rows) and of the migration rate parameter m (columns). Critical migration rates m_s are annotated in each row. Perron-Frobenius eigenvalues ρ are also provided for each box. In this picture we have $C = 1$, $B = 2$ and $n = 20$.

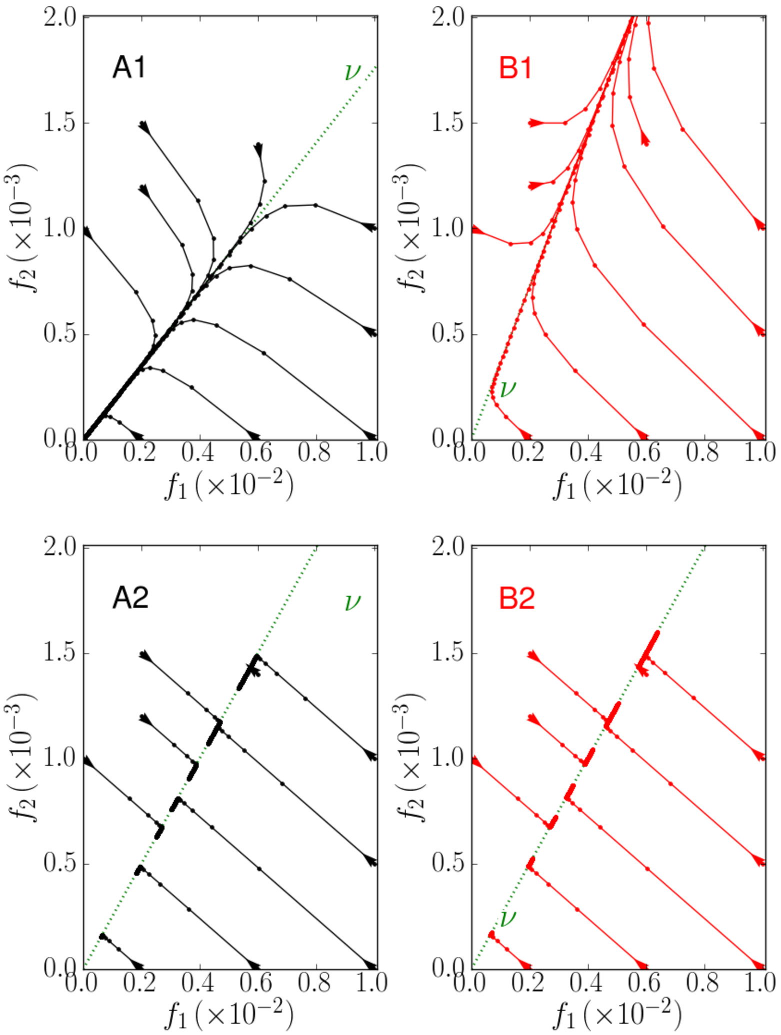

Figure S10: Self-organization of copies of A. In these pictures we have PG with $n = 2$, $C = 1$, $B = 3$, $\delta = 0.3$, resulting in $m_s = 0.2889$. Pictures show evolution of $f(t) = (f_1(t), f_2(t))$, started from several different initial distributions $f(0)$. Circles over the lines mark $f(t)$, with $t = 0, 1, \ldots, 500$ obtained by iterations of the map $f(t+1) = f(t)M(A+B)$. The direction spanned by the eigenvector ν is represented as a dotted green line. Left side (black): cases with $\rho < 1$, the allele A is eliminated; right side (red): cases with $\rho > 1$, the allele A spreads. In the top row, m is far from m_s: (A1) $m = 0.3389$, $\rho = 0.9340$, $\nu = (0.8506, 0.1494)$; (B1) $m = 0.2389$, $\rho = 1.078$, $\nu = (0.7342, 0.2658)$. In the bottom row, m is close to m_s: (A2) $m = 0.2890$, $\rho = 0.999856$, $\nu = (0.7342, 0.2658)$; (B2) $m = 0.2888$, $\rho = 1.000014$, $\nu = (0.7999, 0.2001)$. Note that in all cases $f(t)$ reaches in a few generations a steady state, in which it shrinks ($\rho < 1$), or grows ($\rho > 1$), as a multiple of ν. When m approaches m_s, the eigenvalue ρ becomes close to 1, the stationary movement along the direction given by ν slows down and the trajectories towards this direction straighten themselves, but are not slowed down.

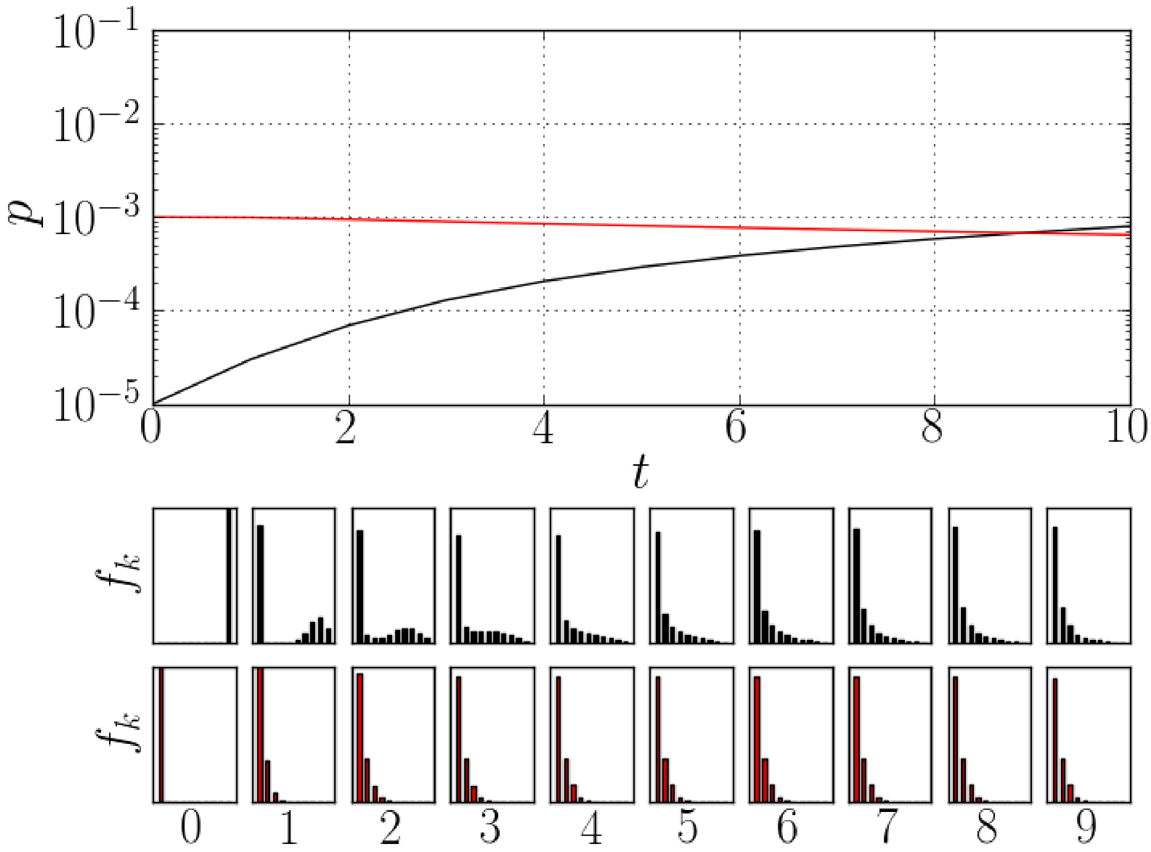

Figure S11: Self-organization of copies of A. In this picture we have IPG with $n = 10$, $C = 1$, $B = 3$, $T = 100$, $a = 2$, $\delta = 0.01$, and $m = 0.153$, slightly smaller than $m_s = 0.163$. Top part shows evolution of $p(t)$, and bottom part shows corresponding evolution of $f(t) = (f_1(t), ..., f_{10}(t))$, displayed as normalized histograms. Two initial conditions are compared: (Red) $f(0) = 10^{-2}(1, 0, ..., 0)$, so that $p(0) = 10^{-3}$. (Black) $f(0) = 10^{-5}(0, ..., 0, 1)$, so that $p(0) = 10^{-5}$. Note that from generation to generation the distribution of copies of A adjusts itself to the same stationary distribution, "losing memory of the initial distribution".

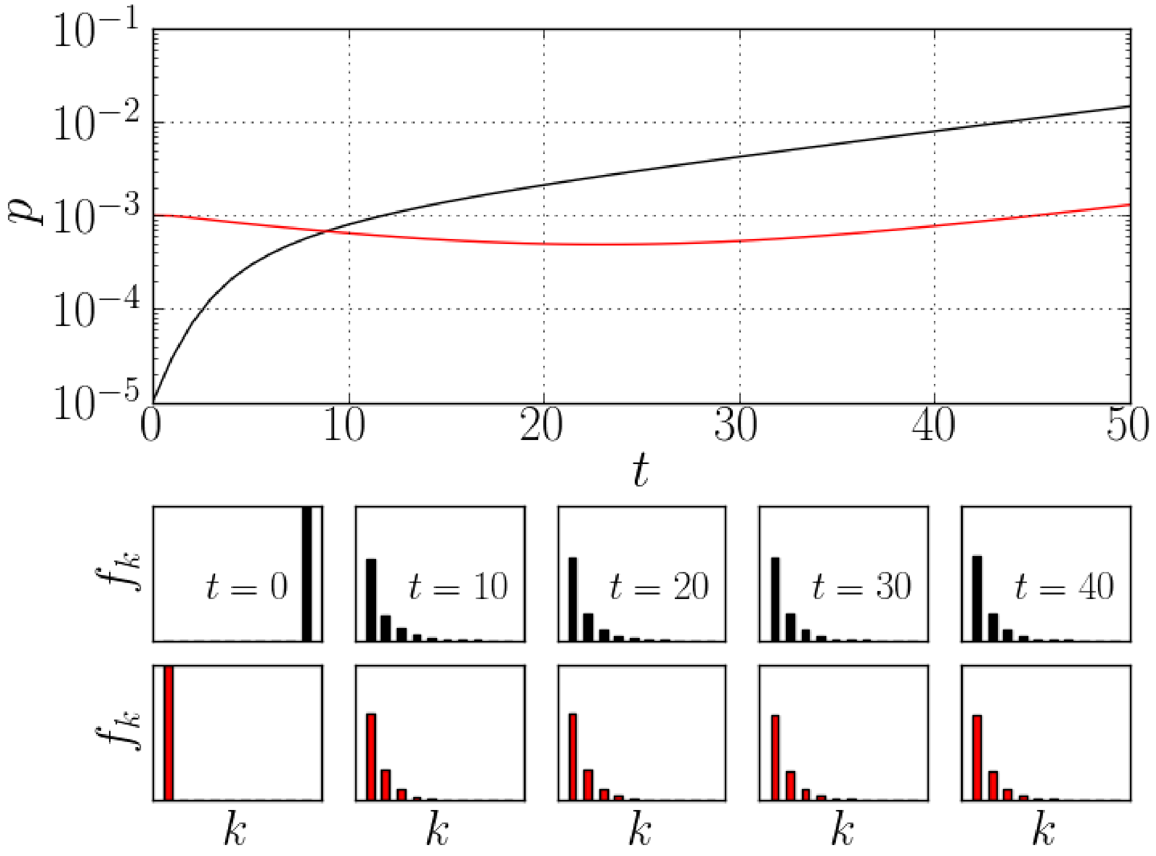

Figure S12: Self-organization of copies of A. This picture corresponds to the same model and situation described in Fig. S11, but with a different time-frame, including later times. Note that eventually the two curves of $p(t)$ become parallel straight lines, illustrating the exponential growth of $p(t)$ at rate ρ independently of the initial condition. This picture also illustrates two other important points: 1) The possible non-monotonicity of $p(t)$. 2) The fact that the asymptotic rate of growth may be smaller than the initial rate of growth. Indeed, computations of Δp only indicate the long term prospects for the allele A, when done under stationary conditions, as in (A1). The initial distribution of copies of A in the red line produces neighbor modulated fitness for A below that of allele N, so that $\Delta p(0) < 0$. In contrast, the initial distribution of copies of A in the black line produces neighbor modulated fitness for A well above that of allele N, so that not only $\Delta p(0) > 0$, but this growth happens at an unsustainably high rate. The distribution ν, towards which the copies of A self-organize is optimal for their stationary, stable, growth. This is so because (ρ, ν) is the leading eigenpair of the driving matrix $M(A + B)$: ν is the vector ν' that satisfies the eigenvalue (stationarity) equation $\nu' M(A + B) = \rho' \nu'$, with maximum ρ'.

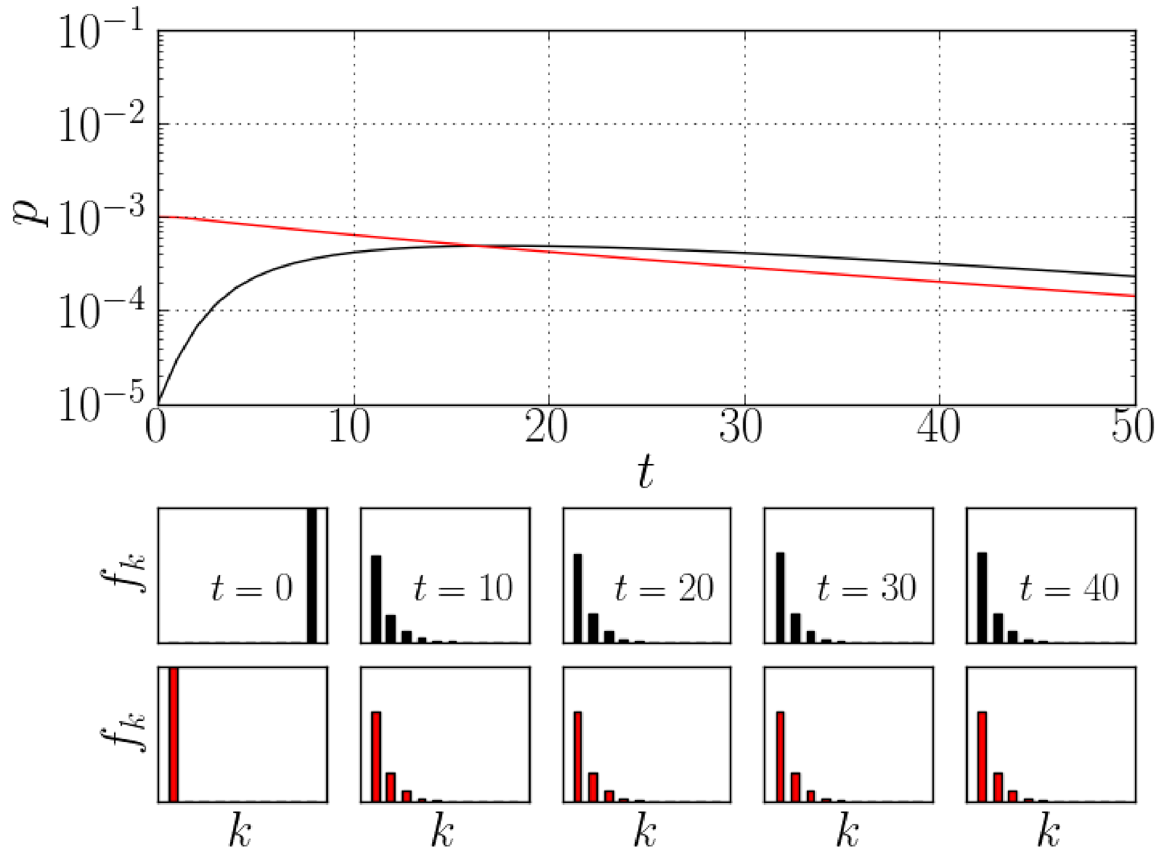

Figure S13: Self-organization of copies of A. This picture corresponds to the same model described in Fig. S11, but now $m = 0.173$ is slightly larger than $m_s = 0.163$. Note that again eventually the two curves of $p(t)$ become parallel straight lines, illustrating in this case the exponential decrease of $p(t)$ at a rate independent of the initial condition. Here again one can see that $\Delta p(0)$ is not indicative of the relevant long term evolution. The self-organized distribution ν is still optimal for the proliferation of the allele A in a stable, sustainable, fashion. But when $\rho < 1$, as in this picture, this optimal stable distribution is still not good enough for A to spread, and instead, its copies are eliminated by natural selection.

S2 Use of Perron-Frobenius Theorem

When $0 < m < 1$, the $n \times n$ matrix $M(A+B)$ has all its entries strictly positive. The Perron-Frobenius Theorem states that from this it follows that [13]
1) $M(A+B)$ has an eigenvalue ρ that is real and positive and is larger in absolute value than all the other eigenvalues of this matrix.
2) To ρ there corresponds a single left-eigenvector ν and a single right-eigenvector ζ. Both eigenvectors have all their entries strictly positive. With no loss, we normalize them as follows: $\sum_{k=1}^{n} \nu_k = 1$, and $\sum_{k=1}^{n} \nu_k \zeta_k = 1$.
3) The product $\zeta\nu$ defines a $n \times n$ matrix, with the property that for $t = 0, 1, 2, ...$,

$$[M(A+B)]^t \;=\; \rho^t \zeta \nu + E(t),$$

where the error term $E(t)$ satisfies $\max_{i,j} |E_{i,j}(t)| < \alpha^t$, for some $0 < \alpha < \rho$.

We apply the theorem to our dynamical system $f(t)$ as follows, If $f(0) \neq (0, ..., 0)$, then some of its entries are strictly positive, and hence $f(0)\zeta$ is a 1×1 matrix, with a strictly positive entry C. Therefore,

$$\begin{aligned} f(t) &= f(0)[M(A+B)]^t \;=\; f(0)[\rho^t \zeta\nu + E(t)] \\ &= \rho^t[(f(0)\zeta)\nu + \rho^{-t} f(0)E(t)] \;=\; C\rho^t(\nu + e(t)), \end{aligned} \tag{S1}$$

where the error term $e(t)$ satisfies $\max_i |e_i(t)| < \rho^{-t} \max_{i,j} |E_{i,j}(t)| < (\alpha/\rho)^t = \beta^t$, with $0 \leq \beta < 1$. Illustrations of (S1) appear in Fig. S10, Fig. S11, Fig. S12 and Fig. S13.

The error term in (A1) can now be expressed as follows. Combining the error terms in (S1) and from the approximation $\bar{W} = 1$, which is of order p (a randomly chosen individual has probability of order p of being in a group with types A), we obtain error of order $p^2 + p\beta^t$ in (A1). The left-hand-side of (A1) in this estimate refers to the linearized evolution $f(t+1) = f(t)M(A+B)$, but if one wants to estimate $\Delta(p)$ for the original non-linear dynamical system $f(t)$, then the additional error term to (A1) is also of order p^2, by the very fact that this dynamical system admits a linearization close to $(0, ..., 0)$.

S3 Focal individual. Sampling bias. Relatedness. F_{ST}. IBD

Focal individual: Suppose that in generation t a group is chosen at random, and its members are ordered in a random fashion. The chosen group is called the focal group, the first individual in the ordering is called the focal individual and the second individual in the ordering is called the co-focal individual. Note that this random experiment is equivalent to choosing a focal individual at random and then choosing a co-focal individual at random from the other $n-1$ individuals in the focal's group. We will denote by $\mathbb{P}_t$ probabilities that refer to the sampling just described, and use $\mathbb{E}_t$ for corresponding expected values. We denote by A_j the event that the jth individual in the random ordering of the members of the focal group is a type A. And we denote by I_j the indicator of the event A_j, i.e.,

I_j is the random variable that takes value 1 if A_j occurs and value 0 if its complement, A_j^c, occurs. We denote by $K = I_1 + ... + I_n$ the random number of types A in the focal group, and we set $\hat{p} = K/n$, for the random fraction of types A in the focal group. Clearly $\mathbb{E}_t(\hat{p}) = p(t)$.

Bayesian sampling bias: Conditioning on the focal being type A introduces bias on the number of types A in the focal group. This is well know and intuitive, since it is more likely that the focal individual will be type A if the focal group has many types A. The standard Bayesian computation follows. For $k = 0$, clearly $\mathbb{P}_t(K = k|A_1) = 0$, while for $k \geq 1$,

$$\begin{aligned} \mathbb{P}_t(K = k|A_1) &= \frac{\mathbb{P}_t(A_1|K = k)\, \mathbb{P}_t(K = k)}{\sum_{k'} \mathbb{P}_t(A_1|K = k')\, \mathbb{P}_t(K = k')} \\ &= \frac{(k/n)\, f_k(t)}{\sum_{k'} (k'/n)\, f_{k'}(t)} = \frac{k\, f_k(t)}{\sum_{k'} k'\, f_{k'}(t)}. \end{aligned} \tag{S2}$$

Relatedness as regression coefficient: If we define relatedness r_t as the regression coefficient of I_2 on I_1 (or equivalently, of A_2 on A_1, or of A_2^c on A_1^c), we have

$$\begin{aligned} r_t = \frac{\mathrm{Cov}_t(I_1, I_2)}{\mathrm{Var}_t(I_1)} &= \frac{\mathbb{P}_t(A_1 A_2) - (p(t))^2}{p(t)(1 - p(t))} = \frac{\mathbb{P}_t(A_2|A_1) - p(t)}{1 - p(t)} = \frac{\mathrm{Cov}_t(1 - I_1, 1 - I_2)}{\mathrm{Var}_t(1 - I_1)} \\ &= \frac{\mathbb{P}_t(A_1^c A_2^c) - (1 - p(t))^2}{p(t)(1 - p(t))} = \frac{\mathbb{P}_t(A_2^c|A_1^c) - (1 - p(t))}{p(t)}. \end{aligned} \tag{S3}$$

Equivalently, r_t can be defined by each one of the following four identities:

$$\begin{aligned} \mathbb{P}_t(A_2|A_1) &= r_t + (1 - r_t)p(t), & \mathbb{P}_t(A_2^c|A_1) &= (1 - r_t)(1 - p(t)), \\ \mathbb{P}_t(A_2|A_1^c) &= (1 - r_t)p(t), & \mathbb{P}_t(A_2^c|A_1^c) &= r_t + (1 - r_t)(1 - p(t)). \end{aligned}$$

In particular,

$$r_t = \mathbb{P}_t(A_2|A_1) - \mathbb{P}_t(A_2|A_1^c).$$

Wright's F_{ST}: The relatedness r_t is closely associated to Wright's F_{ST} statistics, defined as:

$$F_{ST,t} = \frac{\mathrm{Var}_t(\hat{p})}{\mathrm{Var}_t(\hat{p}) + \mathbb{E}_t(\hat{p}(1 - \hat{p}))}.$$

The numerator measures intergroup variability, and the second term in the denominator measures average intragroup variability. Note that the denominator can also be written as

$$\begin{aligned} \mathrm{Var}_t(\hat{p}) + \mathbb{E}_t(\hat{p}(1 - \hat{p})) &= \mathbb{E}_t((\hat{p})^2) - (\mathbb{E}_t(\hat{p}))^2 + \mathbb{E}_t(\hat{p}) - \mathbb{E}_t((\hat{p})^2) \\ &= -(p(t))^2 + p(t) = p(t)(1 - p(t)) = \mathrm{Var}_t(I_1), \end{aligned}$$

showing that it measures total variability in the population. To see how $F_{ST,t}$ relates to r_t, we use (S3) to write

$$\begin{aligned}\mathrm{Var}_t(K) &= \mathrm{Var}_t(I_1 + \ldots + I_n) = n\,\mathrm{Var}_t(I_1) + n(n-1)\,\mathrm{Cov}_t(I_1, I_2) \\ &= n\mathrm{Var}_t(I_1) + n(n-1)\,r_t\,\mathrm{Var}_t(I_1) = n\mathrm{Var}_t(I_1)\,(1 + (n-1)r_t).\end{aligned}$$

Since $\hat{p} = K/n$, we have $\mathrm{Var}_t(\hat{p}) = \mathrm{Var}_t(K)/n^2$, and we obtain

$$F_{ST,t} = \frac{1 + (n-1)\,r_t}{n}, \qquad \text{or, equivalently,} \qquad r_t = \frac{n\,F_{ST,t} - 1}{n-1}. \tag{S4}$$

In particular, when n is large $F_{ST,t}$ is close to r_t.

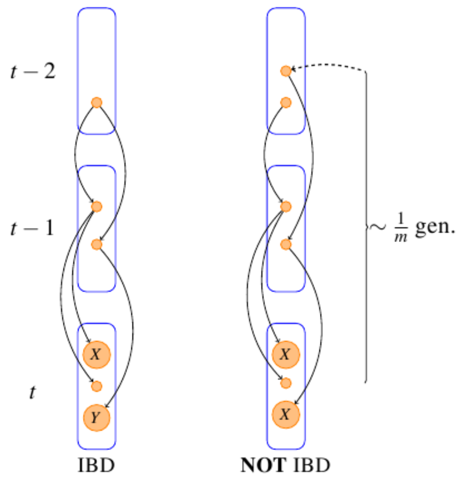

Figure S14: This diagram illustrates the concept of identity by descent (IBD) in the 2lFW. Two individuals X an Y in a given group in generation t, regardless of their type, are identical by descent (IBD) if their lineages, when followed back in time, coalesce before a migration event (indicated by a dashed arrow in the figure in the right panel). Considering a migration rate of m, migration typically takes place within a random number, of order $1/m$ of generations back.

Identity by descent: The 2lFW allows for a very natural definition of "kin" and "genetical identity by descent", IBD for short (see Fig. S14). Say that two individuals in the same generation are l-kin in case they share a common ancestor at most l generations in their past. (The 1-kin of an individual are its siblings, its 2-kin are its siblings and cousins, etc.) Because the number of groups g is large, individuals from different groups have negligible

probability of being l-kin, unless l is comparable to g. As for individuals in the same group, migration events in their lineages play a major role in their being or not kin. If, when we follow their lineages back in time, we find a migration event in one of these lineages before they coalesce, then we know that the probability that they will coalesce in a time that is much shorter than g is negligible. With this in mind, say that two individuals in the same generation are IBD in case following their lineages backwards in time, they coalesce before (in the backwards sense) a migration event happens in either one. From the discussion above, we know that being IBD in the 2lFW framework is essentially the same as being l-kin for some l that may have to be large, but is not comparable to g.

We denote by D the event that the focal and the co-focal individuals are IBD, and define genetic relatedness for the allele A by

$$R_t \;=\; I\!P_t(D|A_1).$$

Case of low prevalence: We suppose now that $I\!P_t(A_1) = I\!P_t(A_2) = p(t) = p << 1$. Then, neglecting terms of order p, we have, from (S3), $r_t = I\!P(A_2|A_1)$.

To relate now r_t to R_t, we proceed as follows. It is clear that $I\!P_t(A_2|DA_1) = 1$. On the other hand, if the event D^c happened, the type of the co-focal was sampled, independently of the type of the focal, from the whole population in a generation $t - s$, when there was a migration event in the lineage of the co-focal. Since migration occurs at fixed rate m, s is of the order of the constant $1/m$, and the frequency of types A in generation $t - s$ was of the same order of p. This implies that $I\!P_t(A_2|A_1D^c)$ is of order p, and hence, neglecting again terms of order p, $I\!P_t(A_2|A_1) = I\!P_t(A_2|DA_1)\, I\!P_t(D|A_1) + I\!P_t(A_2|D^cA_1)\, I\!P_t(D^c|A_1) = 1 \cdot I\!P_t(D|A_1) = R_t$. We have now, using also (S4),

$$R_t = \; I\!P_t(A_2|A_1) \;=\; r_t \;=\; \frac{n\,F_{ST,t} - 1}{n-1}, \qquad \text{when } p(t) << 1. \tag{S5}$$

Case of low prevalence in steady state: We suppose now the conditions of (A1): in addition to $p(t) << 1$, also $t >> 1$. Then we have the steady state relation $f(t) = C\rho^t\nu$, and (S5) and (S2) yield

$$\begin{aligned} R_t \;=\; I\!P_t(A_2|A_1) \;&=\; \sum_{k=1}^{n} I\!P_t(A_2|A_1, K=k)\, I\!P_t(K=k|A_1) \\ &=\; \frac{\sum_{k=1}^n \frac{k-1}{n-1}\, k\, f_k(t)}{\sum_{k=1}^n k\, f_k(t)} \;=\; \frac{1}{n-1}\, \frac{\sum_{k=1}^n (k-1)\, k\, \nu_k}{\sum_{k=1}^n k\, \nu_k} \;=:\; R_{\text{ses}}, \end{aligned} \tag{S6}$$

when $p(t) << 1$ and $t >> 1$.

(The symbol =: means that a new notation is being defined on its right side. The meaning of the subscript ‘ses’ will be explained in Section S9.)

S4 Linear payoff function

Suppose that $v_k^A = -C + B(k-1)/(n-1)$, as in the public goods game. Note that any linear function of k can be written in this form. We will not make any assumption on v_k^N, or on the values of C and B. Combining the notation introduced in the derivation of (A1) and that introduced in Section S3, we have $W^A = 1 + \delta \mathbb{E}_t(v_K^A | A_1)$. When $p << 1$ we have, neglecting terms of order p, $\bar{W} = W^N = 1$, and therefore

$$
\begin{aligned}
W^A - \bar{W} &= \delta\, \mathbb{E}_t(-C + B(K-1)/(n-1) | A_1) \\
&= \delta\, \mathbb{E}_t\left(-C + B\,\frac{\sum_{j=2}^n I_j}{n-1} \middle| A_1\right) \;=\; \delta\,\left(-C + B\,\frac{\sum_{j=2}^n \mathbb{E}_t(I_j|A_1)}{n-1}\right) \\
&= \delta\,(-C + B\mathbb{P}_t(A_2|A_1)) \;=\; \delta\,(-C + BR_t),
\end{aligned}
$$

where we used (S5). Hence, using $\bar{W}\Delta p = p(W^A - \bar{W})$, and neglecting terms of order p^2, we have

$$\Delta p \;=\; p\,\delta\,(-C + BR_t),$$

in this case. Note that here we did not need the assumption that $t >> 1$, but the relatedness R_t is time-dependent, even if selection is weak. If we now also assume $t >> 1$, then, from (S6),

$$\Delta p \;=\; p\,\delta\,(-C + BR_{\text{ses}}). \tag{S7}$$

S5 Limit of weak selection. IBD distribution π as a needed extension of relatedness

Separation of time scales: In this section, we will denote by $\text{Bin}(N, q)$ a random variable with binomial distribution, corresponding to N independent attempts, each one with probability q of success. When $\delta = 0$, we have $w_k^A = w_k^N = \bar{w}_k = 1$, for all values of k. The 2lFW evolves in this case by neutral genetic drift (A is a neutral marker). In this case when $p << 1$, we have $f(t+1) = f(t)M^0(A+B)$, where $(M^0)_{k,i} \;=\; \mathbb{P}\,(\text{Bin}(n, k/n) = i)$. This yields, after some simplifications,

$$
\left(M^0(A+B)\right)_{k,j} \;=\; \begin{cases} \mathbb{P}\,(\text{Bin}(n,(1-m)k/n) = j) \;+\; mk, & \text{if } \; j = 1, \\ \mathbb{P}\,(\text{Bin}(n,(1-m)k/n) = j), & \text{if } \; j = 2, ..., n. \end{cases} \tag{S8}
$$

When $0 < p(0) << 1$ and $t >> 1$, we have from the Perron-Frobenius theorem, as in the paper and in Section S2, $f(t) = C(\rho^0)^t \nu^0$, where $C > 0$ depends on $f(0)$, ρ^0 is the Perron-Frobenius eigenvalue of $M^0(A+B)$ and ν^0 its corresponding left-eigenvector, normalized as a probability vector. But when $\delta = 0$, A and N have the same relative fitness, so the expected frequency of A cannot change. This means that $p(t) = \sum_{k=1}^n (k/n) f_k(t)$ must be a constant, and hence we must have $\rho^0 = 1$.

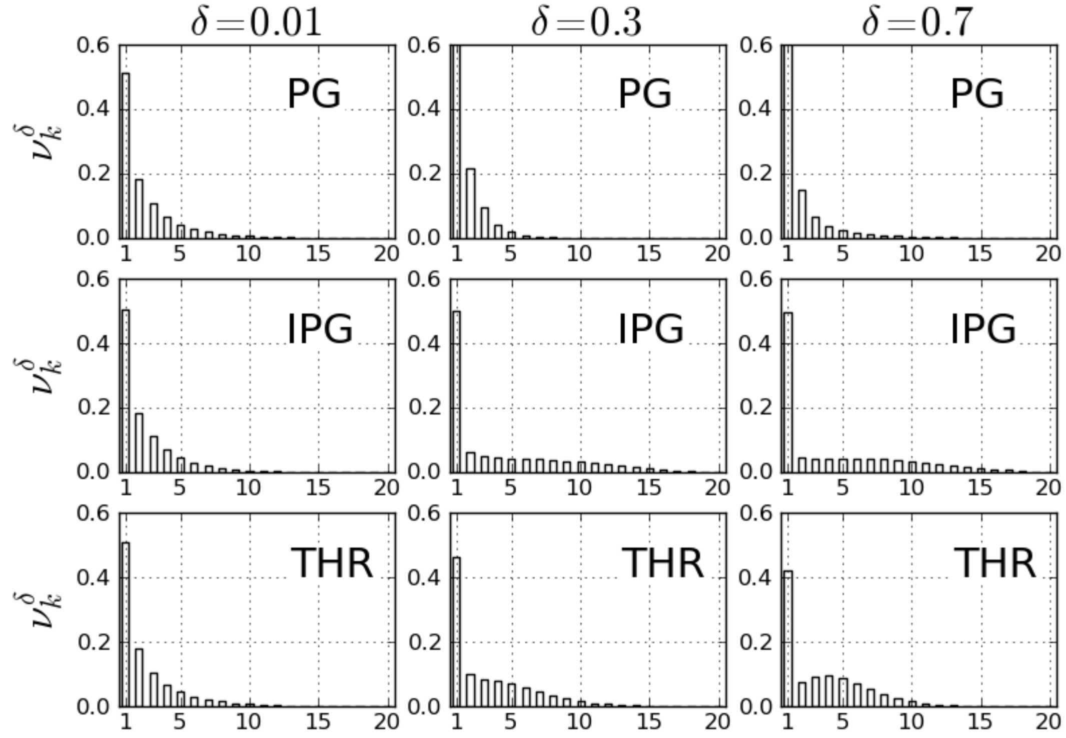

Figure S15: Perron-Frobenius eigenvectors $\nu = \nu^\delta$ for selection strengths $\delta = 0.01$ (left column), $\delta = 0.3$ (middle column) and $\delta = 0.7$ (right column). Migration rate is set to $m = 0.1$ and group sizes to $n = 20$. Each line represents a different model. The top row, labeled as PG depicts the Public Goods game (Example 1) with parameters $C = 1$ and $B = 2$. The Iterated Public Goods game (Example 2) with parameters $C = 1$, $B = 4$, $a = 4$ and $T = 10$ is shown in row at the middle, labeled as IPG. The bottom row shows Perron-Frobenius eigenvectors for the Threshold model (THR, Example 3) with $C = 1$, $A = A' = 5$ and $\theta = 4$. The leftmost column emphasizes that the weak selection limit ν_k^0 is independent of the model. In contrast, when selection is strong, ν^δ depends on the model, as illustrated in the other columns.

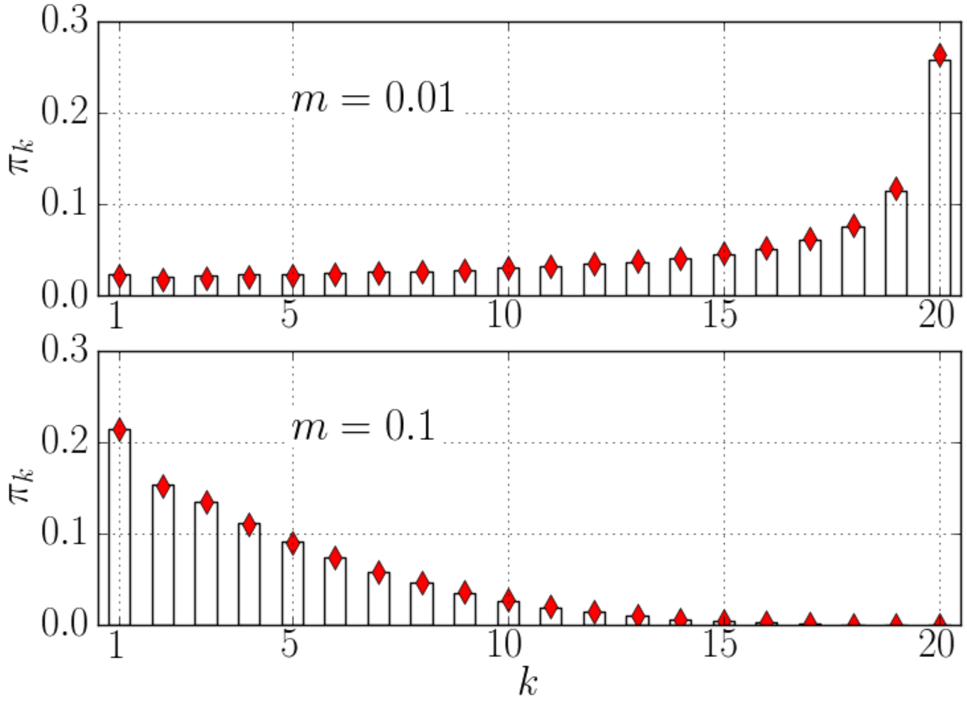

Figure S16: Distribution π_k (bars) given by $\pi = \pi Q$ and $\sum_{k=1}^{n} \pi_k = 1$, compared with $k\nu_k^\delta / \sum_{k'} k'\nu_{k'}^\delta$. Here ν^δ is the Perron-Frobenius eigenvector of $M(A+B)$, with $\delta = 0.01$, for the Threshold model (THR, Example 3) with parameters $n = 20$, $C = 1$, $A = A' = 5$ and $\theta = 4$ (red diamonds). The comparison is repeated for migration rates $m = 0.01$ (top panel) and $m = 0.1$ (bottom panel).

We want to think of the case of weak selection, i.e., $0 < \delta << 1$, as a perturbation of the case with $\delta = 0$. We consider for this purpose the limit $\delta \to 0$. By continuity of eigenvalues and eigenvectors, we have then $\rho \to 1$ and $\nu \to \nu^0$. Note that, since ν^0 does not depend on v_k^A and v_k^N, the dependence of ν on these payoff functions should vanish as $\delta \to 0$ (see Fig. S15, for an illustration).

This is the well known separation of time scales that occurs under weak selection (see, e.g., [14], [11]). Most of the time in most places evolution is occurring as if δ were 0, i.e., via neutral genetic drift. Only occasionally events happen that are caused by the slight differences in fitness of the individuals. Quantitatively, demographics operates even when $\delta = 0$, in times of order $1/m$, while selection operates in times of order $1/\delta$.

When $0 < \delta << 1$, the right hand side of (A1) can be well approximated by $\delta \frac{\sum_{k=1}^{n} k\nu_k^0 v_k^A}{\sum_{k=1}^{n} k\nu_k^0}$.

The error term in this approximation is of order δ^2, since the error in replacing ν with ν^0 is of order δ. Define now $\pi_k = k\nu^0_k / \sum_{i=1}^n i\nu^0_i$. Then (A1) yields, in good approximation, $\Delta p = p\delta \sum_{k=1}^n \pi_k v^A_k$. The quickest way to see that π is the invariant distribution of the Markov chain driven by Q, as introduced in (A2), is as follows. Note that from (S8) and the definition of Q, we have $j(M^0(A+B))_{k,j} = kQ_{k,j}$. Therefore, the eigenvalue equation $\nu^0 M^0(A+B) = \nu^0$ is equivalent to the equation $\pi Q = \pi$. (See Fig. S16 for an illustration.) This completes the proof of the claims pertaining to (A2) in the paper. But the last argument above hides the reasons for these results, and were not our original arguments. In the next subsection a less formal but more intuitive line of argumentation will be presented.

The Markov chain driven by Q can be described as follows. If at time t it is in state $X_t \in \{1, ..., n\}$, then at time $t+1$ it will take the value $X_{t+1} = 1$ with probability m, or the value $X_{t+1} = 1 + \mathrm{Bin}(n-1, (1-m)X_t/n)$ with probability $1-m$. We will explain next how this relates to IBD and how it makes the relationship between ν and this Markov chain intuitive.

Markov chain driven by Q and IBD: From this point on, in this section, we suppose that $\delta = 0$, except when stated otherwise. As before, suppose that a focal group is chosen at random in generation t, and that a focal individual from this group is then also chosen at random. Define K^D as the random variable that gives the number of individuals in the focal group that are IBD to the focal individual, herself included in the counting. Then we claim that K^D evolves from generation to generation as a Markov chain driven by Q. To see this (see Fig. S17), note that if the focal is a migrant, then she is only IBD to herself in her group, while if she is not a migrant, then she is IBD to herself and to each one of the other members of her group that are not migrants and that have a mother who is IBD to her mother. Since the assignment of mothers is given by the intragroup Fisher-Wright process (with no selection, since $\delta = 0$), we have for K^D the same probabilistic evolution as for the chain X_t above.

We want to understand now, on intuitive grounds, why ν^0 relates to the invariant distribution of the Markov chain driven by Q in the way we found computationally in the previous subsection. Suppose that $0 < p(0) << 1$ and $t >> 1$, so that $f(t) = C\nu^0$. Suppose that at time t we choose a random focal group and from that group a random focal individual. We condition now on the event A_1, that the focal individual is type A. Because $p(t) = p(0) << 1$, it is very likely then that the number of types A in the focal group is $K = K^D$, since individuals in the focal group who are not IBD to the focal individual have only probability p of being type A, while those who are IBD to the focal must be type A. Because we are under neutral drift, with $\delta = 0$, conditioning on A_1 does not affect lineages. This means that

$$\mathbb{P}_t(K = k | A_1) = \mathbb{P}_t(K^D = k | A_1) = \mathbb{P}_t(K^D = k).$$

By Bayesian bias in sampling, the left-hand side is $kf_k(t))/(\sum_{i=1}^n if_i(t))$ $= k\nu^0_k / (\sum_{i=1}^n i\nu^0_i) = \pi_k$, where the first equality is a good approximation when $0 < p(0) <<$

1 and $t >> 1$ and the second equality is the definition of π. On the other hand, since $t >> 1$, the right-hand-side is close to the invariant distribution of the Markov chain driven by Q, as we saw in the previous paragraph. This shows that π is that invariant distribution.

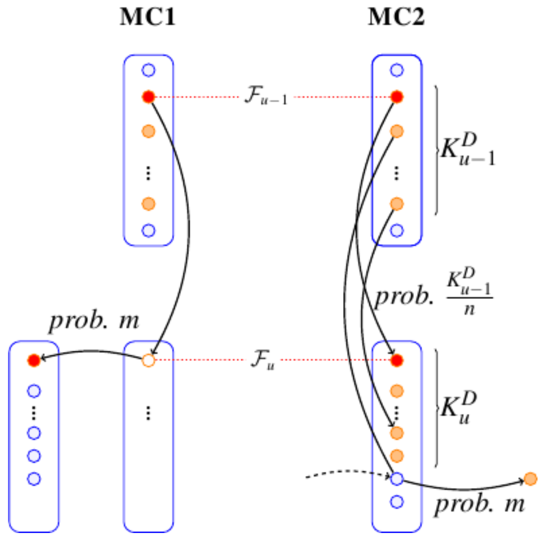

Figure S17: This diagram illustrates why K^D evolves as a Markov chain driven by Q. In this picture K_u^D represents the number of individuals that are IBD to the focal individual $\mathcal{F}_u$, in generation u (red circle). Two scenarios are discernible. MC1 (left panel): the focal individual is a migrant. This happens with probability m and implies that $K_u^D = 1$. MC2 (right panel): the focal individual $\mathcal{F}_u$ is not a migrant, and she is a child of $\mathcal{F}_{u-1}$. Each individual in the focal group in generation u choses a mother from the group of $\mathcal{F}_{u-1}$ in the previous generation with uniform probability, as $\delta = 0$. With probability K_{u-1}^D/n the chosen mother is IBD to $\mathcal{F}_{u-1}$ (orange circles) and, consequently, her children are also IBD to $\mathcal{F}_u$, provided that they are not migrants. In this case, the number of individuals in generation u that are IBD to the focal is, therefore, 1 (for the focal individual herself) plus a number of individuals given by a binomial random variable with probability of success $(1-m)K_{u-1}^D/n$ in $n-1$ trials.

Moments of π: The Markov chain introduced above can be used to write recursions for quantities of interest. For instance, this method can be used for computing the moments of the distribution π, $\mathcal{M}_l = \sum_k k^l \pi_k$, $l = 1, 2, \ldots$.

To express the lth moment, $\mathcal{M}_l$, in terms of $\mathcal{M}_j$, $j = 1, 2, \ldots, l-1$, we will use the following fact. If Y has a Bin(N, q) distribution, then

$$\mathbb{E}(Y^l) \; = \; \sum_{j=1}^{l} S(l,j)\, N_j\, q^j,$$

where $N_j = N(N-1)\cdots(N-j+1)$, and the Stirling number of the second kind, $S(l,j)$, is the number of ways in which a set with l elements can be partitioned into j non-empty sets. Clearly $S(l,1) = S(l,l) = 1$, for all l. They are also known to satisfy

$$S(l,j) \;=\; \frac{1}{j!}\sum_{i=0}^{j}\begin{pmatrix} j \\ i \end{pmatrix}(j-i)^l.$$

The first few values of $S(l,j)$ are: $S(1,1)=1; S(2,1)=1, S(2,2)=1; S(3,1)=1, S(3,2)=3, S(3,3)=1; S(4,1)=1, S(4,2)=7, S(4,3)=6, S(4,4)=1; S(5,1)=1, S(5,2)=15, S(5,3)=25, S(5,4)=10, S(5,5)=1; \ldots$

For our purpose, it is convenient to write $s = 1-m$, and to consider the stationary Markov chain, i.e., the case in which $\mathbb{P}(X_0 = k) = \pi_k$, so that also $\mathbb{P}(X_1 = k) = \pi_k$. Look now at the random variable $(X_1 - 1)^l$. With probability m it takes the value 0, and with probability s it takes the value $(\mathrm{Bin}(n-1, sX_0/n))^l$. Therefore

$$\mathbb{E}((X_1-1)^l) \;=\; s\sum_{j=1}^{l} S(l,j)\,(n-1)_j\, s^j\,\mathbb{E}(X_0)^j/n^j.$$

But since X_0 and X_1 both have distribution π, we obtain

$$\sum_{j=0}^{l}\begin{pmatrix} l \\ j \end{pmatrix}(-1)^{l-j}\,\mathcal{M}_j \;=\; \sum_{j=1}^{l} S(l,j)\,\frac{(n-1)_j}{n^j}\,s^{j+1}\,\mathcal{M}_j.$$

This provides us with the aimed recursion:

$$\mathcal{M}_l \;=\; \frac{(-1)^{l+1} \;+\; \sum_{j=1}^{l-1}\left\{(-1)^{l-j+1}\begin{pmatrix} l \\ j \end{pmatrix} \;+\; S(l,j)\,\frac{(n-1)_j}{n^j}\,s^{j+1}\right\}\mathcal{M}_j}{1 \;-\; \frac{(n-1)_l}{n^l}\,s^{l+1}}. \tag{S9}$$

For $l=1$, (S9) reduces to

$$\mathcal{M}_1 \;=\; \frac{n}{n-(n-1)(1-m)^2}. \tag{S10}$$

Relatedness: The mean $\mathcal{M}_1$ of π is directly related to the relatedness in groups under $\delta = 0$, and demographic equilibrium ($t >> 1$), that we will denote by R^0. First we observe that when $\delta = 0$, conditioning on the focal being type A does not change the demographic distribution, hence $R^0 = \lim_{t\to\infty}\mathbb{P}_t(D|A_1) = \lim_{t\to\infty}\mathbb{P}_t(D)$, where, as before D is the event that the focal and the co-focal individuals are IBD. Recall the enumeration of the members of the focal group, in which the first individual is the focal. Now decompose $K^D - 1 = I_2^D + \ldots + I_n^D$, where, $I_j^D = 1$, if the jth individual in this ordering is IBD to the focal, and $I_j^D = 0$, otherwise. This yields $\mathbb{E}_t(K^D - 1) = \mathbb{E}_t(I_2^D) + \ldots + \mathbb{E}_t(I_n^D) =$

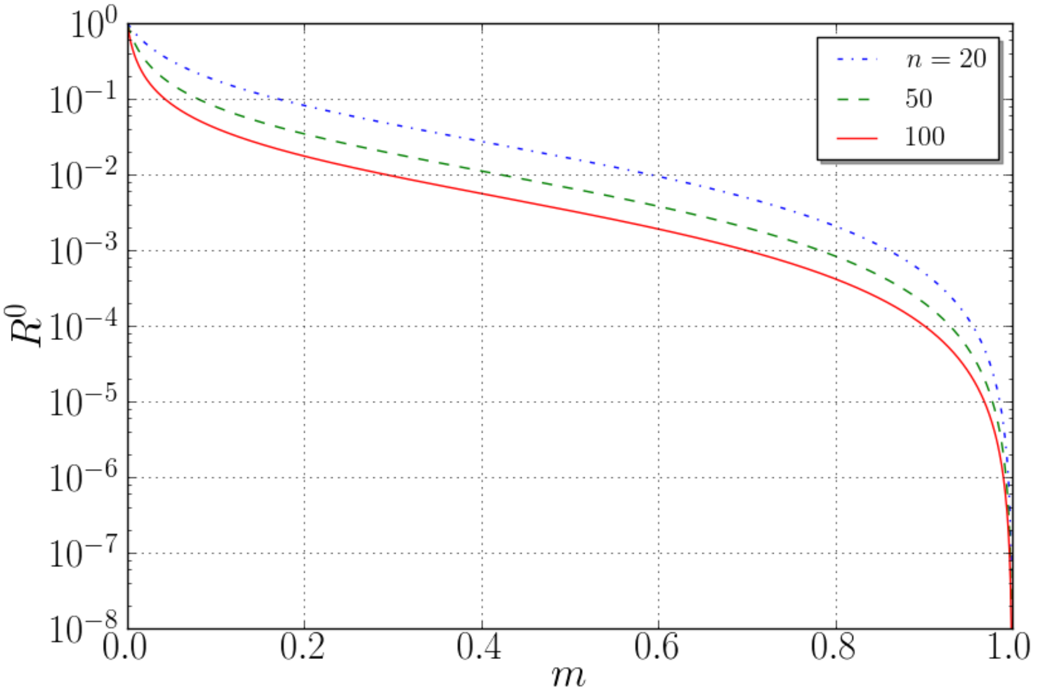

Figure S18: Relatedness R^0 as a function of migration rate m, under neutral drift, $\delta = 0$, as given by (S11). From top to bottom, $n = 20$ (dot-dashed blue line), $n = 50$ (dashed green line) and $n = 100$ (full red line).

$(n-1)\mathbb{P}_t(D)$, and therefore $\mathbb{P}_t(D) = (\mathbb{E}_t(K^D)-1)/(n-1)$. Putting these pieces together yields

$$R^0 = \lim_{t\to\infty} \mathbb{P}_t(D) = \lim_{t\to\infty} \frac{\mathbb{E}_t(K^D)-1}{n-1} = \frac{\mathcal{M}_1 - 1}{n-1} = \frac{(1-m)^2}{n-(n-1)(1-m)^2}. \quad \text{(S11)}$$

(See Fig. S18.) When m is small, (S11) yields the approximation

$$R^0 \approx \frac{1}{1+2nm}. \quad \text{(S12)}$$

This is a well known result by Wright for the infinite islands model, for haploid individuals. It is important to clarify that even when $\delta = 0$, our 2lFK framework is not identical to the infinite islands model. In that model there are a large number g (to be taken to ∞ in the computations) of islands, each one with n individuals. In generation $t+1$ the n individuals in each island choose, independently, a parent from the individuals in the same island in generation t. This is followed by migration at rate m, that is implemented in

exactly the same way as in the 2lFW framework. In contrast with what happens in 2lFW, each island is "parent" to exactly one island in the next generation. In 2lFW, when $\delta = 0$, each group is parent to a random number, with distribution $\text{Bin}(g, 1/g)$, of groups. In spite of this difference, it is not simply a coincidence that lead to the same formula (S11) in both frameworks. They share the same coalescence structure, when we ask ourselves questions related to IBD. Not only is R^0 identical in these frameworks, but so is also the distribution π. Indeed, when we follow lineages backwards in time, until there are migration events, it does not matter if the group that we are following from generation to generation is the same (as in the infinite islands case) or changes (as in our 2lFW case).

For arbitrary payoff functions, v_k^A and v_k^N, we have, from (S6),

$$\begin{aligned}\lim_{\delta\to 0} R\text{ses} &= \lim_{\delta\to 0} \frac{1}{n-1}\frac{\sum_{k=1}^n (k-1)\,k\,\nu_k}{\sum_{k=1}^n k\,\nu_k} = \frac{1}{n-1}\frac{\sum_{k=1}^n (k-1)\,k\,\nu_k^0}{\sum_{k=1}^n k\,\nu_k^0} \\ &= \frac{1}{n-1}\sum_{k=1}^n (k-1)\,\pi_k = \frac{\mathcal{M}_1-1}{n-1} = R^0. \end{aligned} \quad \text{(S13)}$$

Mnemonic recursions: There is a well known (in the infinite island case, but the same applies to 2lFW) and simpler way to derive (S11). If a focal and a co-focal individuals are chosen from the same focal group in generation $t >> 1$, then the probability R^0 that they are IBD can be calculated as follows. For them to be IBD neither can be a migrant (probability $(1-m)^2$). Given that they are not migrants, they will be IBD if they have the same mother (probability $1/n$), or if they have distinct mothers and their mother are IBD (probability $(1-1/n)R^0$, using the fact that $t >> 1$ implies demographic equilibrium). Combining these, we have

$$R^0 = (1-m)^2((1/n) + (1-1/n))R^0, \quad \text{(S14)}$$

which yields (S11).

The very same kind of powerful reasoning can be used to obtain the recursions for $\pi_1, ..., \pi_k$ that are summarized in the equation $\pi Q = \pi$. This is done in the second derivation of (A2) in the paper, and reproduced next, so that its parallel with the derivation of (S14) becomes apparent. For this we will use the standard Kronecker notation $\delta_{i,j} = 1$ if $i = j$ and $\delta_{i,j} = 0$ if $i \neq j$. Now, the probability π_k that the focal is IBD to exactly $k-1$ other members of her group is $\delta_{k,1}$ if the focal is a migrant (probability m), while if she is not a migrant (probability $1-m$), then we have to consider how many individuals in her mother's group were IBD to her mother. If, counting her mother, that number was j (probability π_j, assuming demographic equilibrium) then the probability that the focal is IBD to exactly $k-1$ other members of her group is equal to the probability that of the $n-1$ other members of the focal's group, exactly $k-1$ chose one of the j candidates to mother that were IBD to the focal's mother, and moreover did not become a migrant (probability $I\!P(\text{Bin}(n-1, (1-m)j/n) = k-1)$). Combining these pieces, we have

$$\pi_k = m\,\delta_{k,1} + (1-m)\sum_{j=1}^n \pi_j\, I\!P(\text{Bin}(n-1, (1-m)j/n) = k-1). \quad \text{(S15)}$$

This is exactly the same as the set of equations $\pi Q = \pi$. And Fig. S17 is a diagram of that reasoning.

When is π needed over R^0? Precisely when v_k^A is not a linear function of k. Suppose that $v_k^A = -c + b_1 k + b_2 k^2 + \cdots + b_l k^l$. Then the weak selection viability condition derived from $\Delta p > 0$ in (A2) becomes

$$c < b_1 \mathcal{M}_1 + b_2 \mathcal{M}_2 + \cdots + b_l \mathcal{M}_l, \tag{S16}$$

involving moments of π. In the linear case $l = 1$, this condition becomes $c < b_1(1 + (n-1)R^0)$, that only depends on R^0. If in this case we write $v_k^A = -C + B(k-1)/(n-1)$, corresponding to $c = C + B/(n-1)$ and $b_1 = B/(n-1)$, then this viability condition takes the familiar form $C < BR^0$. This is the same that one obtains from combining (S7) with (S13). In the next section we will study the limit (S18), under which (S16) simplifies considerably, becoming equivalent to the condition $\Delta(p) = 0$ in (A4).

Another good illustration of the need to use π rather than R^0 is given by the threshold model, Example 3. In this case the viability condition derived from $\Delta p > 0$ in (A2) becomes

$$C \; < \; A \sum_{k=\theta}^{n} \pi_k. \tag{S17}$$

In this case, the relevant information contained in π, rather than being in R^0 or in higher moments, is in the tail of that distribution. The results from the next section will lead to a major simplification to (S17), in the limit (S18), assuming that θ/n converges to some $\widetilde{\theta}$, so that the conditions of (A3) are satisfied. (See second subsection of Section S7.)

S6 Limit of large n and small m under weak selection. IBD distribution is close to beta distribution

Convergence of π to beta distribution: In this section we will continue to assume that selection is weak and we will study the limit in which

$$n \to \infty, \quad m \to 0 \quad \text{and} \quad nm \to \widetilde{m}, \quad \text{so that} \quad R^0 \to 1/(1+2\widetilde{m}) = \widetilde{R}^0, \tag{S18}$$

where we used (S11), (S12). We will suppose that $0 < \widetilde{m} < \infty$. Suppose also that K^D is a random variable with distribution π, i.e., $\mathbb{P}(K^D = k) = \pi_k$. Then we will show that

$$\mathbb{P}(K^D/n > x) \;\to\; \int_x^1 f_{\widetilde{m}}(x')dx' \;=\; (1-x)^{2\widetilde{m}}, \tag{S19}$$

for arbitrary $0 \leq x \leq 1$. This means that K^D/n converges in distribution to a beta distribution with parameters $\alpha = 1$ and $\beta = 2\widetilde{m}$, which has density $f_{\widetilde{m}}(x) = 2\widetilde{m}\,(1-x)^{2\widetilde{m}-1}$, $0 < x < 1$. (See Fig. S19 for illustrations.)

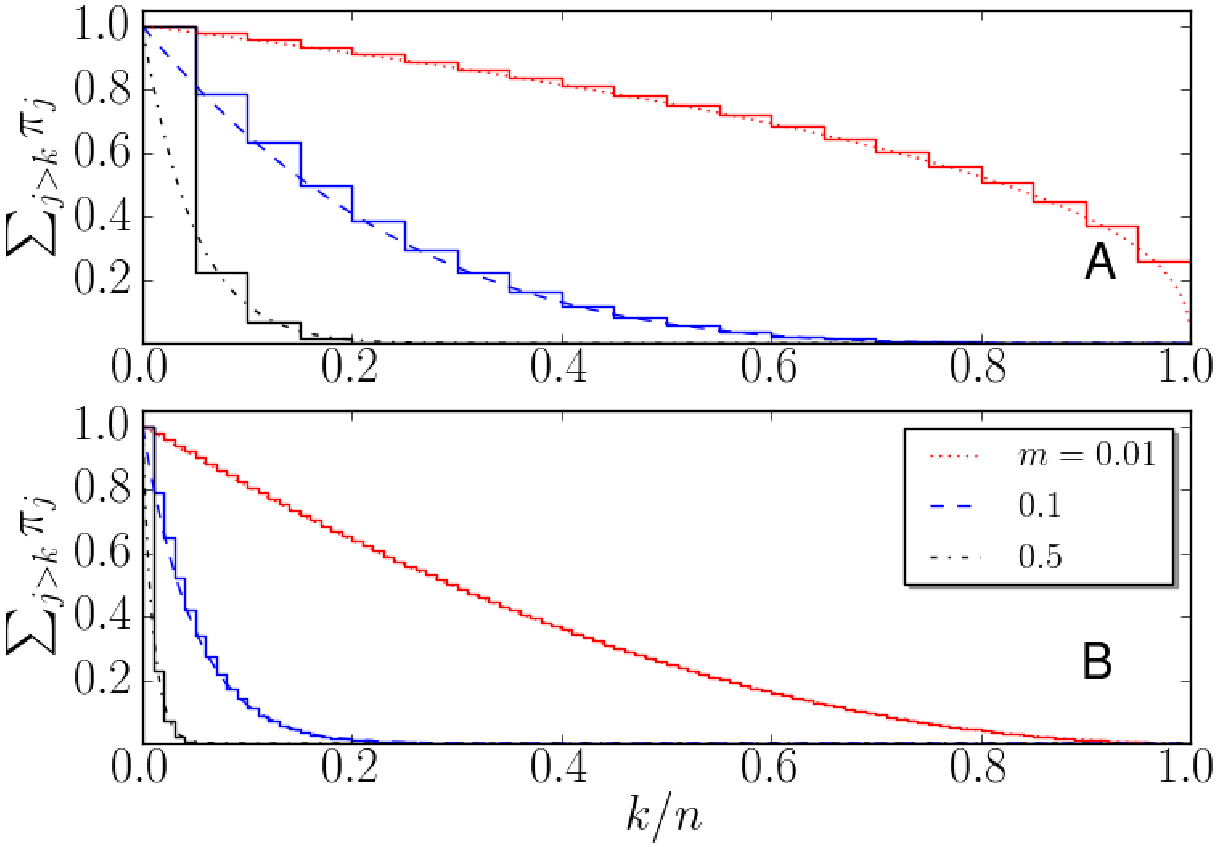

Figure S19: Limit of large n and small m under weak selection. This figure compares tail probabilities for the distribution π (stairs) and for Beta distributions with parameters $\alpha = 1$ and $\beta = 2mn$. Panel A shows the case $n = 20$ for, from top to bottom, $m = 0.01$ (red dotted line), $m = 0.1$ (blue dashed line) and $m = 0.5$ (black dot dashed line). Panel B depicts the same scenarios for the case $n = 100$.

To prove (S19), it is sufficient to show that the moments of K^D/n converge to those of the beta distribution (see, e.g., Section 2.3.e of [4]), namely that for $l = 1, 2, ...,$

$$\frac{\mathcal{M}_l}{n^l} \longrightarrow \frac{l!}{(2\widetilde{m}+1)(2\widetilde{m}+2)\cdots(2\widetilde{m}+l)} = \widetilde{\mathcal{M}}_l. \tag{S20}$$

We will prove (S20) by induction in $l = 1, 2, ...$ First, from (S10) we obtain it in case $l = 1$. Now suppose that (S20) is true for $l = 1, 2, ..., i-1$. Then $\mathcal{M}_l/n^{i-1} \to 0$, for $l = 1, ..., i-2$,

so that from (S9) we obtain

$$\lim \frac{\mathcal{M}_i}{n^i} \;=\; \lim \frac{\left\{\begin{pmatrix} i \\ i-1 \end{pmatrix} \;+\; S(i,i-1)\,\frac{(n-1)_{i-1}}{n^{i-1}}\,s^i\right\}\,\frac{\mathcal{M}_{i-1}}{n^{i-1}}}{n\left(1 \;-\; \frac{(n-1)_i}{n^i}\,s^{i+1}\right)}.$$

But

$$\begin{aligned}\begin{pmatrix} i \\ i-1 \end{pmatrix} \;+\; S(i,i-1)\,\frac{(n-1)_{i-1}}{n^{i-1}}\,s^i \;&=\; \begin{pmatrix} i \\ i-1 \end{pmatrix} \;+\; \begin{pmatrix} i \\ 2 \end{pmatrix}\,\frac{(n-1)_{i-1}}{n^{i-1}}\,s^i \\ &\longrightarrow\; i \;+\; \frac{i\,(i-1)}{2} \;=\; \frac{i\,(i+1)}{2}.\end{aligned}$$

And

$$\begin{aligned} 1 \;-\; \frac{(n-1)_i}{n^i}\,s^{i+1} \;&=\; 1 \;-\; \left(1-\frac{1}{n}\right)\left(1-\frac{2}{n}\right)\ldots\left(1-\frac{i}{n}\right)(1\;-\;m)^{i+1} \\ &=\; \frac{1+2+\ldots+i}{n} \;+\; (i+1)\,m \;+\; \Delta(n,m) \\ &=\; \frac{i\,(i+1)}{2n} \;+\; (i+1)\,m \;+\; \Delta(n,m),\end{aligned}$$

where $\Delta(n,m)\,n \;\rightarrow\; 0$. The last three displays, and the induction hypothesis, imply that

$$\lim \frac{\mathcal{M}_i}{n^i} \;=\; \frac{\frac{i\,(i+1)}{2}\,\widetilde{\mathcal{M}}_{i-1}}{\frac{i\,(i+1)}{2} \;+\; (i+1)\,\widetilde{m}} \;=\; \frac{i}{i+2\widetilde{m}}\,\widetilde{\mathcal{M}}_{i-1} \;=\; \widetilde{\mathcal{M}}_i.$$

This proves that (S20) is true for $l = i$, completing the induction argument, and the proof of (S19).

Application: Suppose that there is a piecewise continuous and bounded function $\widetilde{v}^A_x$, $0 \le x \le 1$, such that

$$\max_{k=1,\ldots,n} \left|v^A_k - \widetilde{v}^A_{k/n}\right| \;\longrightarrow\; 0, \qquad \text{as} \;\; n \to \infty. \tag{S21}$$

Then, the weak convergence result in (S19) above, combined with the continuous mapping theorem, (2.3) in Section 2.2.b of [4], implies that, when $0 < \widetilde{m} < \infty$,

$$\sum_{k=1}^{n} v^A_k \pi_k \;\longrightarrow\; \int_0^1 \widetilde{v}^A_x\, f_{\widetilde{m}}(x)\,dx \;=\; 2\widetilde{m}\int_0^1 \widetilde{v}^A_x\,(1-x)^{2\widetilde{m}-1}\,dx, \tag{S22}$$

in the limit (S18). This proves that (A3) follows from (A2) in this limit.

S7 Critical values

Definitions: When $\rho > 1$ we are in the regime in which the fixed point $(0, ..., 0)$ of the dynamical system $f(t)$ is unstable (the allele A can invade), while, when $\rho < 1$, this fixed point is stable (the allele A cannot invade). (The boundary case $\rho = 1$ could fall in either category, depending on the model.) It is natural to define the critical value

$$m_s \ = \ \max\{m \in [0,1] : \rho \geq 1\}. \tag{S23}$$

(The subscript 's' stands for 'survival' of the allele A, when rare.) Typically (see Fig. S2), we found that $\rho(m)$ is a monotone decreasing function of m, and

$$\rho(m_s) = 1 \tag{S24}$$

has a single solution. In this case (S24) is equivalent to (S23), and the allele A can invade when $m < m_s$ and cannot invade when $m > m_s$.

Conditions (C1) and (C2) from Section S1 imply that $0 < m_s < 1$, as we show next. For this purpose, we use continuity of eigenvalues and eigenvectors. In particular $\rho(m)$ is continuous, and it is enough to observe how it behaves as m approaches 0 or 1. When $m = 1$, the matrix $A = 0$, and $M(A + B) = MB$ has only the first column not identically 0. Therefore any of its left-eigenvectors must be of the form $(a, 0, ..., 0)$. When such a vector is multiplied by MB, the result is $w_1^A(a, 0, ..., 0)$. This shows that w_1^A is the only eigenvalue of $M(A + B)$. Therefore $\rho(m)$ must converge to $w_1^A < 1$ (by (C1)), as $m \to 1$. When $m = 0$, $M(A + B) = M$ has the eigenvalue $w_n^A > 1$ (by (C2)), corresponding to the left-eigenvector $(0, 0, ..., 0, 1)$. This implies that $\rho(m)$ must be larger than 1 when m is close to 0.

The parameter $1 - m$ indicates the level of assortment in the 2lFW. Another natural parameter for this purpose, that is also in one-to-one correspondence with m, is $R^0(m)$, given by (S11) (see Fig. S18). It provides the relatedness measured using neutral genetic markers, in the same demographics in equilibrium, by means of regression coefficients, Wright's F_{ST}, or lineages (IBD). This leads to the following natural definition:

$$R_s^0 \ = \ R^0(m_s) \ = \ \frac{(1 - m_s)^2}{n - (n - 1)(1 - m_s)^2}.$$

When the allele A is under weak selection, so that m_s solves $\Delta p = 0$ in (A2), then R_s^0 gives the critical value of relatedness also if measured for the allele A itself, in demographic equilibrium. But this is not the case if A is under strong selection. Even then, R_s^0 is a meaningful way to describe the critical value of assortment needed for A to be able to invade the population, as, for instance, in Panel B of Fig. 2 in the paper.

If the allele A is under weak selection, n is large, and we have in good approximation (in the sense of (S21)) $v_k^A = \widetilde{v}_{k/n}^A$, for some piecewise continuous and bounded function $\widetilde{v}_x^A$, $0 \leq x \leq 1$, then m_s solves $\Delta p = 0$ in (A3). It is then natural to define

$$\widetilde{m}_s \ = \ \lim_{n\to\infty} nm_s, \qquad \text{and} \qquad \widetilde{R}_s^0 \ = \ \lim_{n\to\infty} R_s^0 \ = \ \frac{1}{1 + 2\widetilde{m}_s}.$$

$\widetilde{R}_s^0$ gives the critical level of relatedness in the population, measured with the allele A itself or with neutral genetic markers, that allows the allele A to invade in this case. The critical migration rate is then close to $\widetilde{m}_s/n$.

Threshold model with large n: A simple example of the use of (A3) is given by the threshold model, Example 3. Suppose that $\theta = \lceil n\widetilde{\theta}\,\rceil$, for some $0 \le \widetilde{\theta} \le 1$, where $\lceil y \rceil$ is the smallest integer larger than or equal to y. In this case, $v_k^A = \widetilde{v}_{k/n}^A$, with $\widetilde{v}_x^A = -C$, for $0 \le x < \widetilde{\theta}$, and $\widetilde{v}_x^A = -C + A$, for $\widetilde{\theta} \le x \le 1$. We can now apply (A3), when A is under weak selection, and n is large. The equation $\Delta p = 0$ can be easily integrated in this case, giving $C = A(1-\widetilde{\theta})^{2\tilde{m}_s}$, which yields

$$\widetilde{m}_s \;=\; \frac{\log(C/A)}{2\,\log(1-\widetilde{\theta})}, \qquad \text{or, equivalently,} \qquad \widetilde{R}_s^0 \;=\; \frac{1}{1+\frac{\log(C/A)}{\log(1-\widetilde{\theta})}}, \tag{S25}$$

where log can be in any base. If $\widetilde{\theta}$ is small, then we have the further approximations

$$\widetilde{m}_s \;=\; \frac{\ln(A/C)}{2\,\widetilde{\theta}}, \qquad \text{or, equivalently,} \qquad \widetilde{R}_s^0 \;=\; \frac{1}{1+\frac{\ln(A/C)}{\widetilde{\theta}}}. \tag{S26}$$

Fig. S20 illustrates the quality of these approximations to m_s and R_s^0, with $n = 20$ and $n = 100$.

In the next section a similar analysis will be carried out for the IPG (Compare Fig. S20 to Fig. S21.)

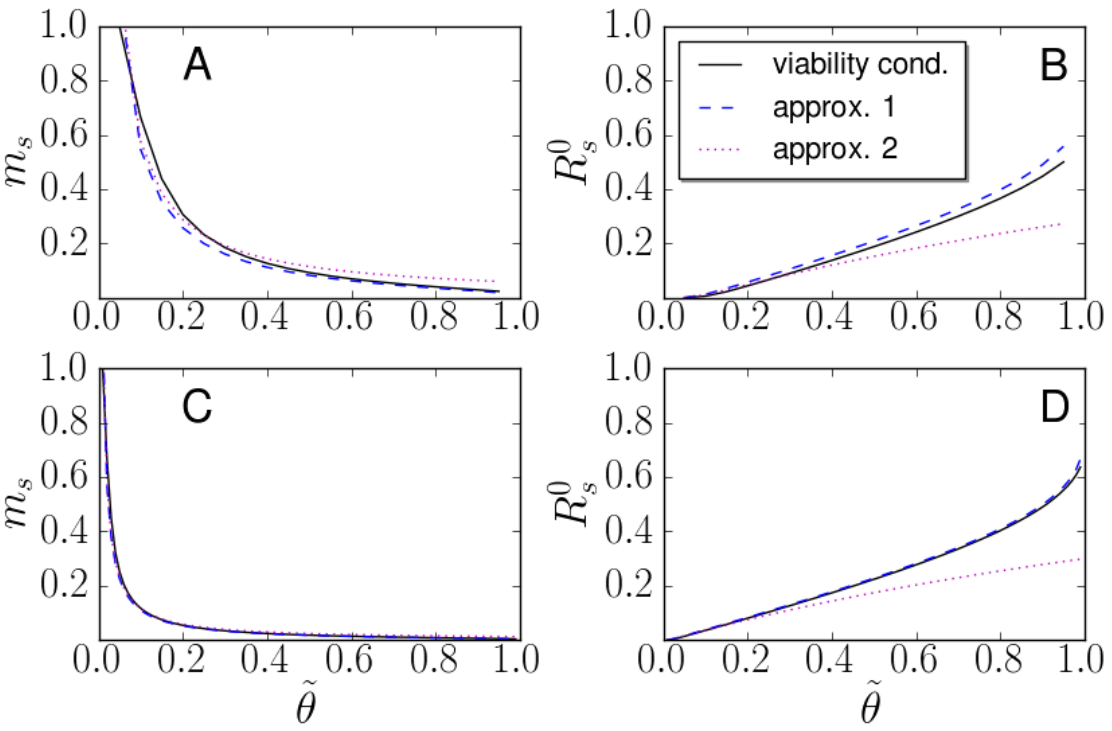

Figure S20: Limit of large n under weak selection for the threshold model (THR, Example 3). Panels represent critical migration rates (A and C) and critical relatedness (B and D) for the THR with $C = 1$, $A = A' = 10$ as a function of $\widetilde{\theta} = \theta/n$. Top panels A and B depict the case $n = 20$. Bottom panels C and D depict the case $n = 100$. In each panel critical values obtained by the viability condition under weak selection derived from (A2) (black full lines) are compared with the approximation for large n given by (S25) ($m_s = \widetilde{m}_s/n$, $R_s^0 = \widetilde{R}_s^0$, approx.1, dashed blue lines) and with the approximation (S26) (approx.2, dotted red lines).

S8 Iterated public goods game. Types A play multi-individual tit-for-tat

In this section we will analyse in detail the iterated public goods game (IPG, Exemple 2), introduced in Fig. 2 of the paper and in Section S1. This model was studied independently in [3] and in [9], in the trait-group framework. Both papers identified stable equilibria with positive fractions of types A. But they also observed that altruists could not invade when rare. In [3] an approach was then introduced to provide assortment and allow the allele A to invade when rare. The authors concluded that such invasion by allele A could only occur under very restrictive conditions, and that therefore this model and the corresponding notion of multi-individual tit-for-tat were of marginal relevance. One of our contributions in the current paper is to rectify this perception. We show here that in the 2lFW framework, the allele A can invade under very reasonable conditions. Therefore we propose that conditional mechanisms of cooperation be seriously considered. Later in this section, we will explain why the estimates in [3] contained an unreasonably pessimistic assumption, that is not supported in the 2lFW.

Suppose that $a = \lfloor n\widetilde{a} \rfloor$, for some $0 \leq \widetilde{a} = \alpha \leq 1$ (the notation α in the paper is being changed to $\widetilde{a}$ in this section, to make it more similar to other quantities related to the limit studied in Section S6; $\lfloor y \rfloor$ is the largest integer smaller than or equal to y, i.e., the integer part of y). Then, when n is large, we have in good approximation (in the sense of (S21)) $v_k^A = \widetilde{v}_{k/n}^A$, where $\widetilde{v}_x^A = -C + Bx$, for $0 \leq x \leq \widetilde{a}$, and $\widetilde{v}_x^A = T\,(-C + Bx)$, for $\widetilde{a} < x \leq 1$. In case selection is weak and n is large the viability condition is obtained from setting $\Delta p > 0$ in (A3). Integration of the right-hand-side of (A3) gives then the following equation for the critical level of relatedness $\widetilde{R}_s^0 = R$.

$$C - BR = (T-1)\left\{BR + \frac{B\widetilde{a} - C}{1-\widetilde{a}}\right\}(1-\widetilde{a})^{1/R}, \tag{S27}$$

except in the trivial case $\widetilde{a} = 1$, in which the right hand side of (S27) is 0. This case corresponds to $a = \widetilde{a}\,n = n$, so that types A cooperate only in the first round. Obviously then, the game reduces to the one-shot public goods game, and indeed, (S27) reduces to the usual condition $C = BR$.

In the opposite extreme, when $\widetilde{a} = 0$, so that $a = \widetilde{a}\,n = 0$ and types A cooperate in each round of the game, the right hand side of (S27) reduces to $(T-1)\,(C - BR)$. So (S27) is again reduced to $C = BR$, as one should expect.

Note that, obviously, also when $T = 1$, the game is a one-shot public goods game and (S27) reduces once more to $C = BR$. One can see the right hand side of (S27), as a correction to that viability condition in the more general and challenging example that we are analyzing here.

Analysis of (S27). Results: While somewhat intimidating at first sight, (S27) provides good information about the critical value $R = \widetilde{R}_s^0$, that solves it. We first state the main features of its behavior, and then explain in the next subsection how to do the computations

leading to these claims. Of special biological and mathematical importance is the case in which $\widetilde{a} = C/B = \min\{0 \le x \le 1 : \widetilde{v}_x^A \ge 0\}$. In this case, in which types A are payoff dependent conditional cooperators, (S27) simplifies to:

$$C - BR = (T-1)\,BR\,(1 - C/B)^{1/R}. \tag{S28}$$

We will see that with C, B and T fixed, $\widetilde{R}_s^0$ is a continuous function of $0 \le \widetilde{a} \le 1$, that takes the value C/B on both end-points of this domain, is strictly decreasing when $0 \le \widetilde{a} \le C/B$, and strictly increasing on $C/B \le \widetilde{a} \le 1$. It reaches therefore its minimum at $\widetilde{a} = C/B$, where it solves (S28). It is intuitive that $\widetilde{R}_s^0$ should reach its minimal value when $\widetilde{a} = C/B$, since then altruists are persisting precisely when it is in their benefit to do so. Fig. S21 compares the solution of (S27), as a function of $\tilde{a}$, with values of R_s^0 obtained from (A2), for $n = 20$ and $n = 100$. Note the features described above present in this pictures.

With T and $\widetilde{a}$ fixed, $\widetilde{R}_s^0$ is a decreasing function of B/C, that goes to 0 as $B/C \to \infty$ (see Fig. S22). With C, B and $0 < \widetilde{a} < 1$ fixed, $\widetilde{R}_s^0$ is a decreasing function of T that behaves as follows when $T \to \infty$. If $0 \le \widetilde{a} < C/B$, then $\widetilde{R}_s^0 \to (C - B\widetilde{a})/(B - B\widetilde{a})$, while if $C/B \le \widetilde{a} \le 1$, then $\widetilde{R}_s^0 \to 0$. In this latter case, the convergence is rather slow, in that $\widetilde{R}_s^0$ behaves asymptotically as $(\log(1/(1-\widetilde{a})))/\log(T)$. (See Fig. S23.)

Analysis of (S27). Computations: The claims above follow from the behavior of the left hand side and the right hand side of (S27). We denote them respectively by $H(R) = H_{C,B}(R)$ and $G(R) = G_{C,B,T,\widetilde{a}}(R)$. (With $G(0) = 0$, so that G is continuous on the interval $[0,1]$. Note that not only $G(R)$, but also all its derivatives converge to 0 as $R \to 0$.) See Fig. S24, for an illustration of what follows. The function $H(R)$ is very straightforward; it is a strictly decreasing function of R, that is positive for $R < C/B$ and negative for $R > C/B$. The function $G(R)$ has the sign of the term inside the curly braces. That term inside the curly braces is 0 when $R = (C - B\widetilde{a})/(B - B\widetilde{a})$. We define $\widehat{R} = \max\{(C - B\widetilde{a})/(B - B\widetilde{a}), 0\}$, and observe that $0 < \widehat{R} < C/B$, when $\widetilde{a} < C/B$, and $\widehat{R} = 0$, when $\widetilde{a} \ge C/B$. The term inside the curly braces, and therefore also $G(R)$, is negative for $R < \widehat{R}$, and positive for $R > \widehat{R}$. Notice also that once it is positive, $G(R)$ is strictly increasing in R, since it is then the product of strictly increasing positive functions. The behaviors described so far for $H(R)$ and $G(R)$ immediately imply that they are equal to each other at exactly one point $R = \widetilde{R}_s^0$, and that this point is in the open interval $(\widehat{R}, C/B)$.

The various claims about the behavior of $\widetilde{R}_s^0$ as a function of $\widetilde{a}$, or of B/C, or of T, follow now from analyzing the behavior of the graphs of $H(R)$ and of $G(R)$, as these parameters change. We have $\partial G_{C,B,T,\widetilde{a}}(R)/\partial\widetilde{a} = (T-1)\,(C - B\widetilde{a})\,((1/R) - 1)\,(1-\widetilde{a})^{(1/R)-2}$, which, regardless of the value of R, is positive for $0 < \widetilde{a} < C/B$ and negative for $C/B < \widetilde{a} < 1$. This means that the graph of $G(R)$ moves upwards, as $\widetilde{a}$ increases from 0 to C/B, and then moves downwards, as $\widetilde{a}$ increases from C/B to 1. In the extremes, $G(R) \to (T-1)(BR-C)$, as $\widetilde{a} \to 0$, for all $R > 0$. (This convergence is not uniform, since the function $G(R)$ converges to 0 as $R \to 0$.) And $G(R) \to 0$, uniformly in R, as $\widetilde{a} \to 1$. These facts, and the

trivial behavior of $H(R)$ that does not depend on $\widetilde{a}$, provides us with the facts about the dependence of $\widetilde{R}_s^0$ on $\widetilde{a}$.

The fact that $\widetilde{R}_s^0$ depends on C and B only through B/C can be seen by dividing both sides of (S27) by C. The fact that $\widetilde{R}_s^0$ decreases as B/C increases follows easily from observing that the graphs of $H(R)$ and $G(R)$ move, respectively, down and up, as B increases, with C fixed. And the fact that $\widetilde{R}_s^0 \to 0$, as $B/C \to \infty$, is immediate from $0 < \widetilde{R}_s^0 < C/B$ (Fig. S22).

If we keep B, C and $0 < \widetilde{a} < 1$ fixed and let $T \nearrow \infty$, then $G(R)$ also goes monotonically to ∞, for $\widehat{R} < R < C/B$, and stays at 0 for $R = \widehat{R}$. The corresponding behavior of the graph of $G(R)$, and the fact that $\widetilde{R}_s^0$ is the point in $\widehat{R} < R < C/B$ where this graph intersects the graph of the decreasing function $H(R)$, shows that, as claimed above, $\widetilde{R}_s^0$ is decreasing in T, and as $T \to \infty$, $\widetilde{R}_s^0 \to \widehat{R}$ (Fig. S25). The claim about the slow speed of this convergence, in case $C/B \leq \widetilde{a} < 1$, can be obtained by taking the logarithm of both sides of (S27) and analyzing how the resulting terms behave as $T \to \infty$.

Analysis of (S28): For each value of T, this equation is a relationship between the values of C/B and R, since it can be written as $C/B - R = (T-1)\, R\, (1-(C/B))^{1/R}$. Fig. S26 shows how this relationship looks like. For $T = 1$ we have $R = C/B$, and as T increases, R decreases towards 0, for each value of $0 < C/B < 1$. To obtain an approximation for R, when T is large, we observe that, if we define $\varphi = C/BR$, then (S28) can be written

$$\varphi - 1 \; = \; (T-1) \, \left[(1-C/B)^{B/c}\right]^{\varphi} . \qquad \text{(S29)}$$

Taking logarithms (arbitrary base) from both sides, we get

$$\frac{\log(\varphi - 1)}{\varphi} \; = \; \frac{\log(T-1)}{\varphi} \; + \; \frac{B}{C} \log\left(1 - \frac{B}{C}\right) . \qquad \text{(S30)}$$

Suppose $0 < C/B < 1$ fixed, and let $T \to \infty$. Then (S29) implies that $\varphi \to \infty$, since otherwise the right-hand-side would go to ∞, while the left-hand-side would not. But since $\varphi \to \infty$, the left-hand-side of (S30) goes to 0, and hence

$$\frac{\log(T-1)}{\varphi} \; \to \; -\frac{B}{C} \log\left(1 - \frac{B}{C}\right), \qquad \text{or, equivalently,} \qquad R \log T \; \to \; -\log\left(1 - \frac{B}{C}\right).$$

This provides us with the approximation (A5), when T is large.

A note on the assumptions in [3], and some intuition on our results: It is important to explain why the assumptions made in [3] on how much assortment to expect in groups, and later used in several papers, including [2], are excessively pessimistic. In [3] it was supposed that conditioned on the focal individual being type A, the other $n-1$ members of the focal group would be type A independently, with probability $I\!P_t(A_2|A_1)$. In the 2lFW, conditioned on the focal being type A, if we also condition on the co-focal being type A further increases the conditional probability that a third member of the group is

type A. Conditioning then on a third individual being type A, again further increases the probability that a fourth individual is type A, and so on. This is so because the information being successively provided keeps increasing the probability that there were several types A in the previous generation in the group from which the focal descends.

It is very interesting to make the computation of $\widetilde{R}^0_s$ using the assumption of [3] and compare the result with what we have obtained from (S27). Under that assumption of conditional independence, the viability condition for the regime of weak selection would become $\sum_{k=1}^n v_k^A \,\mathrm{bin}(k-1|n-1,R^0) > 0$. Define now $\widetilde{v}_x^{A*}$ as $\widetilde{v}_x^A$, when x is a continuity point of $\widetilde{v}_x^A$, and as the average between the limits of $\widetilde{v}_x^A$ from the left and from the right at x, at the possible discontinuity point. Then, by a central limit theorem, in the limit (S18),

$$\sum_k v_k^A \,\mathrm{bin}(k-1|n-1,R^0) \quad\longrightarrow\quad \widetilde{v}_{R^0}^{A*}.$$

We would then have $\widetilde{v}_{\widetilde{R}^0_s}^{A*} = 0$. The only solution of this equation is $\widetilde{R}^0_s = C/B$, regardless of the value of $\widetilde{a}$. This result would not depend on T, and would differ substantially from our result in case, e.g., $\widetilde{a} = C/B$, T large, for which (A5) holds.

To understand the reason for those substantially different results for the IPG in the 2lFW framework and in the ad hoc conditional independence scenario of [3], we observe the following. In the 2lFW, when $\delta = 0$, n is large and m is small, the fraction of individuals in the focal group who are IBD to the focal individual is well approximated by a beta distribution with mean R and standard deviation $R\sqrt{2/(R+1)}$. In contrast, under the assumption of [3] that fraction is well approximated by a normal distribution with mean R and standard deviation $\sqrt{R(1-R)/n}$. So, in the 2lFW framework, the distribution has a spread that is independent of n and is comparable and even larger than its mean and has a heavy tail. (See (S19) and Fig. S19. For instance, in this figure, when $m = 0.1$, we have $R^0 = 0.2$ in panel A and $R^0 = 0.04$ in panel B. But see how much probability the beta distribution allows for substantially larger values of k/n). In the scenario of [3], when n is large, the distribution is narrowly concetrated around the same mean, with a light Gaussian tail. The conditionality of behavior in IPG makes the fitness function v_k^A grow fast when k is large (see Fig. S1) and gives a fundamental role to the tail of that distribution, explaining the discrepancy.

This comparison with [3] highlights some fundamental facts about assortment of genes under weak selection in 2lFW and also in Wright's infinite island model, since both have the same coalescent. The number $K^D - 1$ of individuals in a focal group that are IBD to and distinct from a focal individual in this group is a random variable that does correspond to $n-1$ attempts, each with probability R^0 of success. But, contrary to the assumption in [3], these $n-1$ attempts are highly dependent, so that we do not get a good Gaussian (central limit theorem) approximation, but rather a good beta distribution approximation, with a fatter tail. The observation of this lack of normality and the identification of the appropriate approximate distribution as a beta are central contributions of the current work, with powerful computational and conceptual implications.

When are types A altruistic in the IPG? There is more than one way in which the concept of altruism in the context of the trait-group framework has been defined [10]. These different definitions carry over to the 2lFW. A particularly simple concept is called in [10] the "multilevel interpretation" of altruism. That definition requires the two conditions $w_k^A < w_k^N$, for all k, and $\bar{w}_k$ increasing in k. The first one means that types A are always worse off than types N in the same group, and the second one means that the more types A in a group, the better for the group. Both conditions are clearly always satisfied in the IPG, that has $\bar{w}_k = (B - C)k/n$, if $k \leq a$ and $\bar{w}_k = T(B - C)k/n$, if $k > a$.

There are nevertheless good arguments for considering other definitions of altruism [10]. A particularly appealing definition is called "focal-complement interpretation" in [10], and is often known as "mutation condition". Suppose that a type N mutated into a type A, everything else remaining unchanged. Would this cause a decrease in the fitness of the mutant? Would it cause an increase in the average fitness of the other members of the mutant's group? Since the average fitness of the n members of the mutant's group increases with the mutation, the answer to the second question will be affirmative whenever the answer to the first question is affirmative. Therefore the condition for types A to be altruistic is that

$$v_{k+1}^A - v_k^N \ < \ 0, \tag{S31}$$

for all k. The l.h.s. of (S31) takes the values: $v_{k+1}^A - v_k^N = -C < 0$, if $k \leq a - 1$; $v_{k+1}^A - v_k^N = -CT < 0$, if $k > a$; and $v_{a+1}^A - v_a^N = .T(-C + aB/(n-1)) - aB/(n-1)$, if $k = a$. Therefore, only the case $k = a$ needs to be analyzed. When $T = 1$ and the IPG is identical to the PG, (S31) holds. For arbitrary values of T, the same is true for $a = 0$ (again, the IPG is identical to a PG, in this case with values of C and B multiplied both by T). For each value of T and n, (S31) holds for values of a that are small and may start failing at some critical value of a. In case T is large, that critical value of a is close to $(n-1)C/B$, that, when n is also large, corresponds to $\alpha = C/B$. The intuition for this critical value of a is as follows. When $k \neq a$, it is always costly for a type N to mutate into a type A, since that mutation will not change the number of times other types A in the group cooperate, and will cost to the mutant a net amount C for each act of cooperation in the group. But in case $k = a$, the mutation changes the group from being one in which cooperation occurs only once, into one in which cooperation occurs an average of T times. When T is large, this is beneficial for the mutant when the net benefit obtained from each one of the many additional acts of cooperation, $-C + aB/(n-1)$, is positive, but is detrimental otherwise. In case $a > (n-1)C/B$, the mutation can therefore benefit the mutant in the long run. In this case, types A are not altruistic when in groups with a total of $a + 1$ types A. Indeed, their acts of cooperation are then needed to keep cooperation going in their group, and therefore the initial such acts increase the fitness of the actor in the long run (life-cycle). In contrast, when $a \leq (n-1)C/B$ (as in Fig. 2 in the paper, where $n = 50$, $C/B = 1/3$ and $a = 16$), all the types A are altruistic, even those in groups with a total of $a + 1$ types A. It is still true that their acts of cooperation are needed to keep cooperation going, but, while this benefits their group, it comes then at a net cost (or at least no gain) per action for each type A individual.

It is known from [12] (see also [10]) that when (S31) holds, the 2lFW with $m = 1$ (which is identical to the trait-group population structure, i.e., random assortment in groups) has no stable equilibrium with types A present. This is therefore the case of the model in Fig. 2 in the paper. It is only through the effects of limited dispersal (population viscosity) and the resulting assortment of types A that this altruistic allele can proliferate in the population.

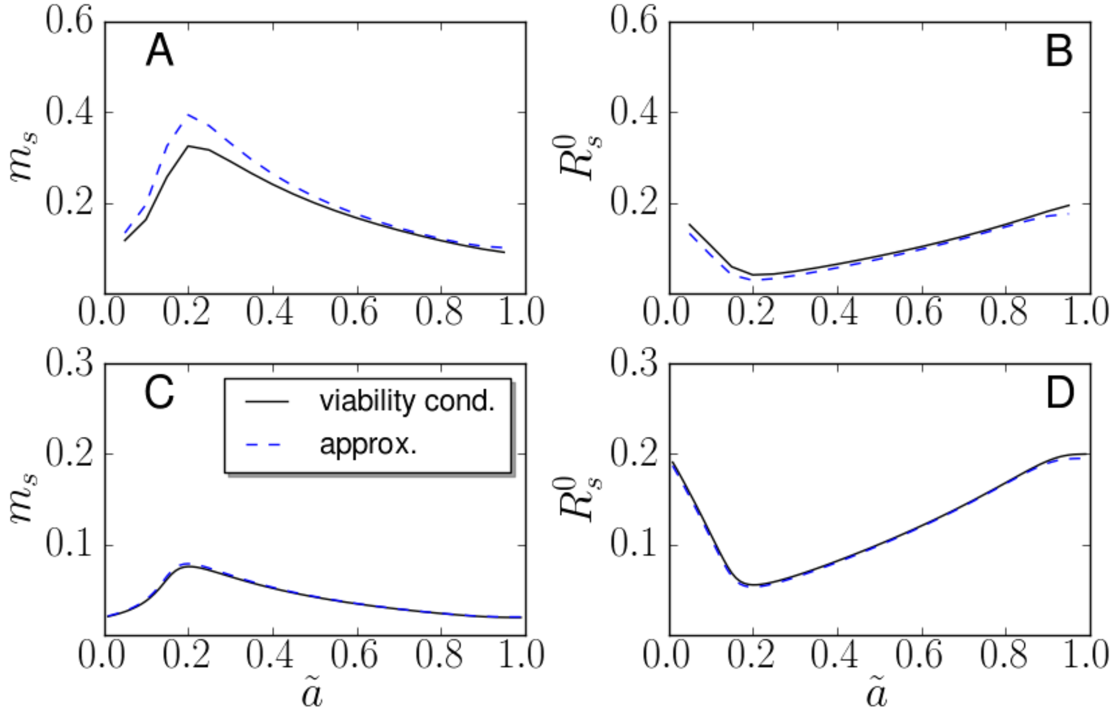

Figure S21: Limit of large n under weak selection for the Iterated public goods (IPG) game (Example 2). Panels represent critical migration rates (A and C) and critical relatedness (B and D) for the IPG with $C = 1$, $B = 5$ and $T = 100$ as a function of $\tilde{a} = a/n$. Top panels A and B depict the case $n = 20$. Bottom panels C and D depict the case $n = 100$. In each panel critical values obtained by the viability condition under weak selection derived from (A2) (black full lines) are compared with the approximation for large n given by solving (S27) in R (approx., dashed blue lines). In panel B we have $R_s^0 = 4.02\%$ when $\tilde{a} = 20\%$, and in panel D we have $R_s^0 = 5.54\%$ when $\tilde{a} = 20\%$. Types A are altruistic in the strong sense of (S31) when $0 \leq \tilde{a} \leq 20\%$.

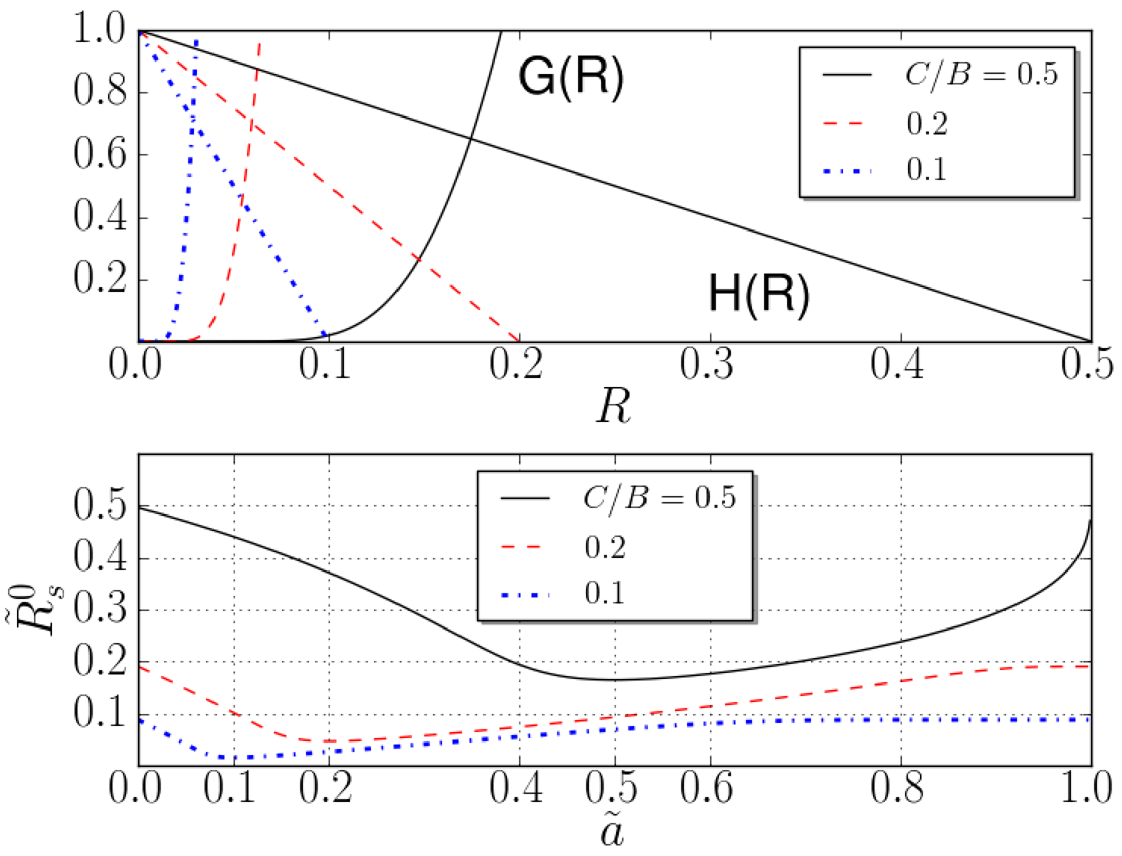

Figure S22: Limit of large n under weak selection for the Iterated public goods (IPG) game (Example 2): behavior of solutions for (S27) - Part 1. Top panel: $H(R)$ corresponds to the l.h.s. of (S27) while $G(R)$ depicts the r.h.s. of (S27). $H(R)$ is strictly decreasing and it is positive for $R < C/B$. Derivatives of $G(R)$ converge to 0 as $R \to 0$. $H(R)$ and $G(R)$ are equal to each other at exactly one point $R = \tilde{R}_s^0$ that is a decreasing function of C/B. Curves depicted correspond to the cases $C/B = 0.5$ (full black line), $C/B = 0.2$ (dashed red line) and $C/B = 0.1$ (dot-dashed blue line) with $\tilde{a} = C/B$ and $T = 100$. Bottom panel: $\tilde{R}_s^0$ as a function of $\tilde{a}$ for $C/B = 0.5$ (top, full black line), $C/B = 0.2$ (middle, dashed red line) and $C/B = 0.1$ (bottom, dot-dashed blue line) and $T = 100$. $\tilde{R}_s^0$ is continuous in the interval $0 \leq \tilde{a} \leq 1$, takes the value C/B on both end-points of this domain and has a minimum at $\tilde{a} = C/B$.

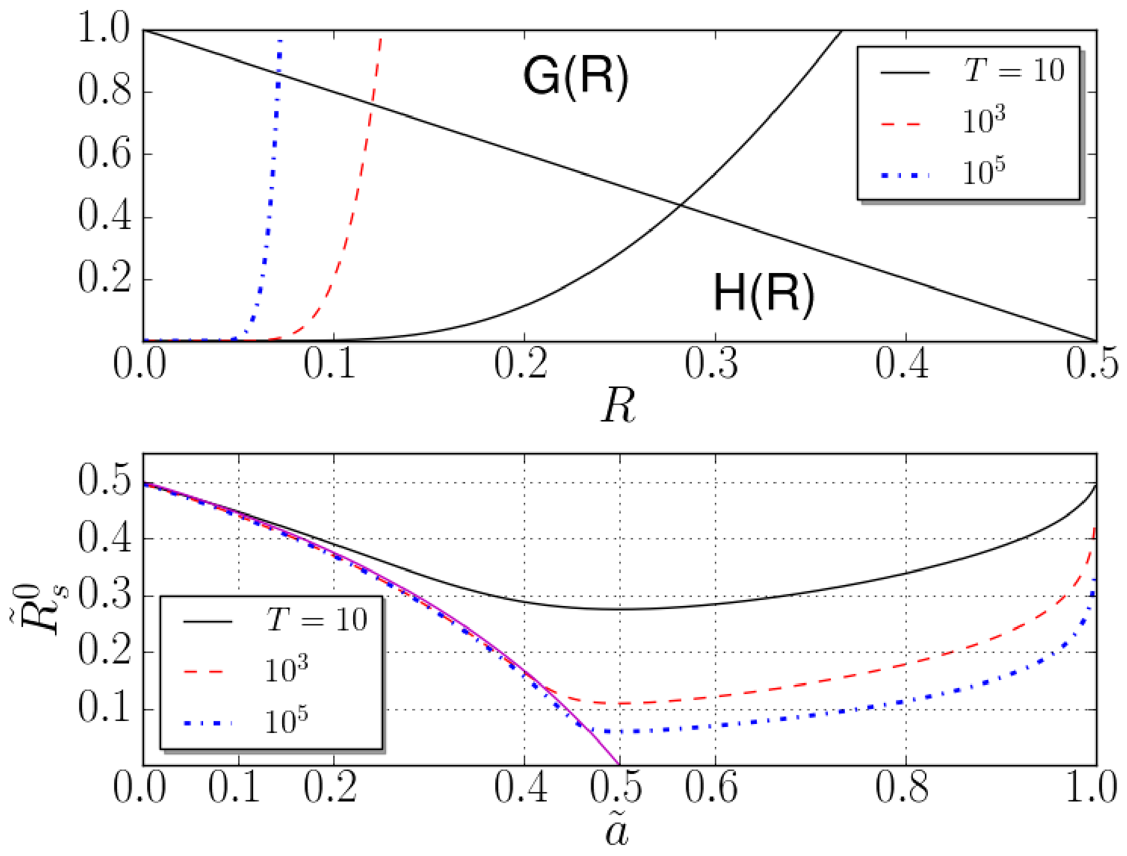

Figure S23: Limit of large n under weak selection for the Iterated public goods (IPG) game (Example 2): behavior of solutions for (S27) - Part 2. Top panel: $G(R)$ and $H(R)$ for $C/B = 0.5$, $\tilde{a} = 0.5$ and $T = 10$ (leftmost, full black line), $T = 10^3$ (dashed red line) and $T = 10^5$ (dot-dashed blue line). $\tilde{R}_s^0$ is a decreasing function of T. Bottom panel: in the limit $T \to \infty$, if $0 \le \tilde{a} < C/B$ then $\tilde{R}_s^0 \to \frac{C/B-\tilde{a}}{1-\tilde{a}}$ (full magenta line). If $C/B \le \tilde{a} \le 1$ then $\tilde{R}_s^0 \to 0$ very slowly.

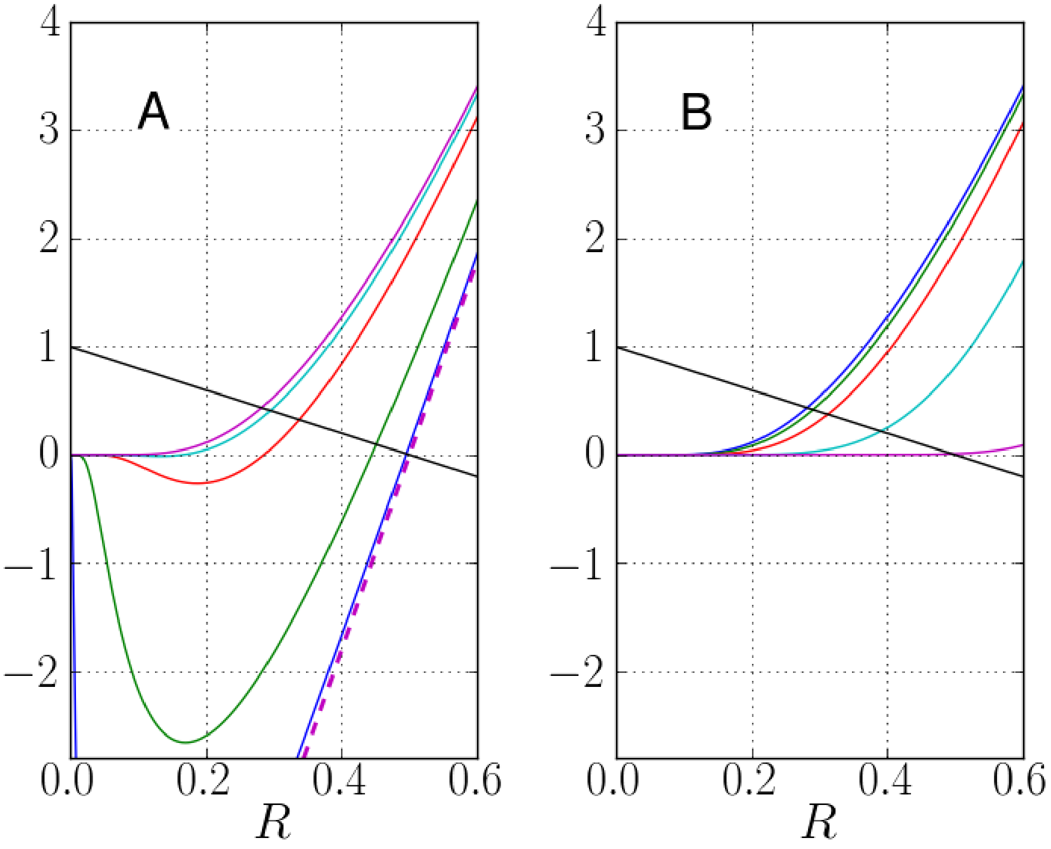

Figure S24: Limit of large n under weak selection for the Iterated public goods (IPG) game (Example 2): behavior of solutions for (S27) - Part 3. $H(R)$ (strictly decreasing straight line) and $G(R)$ for $C/B = 0.5$ and $T = 10$ for $\tilde{a} = 0.01, 0.1, 0.3, 0.4, 0.5$ from right to left in Panel A and for $\tilde{a} = 0.5, 0.6, 0.7, 0.9, 0.999$ from left to right in Panel B. The graph of $G(R)$ moves upwards for $0 \leq \tilde{a} < C/B$ and downwards for $C/B \leq \tilde{a} \leq 1$. $G(R) \to (T-1)(BR - C)$ as $\tilde{a} \to 0$ (dashed magenta line in Panel A). In Panel B it can be seen that $G(R) \to 0$ as $\tilde{a} \to 1$.

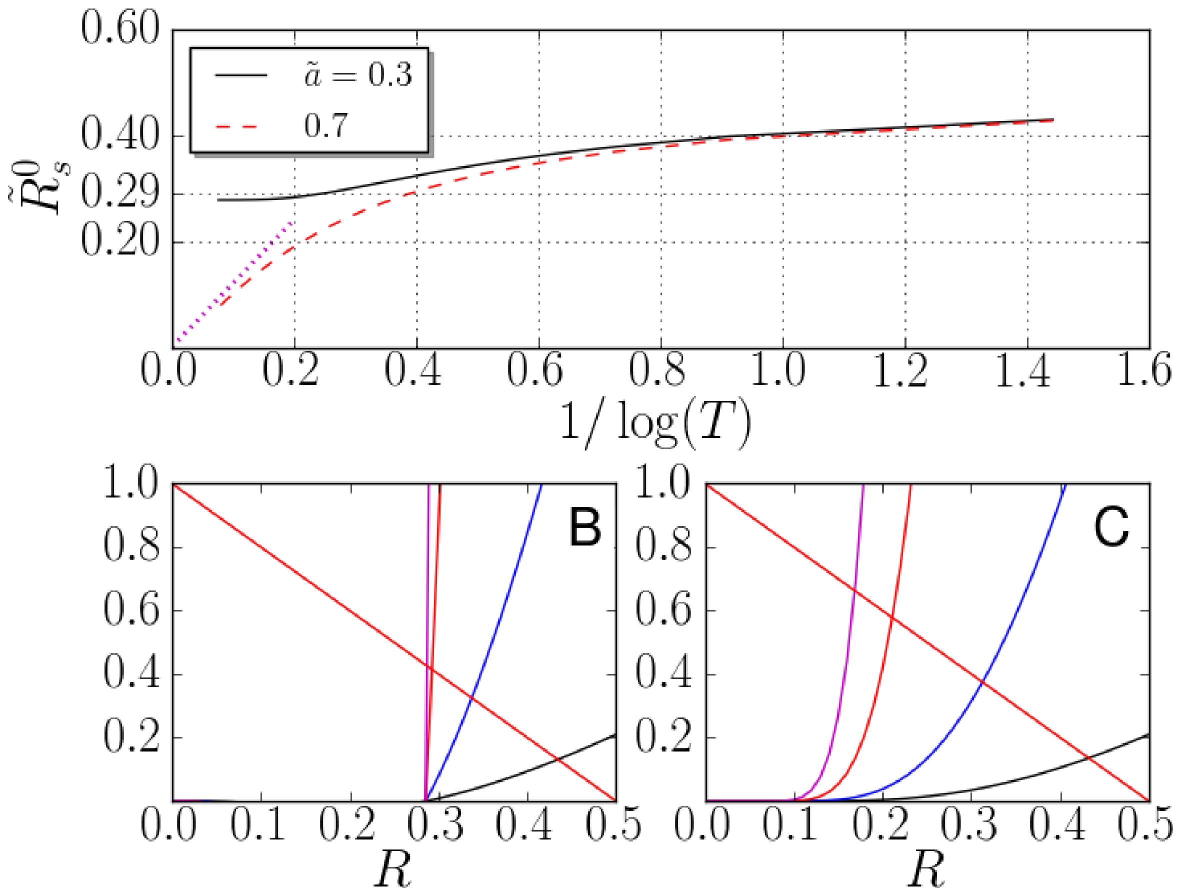

Figure S25: Limit of large n under weak selection for the Iterated public goods (IPG) game (Example 2): behavior of solutions for (S27) - Part 4. In all panels $C/B = 0.5$. Panel A depicts $\tilde{R}_s^0$ as a function of $1/\log(T)$ for $\tilde{a} = 0.3$ (full black line) and for $\tilde{a} = 0.7$ (dashed red line). For $0 \leq \tilde{a} < C/B$ $\tilde{R}_s^0 \to \frac{C/B-\tilde{a}}{1-\tilde{a}}$ (this value is approximately 0.286 for the case shown). If $C/B \leq \tilde{a} \leq 1$ then $\tilde{R}_s^0$ converges to 0 very slowly as T increases, more specifically $\tilde{R}_s^0 \sim -\log(1-\tilde{a})/\log(T)$ (dotted magenta line). Bottom panels show the behavior of $G(R)$ as T increases. Panel B: case $\tilde{a} < C/B$ for, from right to left, $T = 2, 10, 100, 500$. Panel A: case $\tilde{a} > C/B$ for $T = 2, 10, 100, 500$, from right to left. $G(R)$ stays at zero for $\hat{R} = \max\{\frac{C/B-\tilde{a}}{1-\tilde{a}}, 0\}$ and goes monotonically to infinity for $\hat{R} < R < C/B$.

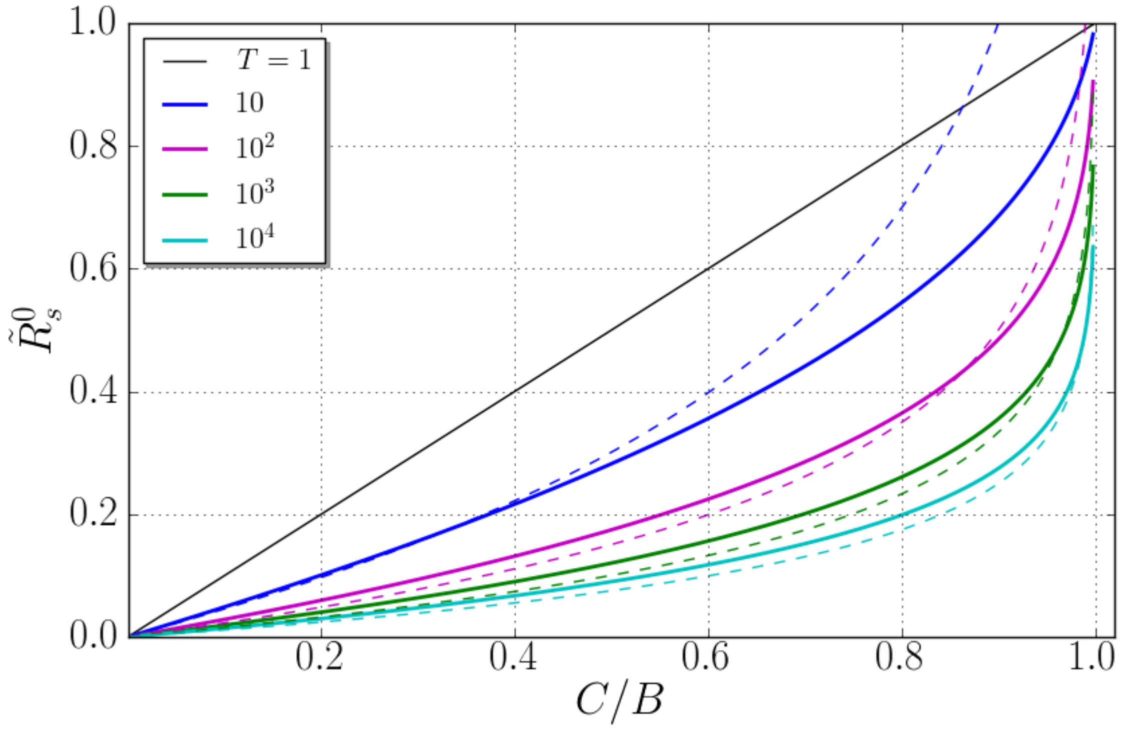

Figure S26: The solid lines provide the solution $\widetilde{R}_s^0$ of $C/B - R = (T-1)\,R\,(1-(C/B))^{1/R}$, as a function of C/B, for (top to bottom) $T = 1$ (black), 10 (blue), 100 (magenta), 1000 (green) and 10000 (cian). The corresponding dashed lines with same colors (no black one) provide the approximation (A5), $R = -\ln(1-(C/B))/\ln T$. This figure is an expanded version of Panel C of Fig. 2 in the paper.

S9 Time periods, mutations, viability conditions, covariance-regression formulas

Time periods: When $p(0) = p << 1$, the evolution is well described by the linearized form $f(t+1) = f(t)M(A+B)$, until $p(t)$ is no longer small. We refer to the time period while the linearized evolution is a good approximation as the "early stage", E.S., as opposed to the "late stage", L.S., when the evolution of $f(t)$ can no longer be well approximated in this linear fashion. During the E.S., we have, from (S1), $f(t) = C\rho^t(\nu + e(t))$, where the error term $e(t)$ satisfies $\max_i |e_i(t)| < \beta^t$, with $0 \leq \beta < 1$, and $C = \sum_k f_k(0)\zeta_k$ is a strictly positive constant, of order p (since all the entries of $f(0)$ must be of order p or smaller). Therefore, we can think of the E.S. as the period of time while $\rho^t << 1/p$, or, equivalently, $t << \log(1/p)/\log\rho$. The E.S. can be further split into the "very early stage", V.E.S., during which the error term $e(t)$ is still relevant, and the "stationary early stage", S.E.S., characterized by $\max_i |e_i(t)| << 1$, so that $f(t) = C\rho^t\nu$ in good approximation. The S.E.S. can be thought as the period of time $1 << t << \log(1/p)/\log\rho$. Thruought the E.S. we have

$$\Delta p(t) \; = \; p\,\delta\,\frac{\sum_{k=1}^n k f_k(t) v_k^A}{\sum_{k=1}^n k f_k(t)}, \qquad f(t) = f(0)[M(A+B)]^t. \tag{S32}$$

But it is only in the S.E.S., that $\Delta p(t)$ takes the stationary form given by (A1).

In Fig. S10 the V.E.S. is the short time period (about 4 or 5 generations) before $f(t)$ reaches the line spanned by ν. Once $f(t)$ is moving along that line, we are in the S.E.S.. In this figure the evolution is produced by the linearized form $f(t+1) = f(t)M(A+B)$, so that one cannot see the L.S.. Under the non-linear dynamics, $f(t)$ eventually departs from that line, in the L.E..

Small mutation rate: The condition $\rho < 1$ (or $\rho > 1$) implies the local stability (or instability) of the fixed point $(0,...,0)$ of the dynamical system $f(t)$. Stability here can be interpreted with respect to small deviations from that fixed point as initial condition, or in terms of the effect of a small mutation rate μ. For this latter purpose, we introduce mutation at rate $0 < \mu < 1$ per individual per generation, by assuming that each offspring is of the same type of her mother with probability $1-\mu$ and otherwise is of the other type. Suppose that $f(0) = (0,...,0)$ and that μ is small. When types A are rare, the main effect of mutation is to create new types A in groups that have no types A. Therefore, neglecting terms of order μ^2 or smaller, we have, $f(1) = (\mu n, 0,...,0)$, $f(2) = (\mu n, 0,...,0)M(A+B) + (\mu n, 0,...,0)$, ..., $f(s) = \sum_{t=0}^{s-1}(\mu n, 0,...,0)[M(A+B)]^t$. This series converges when $\rho < 1$, and diverges when $\rho > 1$, since its generic term behaves as $C\rho^t\nu$, when t is large. This means that when $\rho < 1$, $f(t)$ converges to the perturbed fixed point $f^\mu \;=\; \mu n\sum_{t=0}^{\infty}(1,0,...,0)[M(A+B)]^t$, in which p is of order μ, and represents a small perturbation of the fixed point $f^0 = (0,...,0)$. And, in contrast, when $\rho > 1$, allele A invades the population, for any mutation rate $\mu > 0$.

Viability conditions: By a *viability condition* for an allele we mean a condition for it to spread when rare. In the 2lFW setting, this means the instability of the fixed point

$f = (0, ..., 0)$, either with respect to a small perturbation of the initial condition or by introducing a small mutation rate μ. Both are expressed by the mathematical condition $\rho > 1$. This is equivalent to the condition $\Delta p > 0$, under no mutation and for small $p(0) = p$, provided that one waits until the S.E.S.. If we consider, say, an iterated public goods game and start from $f(0) = (np, 0, ..., 0)$, (*resp.* $f(0) = (0, ..., 0, p)$), then initially $p(0) = p$ and $\Delta p(t)$ is negative (*resp.* positive), regardless of the value of m. In both cases, in the S.E.S., $f(t)$ becomes parallel to ν and (A1) holds. Fig. S11, Fig. S12 and Fig. S13 illustrate this discussion. The evolution of the distribution $f(t)$ towards multiples of ν may be thought of metaphorically as "natural selection organizing the copies of the allele A in the best way, compatible with the demographics, for them to grow". Alternatively, we can use the metaphor that "the allele A self-organizes itself in the best way, compatible with the demographics, for it to grow". Regardless of the language used, the condition $\Delta p > 0$ is only equivalent to the viability condition in the S.E.S., when the distribution of the copies of the allele A is stationary in a way compatible with natural selection and the demographics. We believe that this lesson, that to obtain viability conditions, one should only make requirements of the type $\Delta p > 0$ under stationarity, had not been fully appreciated before, and is a contribution from the current work.

Our viability condition, in the 2lFW setting, can be written, respectively as

$$\rho > 1, \quad \text{or, equivalently,} \quad \sum_{k=1}^{n} k\nu_k v_k^A > 0, \tag{S33}$$

in general (thanks to (A1)); as

$$\sum_{k=1}^{n} \pi_k v_k^A > 0, \tag{S34}$$

under weak selection (thanks to (A2)); and as

$$\int_0^1 (1-x)^{\frac{1}{R}-2}\, \widetilde{v}_x^A \, dx > 0, \tag{S35}$$

with $R = 1/(1+2mn)$, under the conditions of (A3). In the special case of (S35) in which $\widetilde{v}_x^A = -C + Bx + B_2 x^2 + ... + B_l x^l$, we have the viability condition

$$C < BR + B_2 R_2 + ... + B_l R_l, \tag{S36}$$

where $R_l = l! R^l / [(l-1)R+1)(l-2)R+1) \cdots (R+1)]$. In the special case of (S35) in which we have the THR with $\widetilde{v}_x^A = -C$, for $0 \le x \le \widetilde{\theta}$, and $\widetilde{v}_x^A = -C + A$, for $\widetilde{\theta} < x \le 1$, we have the viability condition (see Section S7)

$$R > \frac{1}{1 + \frac{\log(C/A)}{\log(1-\widetilde{\theta})}}$$

In the special case of (S35) in which we have the IPG with $\widetilde{v}_x^A = -C + Bx$, for $0 \le x \le \alpha$, and $\widetilde{v}_x^A = T(-C + Bx)$, for $\alpha < x \le 1$, we have the viability condition (see Section S8)

$$C - BR < (T-1)\left\{ BR + \frac{B\alpha - C}{1-\alpha} \right\} (1-\alpha)^{1/R},$$

that, when $\alpha = C/B$, simplifies to

$$C \,-\, BR \,<\, (T-1)\,BR\,(1-C/B)^{1/R}.$$

The viability conditions above involve only parameters of the models. These inequalities provide the exact conditions under which the allele A can proliferate in the corresponding mathematical model. It is just natural to see them as counterparts to and generalizations of Hamilton's rule $R > C/B$ for the PG.

Covariance-regression formulas: In the 2lFW framework, the fitness $w_\bullet$ of the focal individual is a function of I_1 (that takes value 1 if the focal is of type A and 0 if it is of type N) and of $\kappa = (I_2 + ... + I_n)/(n-1)$, the fraction of types A among the other members of the focal group. Note that κ describes the social environment of the focal individual. Using (S3), the regression coefficient of κ on I_1 can be seen to be:

$$\beta_{t;\kappa,I_1} \;=\; \frac{\mathrm{Cov}_t(I_1,\kappa)}{\mathrm{Var}_t(I_1)} \;=\; \frac{1}{n-1}\,\frac{\mathrm{Cov}_t(I_1, I_2 + ... + I_n)}{\mathrm{Var}(I_1)} \;=\; \frac{\mathrm{Cov}_t(I_1,I_2)}{\mathrm{Var}_t(I_1)} \;=\; r_t.$$

The following covariance-regression formulas are well known [5], [6]:

$$\bar W(t)\,\Delta p(t) \;=\; \mathrm{Cov}_t(w_\bullet, I_1) \;=\; \mathrm{Var}_t(I_1)\,\beta_{t;w_\bullet,I_1} \;=\; \mathrm{Var}_t(I_1)\,\left(\beta_{t;w_\bullet,I_1|\kappa} \,+\, \beta_{t;\kappa,I_1}\beta_{t;w_\bullet,\kappa|I_1}\right). \tag{S37}$$

Here $\beta_{t;w_\bullet,I_1}$ is defined as the number that, together with a proper choice of the constant α_t, minimizes

$$I\!\!E_t\left\{w_\bullet - (\alpha_t \,+\, \beta_{t;w_\bullet,I_1} I_1)\right\}^2,$$

and $\beta_{t;w_\bullet,I_1|\kappa}$ and $\beta_{t;w_\bullet,\kappa|I_1}$ are defined as the numbers that, together with a proper choice of the constant α'_t, minimize

$$I\!\!E_t\left\{w_\bullet - (\alpha'_t \,+\, \beta_{t;w_\bullet,I_1|\kappa} I_1 \,+\, \beta_{t;w_\bullet,\kappa|I_1}\kappa)\right\}^2.$$

If we define $c_t = -\beta_{t;w_\bullet,I_1|\kappa}$ and $b_t = \beta_{t;w_\bullet,\kappa|I_1}$, then (S37) reads

$$\bar W(t)\,\Delta p(t) \;=\; p(t)(1-p(t))\,(-c_t + r_t b_t)\,, \tag{S38}$$

valid in the 2lFW setting for any model, any initial state $f(0)$ (with no conditions on p) and for all time t. In the case of the public goods game, $w_\bullet = 1 + \delta(-CI_1 + B\kappa)$, and (S38) becomes the well known [7] $\bar W(t)\Delta p(t) = p(t)(1-p(t))\delta(-C + Br_t)$. But the simplicity of this special case hides the fact that for other models the regression coefficients c_t and b_t depend not only on the parameters of the model, but also on the distribution of alleles in the population in generation t.

Admittedly, (S38) is always valid, while (S32) is valid only when $p(0) << 1$, during the E.S.. But for the purpose of deciding in the 2lFW when the allele A can invade the population, (S32) and its form (A1) for the S.E.S. give the answer, while we do not see how to use (S38) for this purpose, without first studying how $f(t)$ evolves. This is so because

c_t, b_t and r_t are regression coefficients associated to the random experiment of choosing the focal individual when the distribution of alleles in the groups is given by $f(t)$. And once the evolution of $f(t)$ has to be studied, one can use (S32) directly, without any need to invoke (S38).

Cannot replace regression coefficients with partial derivatives: A popular procedure for approximating (S38) by something easier to analyse, under weak selection, is the replacement of the regression coefficients $c_t = -\beta_{t;w_\bullet,I_1|\kappa}$ and $b_t = \beta_{t;w_\bullet,\kappa|I_1}$ by partial derivatives of fitness functions with respect to I_1 and κ, respectively. See for instance [16], [6] (Box 6), or [18] (condition (6.7)). (The standard notation involves defining $y = \delta I_1$, $z = \delta\kappa$ and $w(y, z) = w_\bullet$, and expressing (S38) in terms of y and z, rather than I_1 and κ. This would make no relevant difference in the computations below.)

We compute next the critical level of relatedness needed for invasion by allele A in the case of the IPG, using that approximation. For the IPG we have

$$w_\bullet = \begin{cases} 1 + \delta[-CI_1 + B\kappa], & \text{if } (n-1)\kappa + 1 \leq a, \\ 1 + \delta[T(-CI_1 + B\kappa)], & \text{if } (n-1)\kappa + 1 > a. \end{cases}$$

We are interested in analysing (S38) when p is close to 0. In this case the relevant partial derivatives would be taken at $I_1 = \kappa = 0$, and would be $\partial w_\bullet/\partial I_1 = -\delta C$, $\partial w_\bullet/\partial\kappa = \delta B$. Therefore (S38) would become $\Delta p = p\delta(-C + Br_t)$. Then we would conclude that allele A could only invade when δ is small if $R^0 > C/B$. But as we know from Section S8, this is incorrect in a substantial way. Note that the limit $\delta \to 0$ corresponds to the limit of "vanishing trait variation" under which [6] and [18] claim that the replacement of regression coefficients by derivatives, as in the computation above, would give the right condition for invasion (when p is small the condition of "vanishing genetic variation" required in [18] is also met).

References

[1] Archetti, M. and Scheuring, I. (2010) Coexistence of cooperation and defection in public goods games *Evolution* **65**, 1140-11148.

[2] Boyd, R., Gintis, H., Bowles, S. (2010) Coordinated punishment of defectors sustains cooperation and can proliferate when rare. *Science* **328**, 617-620.

[3] Boyd, R. and Richerson, P.J. (1988) The evolution of reciprocity in sizable groups. *Journal of Theoretical Biology* **132**, 337-357.

[4] Durrett, R. (2005) *Probability: Theory and Examples* (Thomson, Brooks/Cole, London).

[5] Frank, S. (1997) The Price equation, Fisher's fundamental theorem, kin selection, and causal analysis. *Evolution* **51**, 1712-1729.

[6] Gardner, A., West, S.A. and Wild, G. (2011) The genetical theory of kin selection. *Journal of Evolutionary Biology* **24**, 1020-1043.

[7] Hamilton, W.D. (1975) Innate social aptitude of man: An approach from evolutionary genetics. In: *ASA Studies 4: Biosocial Anthropology.* Fox, R., ed. (Malaby Press, London). 133-153.

[8] Hauert, C., Michor, F., Nowak, M.A. and Doebeli, M. (2006) Synergy and discounting of cooperation in social dilemmas. *Journal of Theoretical Biology* **239**, 195-202.

[9] Joshi, N.V. (1987) Evolution of cooperation by reciprocation within structured demes. *Journal of Genetics* **66**, 69-84.

[10] Kerr, B., Godfrey-Smith, P. and Feldman, M.W. (2004) What is altruism? *Trends in Ecology and Evolution* **19**, 135-140.

[11] Lehmann, L., Keller, L., West, S. and Roze, D. (2007) Group selection and kin selection: Two concepts but one process. *Proceedings of the National Academy of Sciences of the United States of America* **104**, 6736-6739.

[12] Matessi, C. and Jayakar, S.D. (1976) Conditions for the evolution of altruism under Darwinian selection. *Theoretic Population Biology* **9**, 360-387.

[13] Meyer, C. (2000) *Matrix Analysis and Applied Linear Algebra.* (SIAM).

[14] Rousset, F. (2004) *Genetic Structure and Selection in subdivided populations.* (Princeton University Press. Princeton).

[15] Schonmann, R.H., Vicente, R. and Caticha, N. (2011) Two-level Fisher-Wright framework with selection and migration: an approach to studying evolution in group structured populations. *arXiv:1106.4783.*

[16] Taylor, P.D. and Frank, S.A. (1996) How to make a kin selection model. *Journal of Evolutionary Biology* **180**, 27-37.

[17] van Veelen, M. (2009) Group selection, kin selection, altruism and cooperation: when inclusive fitness is right and when it can be wrong. *Journal of Theoretical Biology* **259**, 589-600.

[18] Wenseleers, T., Gardner, A. and Foster, K.R. (2010) Social evolution theory: a review of methods and approaches. In: *Social Behaviour: Genes, Ecology and Evolution.* Szekely, T., Moore, A.J. and Komdeur, J., eds. (Cambridge University Press, Cambridge, UK) 132-158.
